# CREB1 and GSK3 Isoforms as Potential Adaptive Signaling Nodes in PI3K/Akt/mTOR Inhibitor Treated Philadelphia Chromosome-Positive B-ALL

**DOI:** 10.3390/ijms27188300

**Published:** 2026-09-17

**Authors:** Himanshu Dhanda, Shamsuz Zaman, Sandeep Kumar Swain, Neetu Kushwaha, Raj Kamal, Manpreet Kaur, Bhavika Rishi, Pranay Tanwar, Sufian Zaheer, Amitabh Singh, Sumita Chaudhry, Fouzia Siraj, Aroonima Misra

**Affiliations:** 1 ICMR-Centre for Cancer Pathology, New Delhi 110029, India; himanshudhanda198@gmail.com (H.D.); shamsuz.zaman@icmr.gov.in (S.Z.); 2 Manipal Academy of Higher Education, Manipal 576104, India; 3 ICMR-National Institute of Child Health Research, New Delhi 110029, Indianeetukush1128@gmail.com (N.K.); bhavz83@gmail.com (B.R.); 4 Dr. B.R.A. Institute of Rotary Cancer Hospital, All India Institute of Medical Science, New Delhi 110029, India; pranaytanwar@gmail.com; 5 Vardhman Mahavir Medical College & Safdarjung Hospital, New Delhi 110029, Indiadoc.amitabh@gmail.com (A.S.); sumita_chaudhry@yahoo.co.in (S.C.)

**Keywords:** Ph+ ALL, PI3K/Akt/mTOR pathway, CREB1, GSK3α/β, phospho-kinase array, network pharmacology, biomarkers, drug resistance

## Abstract

Philadelphia chromosome-positive (Ph+) B-cell acute lymphoblastic leukemia (ALL), driven by the BCR-ABL1 fusion, remains highly aggressive, with poor prognosis despite tyrosine kinase inhibitor (TKI) therapy. Aberrant PI3K/Akt/mTOR pathway activation contributes to resistance, warranting identification of predictive kinase biomarkers. To identify and validate critical phospho-kinase markers within the PI3K/Akt/mTOR axis as candidate adaptive signaling nodes and exploratory prognostic markers in Ph+ B-ALL. Network pharmacology using STRING and Cytoscape mapped the protein–protein interactions, identifying high-centrality nodes. Functional enrichment analyses (GO/KEGG) were then performed via ShinyGO. Experimentally, phospho-kinase arrays profiled 39 kinase phosphorylation sites in Ph+ B-ALL cells (SUPB-15) treated with PI3K/Akt/mTOR inhibitors (Rapamycin, GDC-0941, GSK690693, Perifosine). Gene expression was validated by RT-qPCR in 100 Ph+ B-ALL patients and protein validation employed Western blot in 50 patient samples with 15 and five healthy controls, respectively. Network analysis identified CREB1 and GSK3 isoforms as central hub regulators. Phospho-kinase profiling revealed p-CREB (Ser133) phosphorylation upregulation following Perifosine, GDC-0941, and Rapamycin treatment, with no statistically significant change after GSK690693. p-GSK3α/β (Ser21/9) phosphorylation showed a statistically significant increase after Perifosine, with non-significant trends after GDC-0941, and a significant reduction following Rapamycin. Hierarchical clustering then divided proteins into four distinct clusters positioning CREB1 in cluster 3 and GSK3α/β in cluster 2 as candidate adaptive response markers based on their phosphorylation profiles. RT-qPCR demonstrated marked downregulation of GSK3α (approximately 71%), GSK3β (63%), and CREB1 (65%) transcripts in patients versus controls, with even greater suppression seen in cell lines. Conversely, Western blot revealed a significant 2.49 fold elevated p-CREB (Ser133) (**β = 1.317, *p* = 0.000347**) and a phospho-GSK3α/β (Ser21/9) level that did not differ significantly between patients and controls (β = 0.467, *p* = 0.466), revealing mRNA–protein discordance indicative of compensatory post-transcriptional and post-translational regulation. These findings represent treatment-associated and disease-state associations between altered phosphorylation states and the Ph+ B-ALL context; functional validation remains an essential next step. mRNA–protein discordance with transcriptional downregulation (63–71%) contrasting with elevated p-CREB levels (**2.49-fold, *p* = 0.000347**) suggests post-translational compensatory mechanisms under therapeutic pressure. CREB1 and GSK3 isoforms emerge as central network nodes that might potentially mediate adaptive resistance, although functional validation is required. It should be noted that the evidence presented is associative rather than causal and a direct link between the observed phosphorylation changes and clinical drug resistance has not been established in this study. Sustained CREB phosphorylation despite PI3K/Akt/mTOR inhibition implicates alternative kinase pathways (MAPK/ERK, CaMK), supporting rational combination therapy strategies. These findings position p-CREB and p-GSK-3α/β as candidate biomarkers, highlighting the value of phospho-proteomics over transcriptomics alone in capturing pathway activation. Multi-omics integration may prove essential for overcoming adaptive resistance in ALL patients.

## 1. Introduction

Acute lymphoblastic leukemia (ALL) is a hematologic malignancy defined by the uncontrolled clonal expansion and accumulation of immature lymphoid progenitor cells that have undergone malignant transformation and failed to mature properly. These abnormal lymphoblasts invade the bone marrow, peripheral blood, and various extramedullary tissues, where they interfere with normal haematopoiesis and result in potentially life-threatening cytopenias [1]. ALL is one of the most common pediatric cancers, with peak incidence occurring between 1 and 4 years of age, However, the disease is not limited to pediatric populations and also affects adolescents, young adults, and older individuals, in whom clinical outcomes are significantly inferior [1].

The global incidence of ALL is approximately 1.57 cases per 100,000 population, translating to nearly 6000 new diagnoses annually in the United States alone [2,3]. Survival outcomes have improved markedly in pediatric patients, with 5-year overall survival rates now exceeding 90%. Despite this progress, there remains a pronounced disparity associated with age. In patients older than 50 years, long-term survival is achieved in only about 25% of cases, underscoring the ongoing therapeutic difficulties in managing adult ALL [4,5,6]. These differences are further compounded by healthcare access worldwide. There are high survival rates in high-income countries, whereas low- and middle-income countries continue to face substantial challenges, largely due to limited availability of intensive treatment regimens and adequate supportive care [7].

In India, ALL constitutes a major healthcare burden, accounting for 18.22% of leukemia cases in females and 24.63% in males as of 2019, with the disease as the leading cause of disability-adjusted life years (DALYs) and deaths in children and young adults aged 0–20 years [8]. Marked regional variability has been reported across institutions in India, with the proportion of adult ALL among acute leukemia cases ranging from 7.3% to 57.8%. The highest proportions have been observed in South India, where rates range between 54.5% and 57.8%. Despite ongoing advances, treatment outcomes remain less than optimal. Five-year overall survival rates in adults range from 30% to 50%, which is substantially lower than those reported in developed countries. Complete remission rates also show considerable variability, ranging from 46.7% to 91.4%, while overall relapse rates fall between 24.3% and 57.1%, underscoring the urgent need for more effective therapeutic strategies [9,10].

At the molecular level, ALL is characterized by recurrent chromosomal abnormalities and genetic alterations affecting transcription factors, kinases, and epigenetic regulators that drive leukemogenesis and influence prognosis [1]. These molecular characteristics, together with parameters reflecting treatment response, underpin contemporary risk stratification frameworks and have enabled the development of targeted therapeutic approaches, including monoclonal antibodies and CAR-T-cell–based immunotherapies [11,12,13]. Among the various molecular subtypes, Philadelphia chromosome–positive B-cell ALL (Ph+ B-ALL) is recognized as one of the most aggressive and clinically challenging forms. It is defined by the t(9;22)(q34;q11) translocation, which produces the BCR-ABL1 fusion oncogene encoding a constitutively active tyrosine kinase that promotes uncontrolled cellular proliferation, enhances survival, and disrupts normal differentiation processes [14,15].

Ph+ ALL accounts for approximately 20–30% of adult ALL cases and 3–5% of childhood cases, with prognosis remaining strongly age-dependent despite significant therapeutic advances. While pediatric patients now achieve 5-year overall survival rates exceeding 90%, outcomes in older adults remain substantially poor, with survival rates below 25 to 30% [4,5,6,16]. The introduction of tyrosine kinase inhibitors (TKIs), including imatinib, dasatinib and ponatinib, has transformed the treatment landscape, with significant improvements in remission and overall survival [17,18,19]. Nevertheless, treatment resistance and patient relapse remain major challenges, driven by factors such as BCR-ABL1 kinase domain mutations (e.g., T315I) and the activation of alternative signaling pathways that enables leukemic cells to evade therapy [20,21].

Central to ALL pathogenesis and therapeutic resistance is the PI3K/Akt/mTOR signaling pathway, a critical regulator of cell survival, metabolism, and proliferation whose constitutive activation promotes malignant cell growth and contributes to resistance against standard chemotherapy [22,23,24,25,26]. In both pediatric and adult ALL, increased PI3K/Akt/mTOR pathway activity has been consistently associated with poorer prognosis, higher relapse rates, and diminished treatment response [27,28,29]. Dysregulation of this pathway occurs through several mechanisms, including loss of the tumor suppressor PTEN in T-ALL, the presence of the BCR-ABL1 fusion in B-ALL, CRLF2 rearrangements, and activating mutations in upstream kinases such as SYK and PKCδ [30,31,32]. This signaling axis is particularly important in Philadelphia chromosome-like (Ph-like) ALL, where genetic alterations such as CRLF2 rearrangements lead to the activation of downstream pathways, including JAK2/STAT5 and PI3K/Akt/mTOR. These changes drive leukemic proliferation and impair normal B-cell precursor differentiation, contributing to the aggressive clinical course and poor prognosis characteristic of this subtype [22,33,34].

The therapeutic potential of PI3K/Akt/mTOR pathway inhibition has been extensively demonstrated through preclinical studies, with pharmacological inhibition restoring glucocorticoid sensitivity in B-ALL and combinations with tyrosine kinase inhibitors improving outcomes in Ph+ ALL [22,35,36,37]. Second-generation inhibitors that target both mTORC1 and mTORC2 reduce feedback activation of Akt and promote PUM-dependent apoptosis, offering greater efficacy compared to earlier rapalogs [38]. Preclinical evidence also indicates that mTOR inhibition can suppress leukemic growth in high-risk ALL models, with combination strategies involving histone deacetylase inhibitors or dasatinib having synergistic pro-apoptotic effects [39]. However, clinical translation still presents significant challenges, as evidenced by a phase I trial of temsirolimus with intensive chemotherapy that achieved 50% remission rates in relapsed ALL patients but showed excessive toxicity [40].

Despite promising therapeutic potential, there are several challenges in the clinical translation of these findings. Many putative biomarkers identified in studies lack validation in large, independent cohorts and even biomarkers that are statistically significant may not translate into clinically meaningful impacts or influence patient management decisions. Furthermore, kinase activity can occur without mutations, emphasizing the need for comprehensive approaches evaluating protein-level changes and kinase activity patterns rather than relying solely on genetic alterations [41]. The future lies in multi-modal, integrative biomarkers that combine genomics, proteomics, and computational methods including machine learning and artificial intelligence for enhanced predictive and prognostic power [42,43,44,45].

Given that the PI3K/Akt/mTOR pathway already serves as both a central driver of leukemogenesis and an actionable therapeutic target in ALL, carefully designed, evidence-based therapeutic strategies that inhibit this pathway, particularly in combination with conventional gold-standard therapies, hold promise for improving disease outcomes. However, the identification of reliable biomarkers predicting response to PI3K/Akt/mTOR pathway inhibition remains a critical unmet need. The objective of this study is to identify and validate essential markers by evaluating phospho-kinase signaling in PI3K/Akt/mTOR pathway inhibition in Ph+ve ALL. This study also aims to validate said important markers, such as GSK3α/β and CREB1, in clinical samples at both the genetic and proteomic levels, highlighting the differential expression pattern between the two, bringing us closer to real-world clinical utility and addressing the critical gap between preclinical findings and clinical application.

## 2. Results

### 2.1. Network Pharmacology Analysis Identifies CREB1 and GSK3 Isoforms as Highly Connected Nodes in the PPI Network

**Figure 1:**
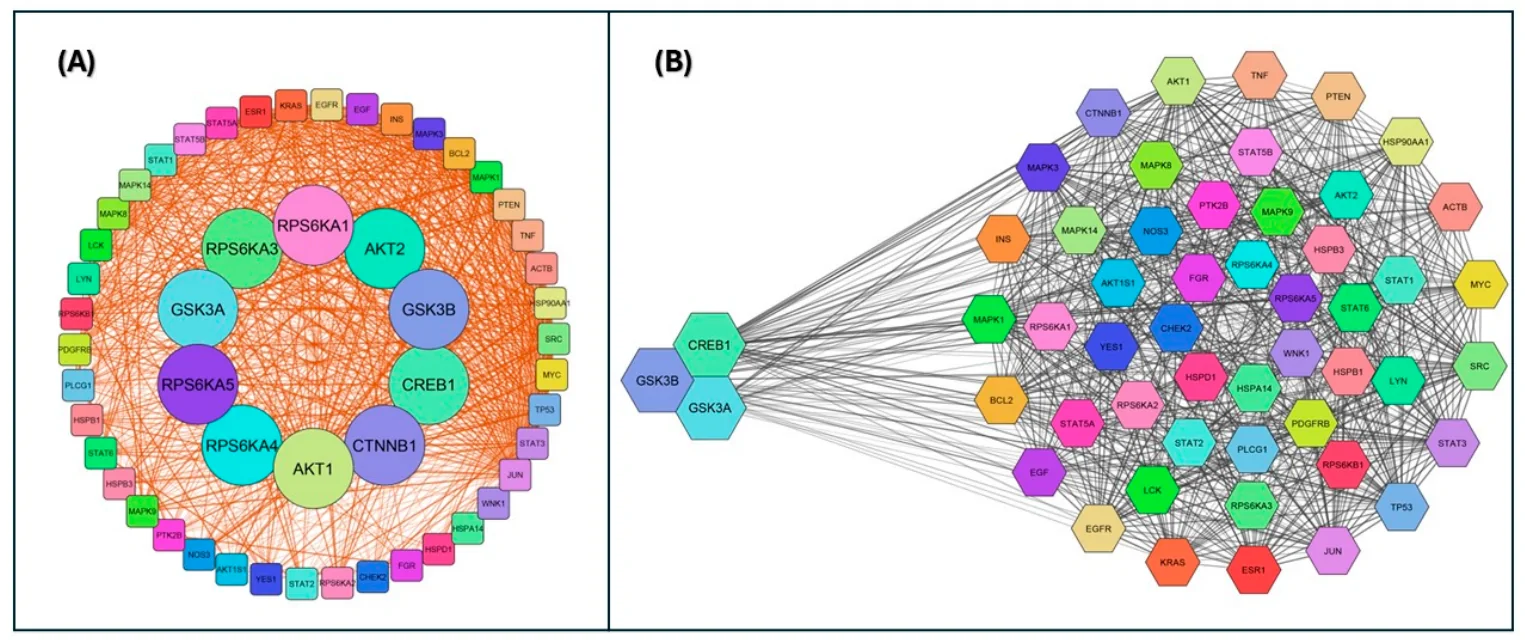
Protein–protein interaction (PPI) and functional network analysis of candidate targets. (**A**). PPI network generated using STRING v12.0 for *Homo sapiens* at a confidence score ≥ 0.4 and visualized in Cytoscape 3.10.3. The network comprised 52 nodes and 767 edges and was derived from the predefined phospho-kinase array panel. CREB1 (degree = 40) and GSK3B (degree = 36) showed connectivity above the network-average degree (29.5), whereas GSK3A (degree = 22) did not meet this criterion. (**B**). Refined visualization highlighting the connectivity of CREB1 and GSK3 isoforms with signaling proteins including AKT1, MAPK1/3, STAT3, TP53, NF-κB, and MYC. CREB1 showed strong interaction with GSK3B, while GSK3A was retained based on its biological relationship to GSK3B and its relevance to the experimentally observed signaling pattern.

The PPI network generated using STRING v12.0 for Homo sapiens at a confidence score ≥0.4 comprised 52 nodes and 767 edges, with one connected component. The network had an average number of neighbors of 29.500, diameter of 3, radius of 2, characteristic path length of 1.431, clustering coefficient of 0.807, density of 0.578, heterogeneity of 0.406, and centralization of 0.336. The proteins analyzed were derived from the predefined phospho-kinase array panel (Proteome Profiler™ Human Phospho-Kinase Array Kit, R&D Systems, Catalog #ARY003C; Section 4.3.2), rather than selected using a hub-detection algorithm. STRING/Cytoscape analysis was used to characterize connectivity among these proteins. For interpretation of network connectivity, nodes with degree ≥ the network-average degree (29.5) were considered highly connected. CREB1 had a degree of 40 (rank 16/52), betweenness centrality of 0.0192, and closeness centrality of 0.823, while GSK3B had a degree of 36 (rank 21/52), betweenness centrality of 0.0066, and closeness centrality of 0.773. Both were above the network-average degree. In contrast, GSK3A had a degree of 22 (rank 37/52), betweenness centrality of 0.0012, and closeness centrality of 0.638, and therefore did not meet this criterion. The complete centrality measures for all 52 nodes are provided in Supplementary Table S1. CREB1 and GSK3α/β were prioritized primarily on the basis of their treatment-associated changes in the phospho-kinase array and their clustering in the hierarchical analysis across the inhibitor conditions (clusters 2 and 3; Figure 1 and Figure 3), with network connectivity providing supportive context. GSK3A was retained alongside GSK3B because of their related biological roles as GSK3 isoforms and their relevance to the experimentally observed signaling pattern. Thus, GSK3A is not considered a topological hub in this analysis. Their interaction partners included major kinases and transcription factors associated with survival and proliferation, including AKT1, MAPK1/3, STAT3, TP53, NF-κB, and MYC, with a strong interaction observed between CREB1 and GSK3 (Figure 1). The STRING interaction scores for CREB1-GSK3A and CREB1-GSK3B were 0.663 and 0.988, respectively.

**Figure 2:**
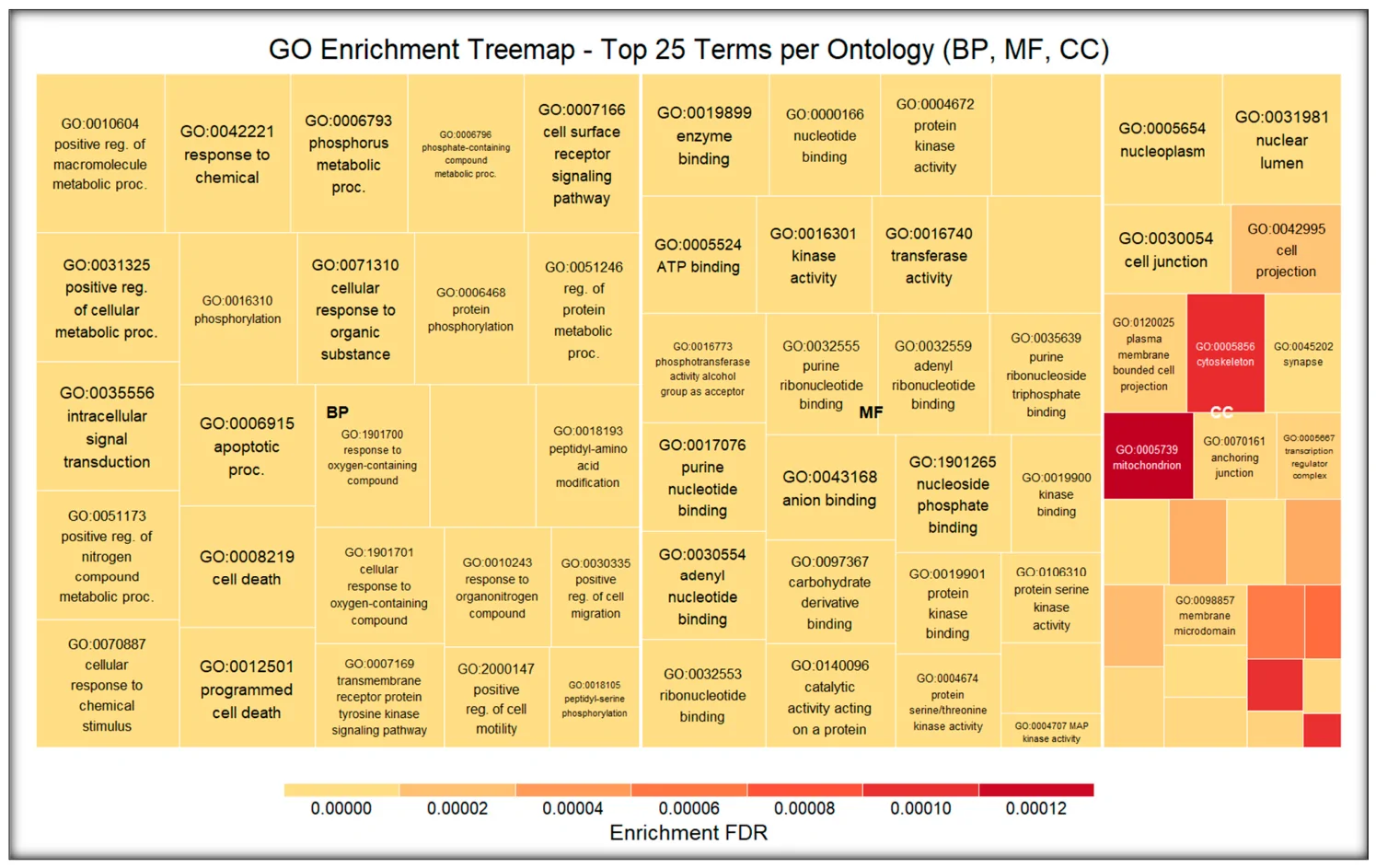
Gene Ontology (GO) enrichment analysis of the target proteins. Top 25 biological process (BP), cellular component (CC), molecular function (MF), and KEGG pathways based on the key targets of T2DM and bioactive compounds in GO term analysis. The color and size of each bubble represent the adjusted *p*-value and gene count, respectively, in all charts.

Gene ontology analysis (FDR < 0.05) identified CREB1, GSK3α, and GSK3β as key targets across 1000 BP, 167 CC, and 190 MF records, with the top 25 enrichments visualized (Figure 2, Supplementary Table S2). GO-BP terms indicated strong associations with cell survival and death, including transmembrane receptor protein tyrosine kinase signaling, protein phosphorylation, and positive regulation of macromolecule metabolic processes, highlighting a significant correlation between CREB and GSK3.

In CC, target genes were enriched in nucleoplasm, nuclear lumen, and cell junctions. CREB1 primarily localizes to the nucleus, regulating transcription via CRE elements, whereas GSK3α/β are mainly cytosolic but shuttle between cytoplasm and nucleus depending on signaling status. MF analysis linked targets to kinase-related activities, including serine/threonine/tyrosine kinase activity and phosphotransferase functions. GSK3 isoforms phosphorylate serine/threonine residues and are negatively regulated by Akt but remain active upon Akt inhibition (e.g., Perifosine).

KEGG pathway analysis (166 enriched pathways, FDR < 0.05) highlighted the PI3K-Akt and mTOR signaling pathways (Supplementary Table S2 and Figure S1). PI3K activation by growth factors (insulin, IGF-1) generates PIP3, recruiting and activating Akt, which phosphorylates GSK3α (Ser21) and GSK3β (Ser9), inhibiting their activity. Akt inhibition (e.g., Perifosine) derepresses GSK3. GSK3 regulates mTORC1 indirectly via activation of TSC2, leading to mTORC1 inhibition, whereas Akt-mediated GSK3 inhibition enhances mTORC1 activity.

mTORC1 promotes protein synthesis and modulates CREB via S6K1 and RSK. Rapamycin-mediated mTOR inhibition reduces CREB phosphorylation and downstream survival gene transcription. In the PI3K–Akt context, Perifosine reduces Akt activity, activates GSK3, and increases CREB phosphorylation, potentially via alternative pathways. In contrast, in mTOR signaling, Rapamycin suppresses CREB activity while GSK3 remains unaffected.

Note: The PPI network was derived from STRING and its topology may be influenced by research and annotation bias, with highly studied and well-annotated proteins more likely to have numerous documented or inferred interactions. The five highest-degree nodes in the present network were JUN (46), TP53 (45), STAT3 (45), SRC (44), and MYC (44). Therefore, network centrality was interpreted as supportive rather than definitive evidence of biological importance.

### 2.2. Phospho-Kinase Array Analysis Reveals Adaptive Kinase Reprogramming Following PI3K/Akt/mTOR Pathway Inhibition

Kinase array profiling was performed to characterize phosphorylation changes in key signaling proteins following inhibition of the PI3K/Akt/mTOR pathway. SUBP-15 cells were treated with Perifosine, GDC-0941, Rapamycin and GSK690693 for 24 h, and phosphorylation status was assessed using a human phospho-kinase antibody array. Statistical analysis was performed for 39 phosphoprotein targets using unpaired two-sided Student’s *t*-tests, with Benjamini–Hochberg FDR correction applied separately within each drug versus untreated control comparison (39 tests per comparison; Q ≤ 0.05).

**Figure 3:**
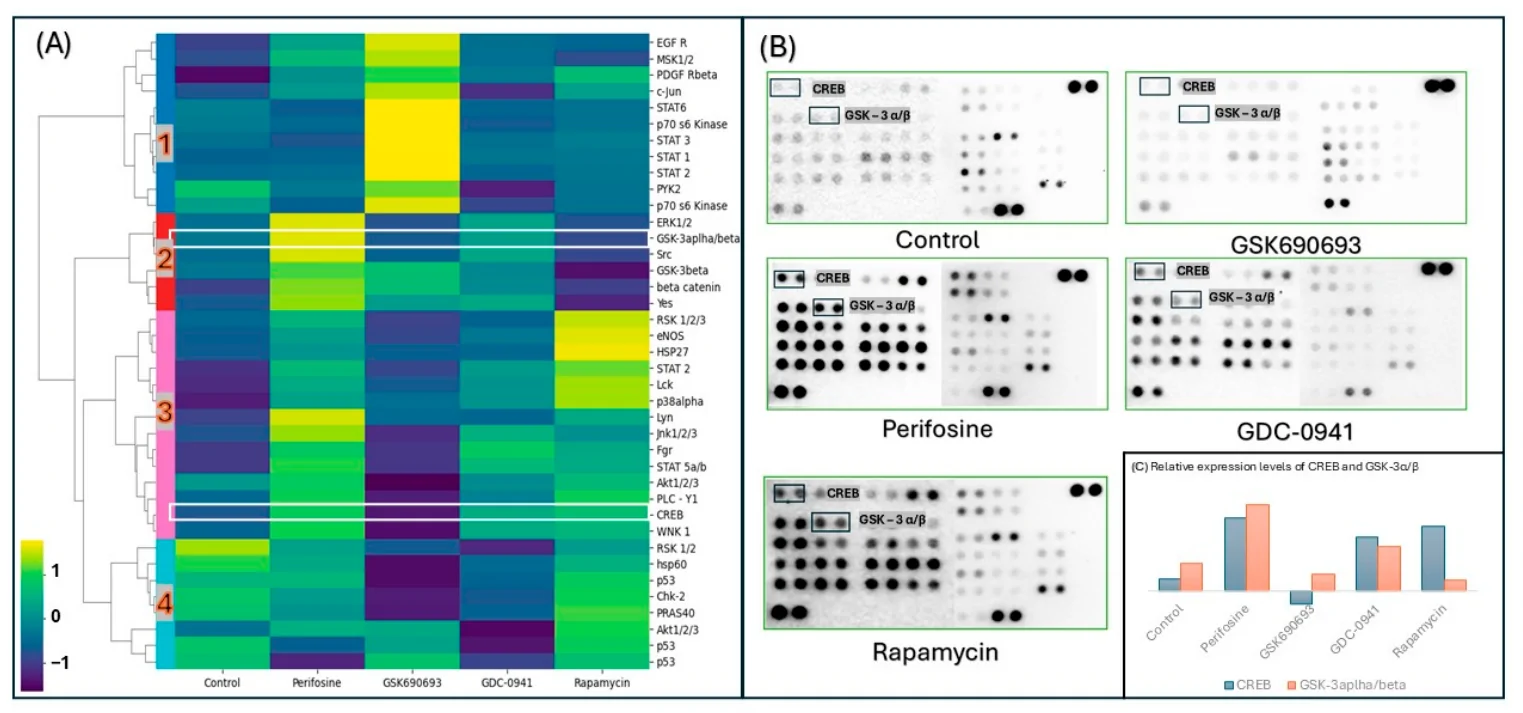
The figure represents a phospho-kinase array profiling of 40 protein targets in untreated SUBP-15 cells versus treated cells (Perifosine, GSK690693, GDC-0941, and rapamycin; inhibitors of the PI3K/Akt/mTOR pathway). (**A**) Heat map representation of protein expression patterns, grouped into four distinct clusters, (**B**) Corresponding membrane array images of the same protein set and (**C**) Relative expression levels of CREB and GSK-3α/β selected as representative markers.

Heatmap-based hierarchical clustering of all detected phosphoproteins grouped the targets into four distinct clusters according to their phosphorylation profiles (Figure 3; Supplementary Tables S3 and S4). These clusters represent descriptive groupings based on z-score-transformed fold-change patterns across treatment conditions and were used to visualize co-regulated phosphorylation profiles rather than as tests of statistical significance. Clusters 2 and 3 predominantly comprised proteins exhibiting altered phosphorylation following Perifosine, GDC-0941, and Rapamycin treatment, whereas clusters 1 and 4 contained proteins displaying comparatively weaker or more heterogeneous responses. Treatment with Perifosine, GDC-0941, and Rapamycin resulted in marked increases in CREB phosphorylation relative to the untreated control. These increases were statistically significant after BH–FDR correction: phospho-CREB (Ser133) increased 6.295-fold following Perifosine (BH-q = 0.033), 4.658-fold following GDC-0941 (BH-q = 0.032), and 5.528-fold following Rapamycin (BH-q = 0.042). No statistically significant change in CREB phosphorylation was observed following GSK690693 treatment (BH-q = 0.070) (Supplementary Figure S2 and Tables S5 and S6).

Distinct phosphorylation patterns were also observed for GSK-3. Perifosine produced a statistically significant 3.123-fold increase in phospho-GSK-3α/β (Ser21/9) relative to the control (BH-q = 0.023), whereas the 1.607-fold increase observed following GDC-0941 did not reach FDR significance (BH-q = 0.287). Similarly, neither the 0.387-fold change following Rapamycin (BH-q = 0.464) nor the 0.592-fold change following GSK690693 (BH-q = 0.236) reached FDR significance for this target. A discrete GSK-3β (Ser9) array entry showed a statistically significant 2.877-fold increase following Perifosine (BH-q = 0.023). Conversely, Rapamycin produced a statistically significant reduction in GSK-3β (Ser9) phosphorylation to sub-background levels in both treated replicates (BH-q = 0.042); because both replicate signals fell below background, this finding is more appropriately described as a significant reduction to undetectable signal rather than as a conventional fold change. GSK-3β (Ser9) phosphorylation did not reach FDR significance following GDC-0941 or GSK690693 treatment.

To further contextualize these phosphorylation patterns, the kinase array dataset was integrated with protein–protein interaction (PPI) network analysis. Among the targets identified, GSK-3α/β (cluster 2) and CREB (cluster 3) displayed high network connectivity together with consistent treatment-associated changes in phosphorylation, identifying them as potential signaling hubs for further investigation. In aggregate, after BH–FDR correction, 19 of 39 targets were significant following Perifosine treatment, 19 of 39 following Rapamycin, 12 of 39 following GSK690693, and 10 of 39 following GDC-0941. The statistically supported alterations in CREB and GSK-3 phosphorylation identify treatment-associated signaling changes warranting further investigation; however, FDR significance in this two-replicate phospho-kinase screen does not by itself establish kinase activation, pathway activation, therapeutic resistance or a causal mechanism.

### 2.3. Statistical Validation by RT-PCR Expression Profiles for GSK3α/β and CREB1 in 100 Patients

**Figure 4:**
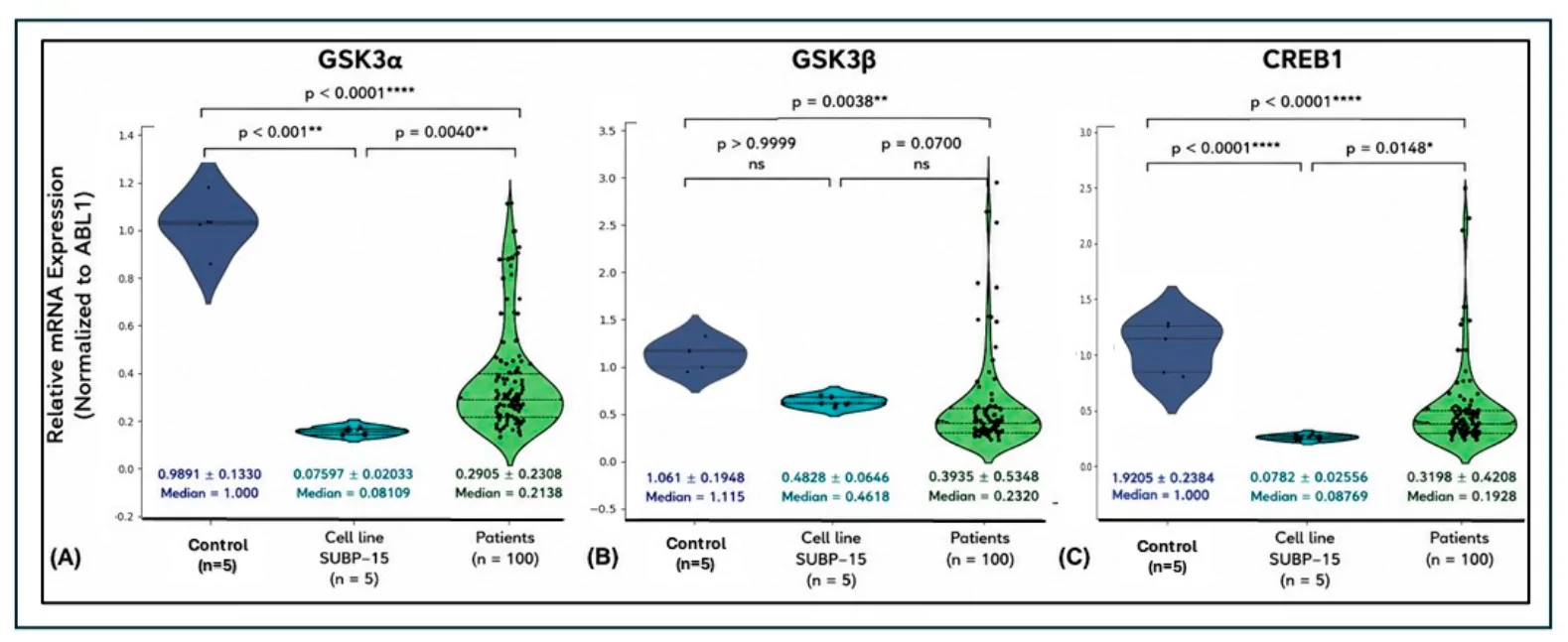
RT-qPCR analysis of GSK3α (**A**), GSK3β (**B**), and CREB1 (**C**) mRNA expression in Philadelphia chromosome-positive B-cell acute lymphoblastic leukemia (Ph+ B-ALL) patients, healthy controls, and the SUBP-15 Ph+ B-ALL cell line. Relative mRNA expression levels were normalized to ABL1. Violin plots show the distribution of individual expression measurements, with individual data points overlaid; dashed lines indicate the median and interquartile range. Mean ± SD and median values are reported for each group below the corresponding violin plots. Statistical comparisons were performed using the Kruskal–Wallis test followed by Dunn’s multiple-comparisons test with Bonferroni correction; all reported pairwise *p*-values are Bonferroni-adjusted. Patient expression data were non-normally distributed according to the Shapiro–Wilk test for GSK3α (W = 0.7832, *p* < 0.0001), GSK3β (W = 0.6097, *p* < 0.0001), and CREB1 (W = 0.5599, *p* < 0.0001). (**A**) **GSK3α:** Expression differed significantly among the three groups (Kruskal–Wallis H(2) = 23.34, *p* < 0.0001). Mean ± SD/median expression was 0.9891 ± 0.1330/1.000 in controls, 0.07597 ± 0.02033/0.08109 in SUBP-15 cells, and 0.2905 ± 0.2308/0.2138 in patients. Dunn’s post hoc analysis showed significantly lower expression in SUBP-15 cells versus controls (adjusted *p* < 0.001), patients versus controls (adjusted *p* < 0.0001), and SUBP-15 cells versus patients (adjusted *p* = 0.0040). Patient expression was approximately 71% lower and SUBP-15 expression approximately 92% lower than controls. (**B**) **GSK3β:** Expression differed significantly among the three groups (Kruskal–Wallis H(2) = 14.88, *p* = 0.0006). Mean ± SD/median expression was 1.061 ± 0.1948/1.115 in controls, 0.4828 ± 0.0646/0.4618 in SUBP-15 cells, and 0.3935 ± 0.5348/0.2320 in patients. Dunn’s post hoc analysis showed significantly lower expression in patients versus controls (adjusted *p* = 0.0038), whereas the comparisons between SUBP-15 cells and controls (adjusted *p* > 0.9999) and between SUBP-15 cells and patients (adjusted *p* = 0.0700) were not significant. Patient expression was approximately 63% lower and SUBP-15 expression approximately 55% lower than controls; the latter reduction was not statistically significant. (**C**) **CREB1:** Expression differed significantly among the three groups (Kruskal–Wallis H(2) = 18.56, *p* < 0.0001). Mean ± SD/median expression was 0.9205 ± 0.2384/1.000 in controls, 0.0782 ± 0.02556/0.08769 in SUBP-15 cells, and 0.3198 ± 0.4208/0.1928 in patients. Dunn’s post hoc analysis showed significantly lower expression in SUBP-15 cells versus controls (adjusted *p* < 0.0001), patients versus controls (adjusted *p* = 0.0053), and SUBP-15 cells versus patients (adjusted *p* = 0.0148). Patient expression was approximately 65% lower and SUBP-15 expression approximately 92% lower than controls. ns, not significant; * *p* < 0.05; ** *p* < 0.01; **** *p* < 0.0001. Patient samples, *n* = 100; healthy controls, *n* = 5 (BR = 5;TR = 3/BP); SUBP-15 cell line, *n* = 5.

To validate the transcriptional expression of CREB1 and GSK3α/β in Philadelphia chromosome-positive B-cell acute lymphoblastic leukemia (Ph+ B-ALL), quantitative RT-PCR analysis was performed in 100 newly diagnosed Ph+ B-ALL patients, 15 healthy controls (5*3), and the SUBP-15 Ph+ B-ALL cell line. RNA was extracted from peripheral blood mononuclear cells (patients and controls) or cultured SUBP-15 cells, and relative mRNA expression levels were normalized to ABL1 (Figure 4, Supplementary Figure S3, Tables S7 and S8). Prior to statistical analysis, data normality was evaluated using the Shapiro–Wilk test. Patient data significantly deviated from normality for all three target genes (GSK3α: W = 0.7832, *p* < 0.0001; GSK3β: W = 0.6097, *p* < 0.0001; CREB1: W = 0.5599, *p* < 0.0001), whereas the control and cell line groups (*n* = 5 each) passed normality testing. Accordingly, non-parametric Kruskal–Wallis tests followed by Dunn’s post hoc multiple comparisons test (Bonferroni correction) were applied for all between-group analyses. All *p*-values reported for pairwise comparisons are Bonferroni-adjusted values from Dunn’s test. GSK3α expression differed significantly across the three groups (Kruskal–Wallis H(2) = 23.34, *p* < 0.0001). GSK3α mRNA expression was significantly downregulated in patient samples (0.2905 ± 0.2308, mean ± SD; median = 0.2138) compared with healthy controls (0.9891 ± 0.1330; median = 1.000; Dunn’s post hoc, adjusted *p* < 0.0001), representing an approximately 71% reduction. Expression of GSK3α was further reduced in the SUBP-15 cell line (0.07597 ± 0.02033; median = 0.08109) compared with controls (0.9891 ± 0.1330; *p* < 0.001), corresponding to an approximately 92% reduction relative to controls. Furthermore, GSK3α expression remained significantly lower in the SUBP-15 cell line than in patient samples (Dunn’s post hoc, adjusted *p* = 0.0040). For GSK3β, the Kruskal–Wallis test revealed significant overall group differences (H(2) = 14.88, *p* = 0.0006). Healthy controls exhibited high expression levels (1.061 ± 0.1948; median = 1.115), whereas both patient samples (0.3935 ± 0.5348; median = 0.2320) and the SUBP-15 cell line (0.4828 ± 0.0646; median = 0.4618) showed reduced expression. Patient samples demonstrated a significant 63% reduction in GSK3β expression compared with controls (Dunn’s post hoc, adjusted *p* = 0.0038). Although the SUBP-15 cell line exhibited an approximately 55% reduction in GSK3β expression relative to controls, this difference did not reach statistical significance after post hoc correction (Dunn’s post hoc, adjusted *p* > 0.9999). No significant difference in GSK3β expression was observed between the SUBP-15 cell line and patient samples (adjusted *p* = 0.0700). CREB1 expression also differed significantly across the three groups (Kruskal–Wallis H(2) = 18.56, *p* < 0.0001). Healthy controls had higher CREB1 expression (0.9205 ± 0.2384; median = 1.000), whereas patient samples exhibited significantly lower expression (0.3198 ± 0.4208; median = 0.1928; Dunn’s post hoc, adjusted *p* = 0.0053), representing an approximately 65% reduction compared with controls. The SUBP-15 cell line demonstrated markedly reduced CREB1 expression (0.0782 ± 0.02556; median = 0.08769; Dunn’s post hoc, adjusted *p* < 0.0001), corresponding to an approximately 92% reduction relative to controls. In addition, CREB1 expression remained significantly higher in patient samples than in the SUBP-15 cell line (Dunn’s post hoc, adjusted *p* = 0.0148).

Overall, GSK3α and CREB1 were significantly downregulated in both Ph+ B-ALL patient samples and the SUBP-15 cell line relative to healthy controls, with the greatest magnitude of reduction consistently observed in the cell line. For GSK3β, significant downregulation was confirmed in patient samples compared with controls; however, the reduction observed in the SUBP-15 cell line, while numerically substantial (~55%), did not achieve statistical significance by Dunn’s post hoc analysis. Notably, the observed reduction in mRNA expression contrasts with the increased phosphorylation levels described in the previous section, suggesting that post-transcriptional or post-translational regulatory mechanisms might be playing a role in functional activation of these signaling molecules in Ph+ B-ALL.

### 2.4. Western Blot Validation for GSK3α/β and CREB1 in 50 Patient Samples

**Figure 5:**
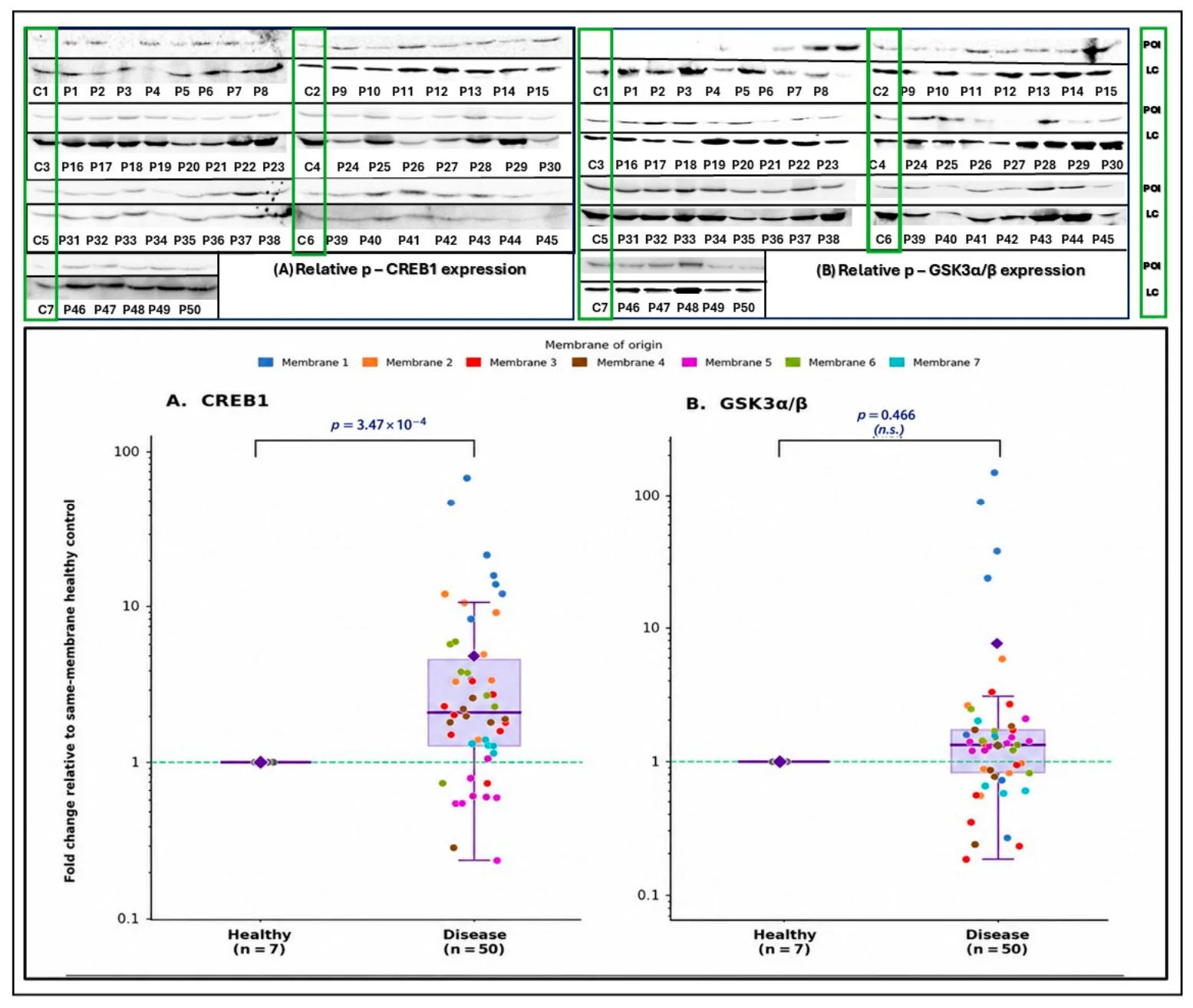
Quantitative Western blot analysis of CREB1 and GSK3α/β protein expression in Ph+ B-ALL patient samples. Background-adjusted protein-of-interest band intensity was normalized to the corresponding beta-actin band intensity. Fold change was calculated relative to the healthy control on the same membrane (healthy reference = 1.00). Each point represents one individual sample; box = IQR, horizontal line = median, diamond = geometric mean, whiskers = range. A logarithmic y-axis is used to display the right-skewed distributions without excluding high-expression observations; **POI = protein of interest, LC = Loading control (beta-actin).** (**A**) 50 patients and 7 healthy controls; median 2.15× (IQR 1.46–4.15), mean 4.58×, SD 7.19, geometric mean 2.54×, range 0.39–41.37×. Membrane-adjusted effect = 2.49× (95% CI 1.55–4.01), *p* = 3.47 × 10^−4^. (**B**) 50 patients and 7 healthy controls; median 1.25× (IQR 0.80–1.58), mean 7.02×, SD 25.15, geometric mean 1.42×, range 0.20–157.75×. Membrane-adjusted effect = 1.38× (95% CI 0.57–3.35), *p* = 0.466 (n.s.) (Supplementary Table S13). **Primary inferential analysis:** ordinary least-squares linear model of log_2_(POI/beta-actin) with disease group as the biological factor and membrane identity as a technical blocking factor. Reported fold effect and 95% CI were backtransformed from the model. All tests two-sided; no observations excluded solely because of extreme magnitude.

Western blot analysis was performed across seven separate membranes, each containing one healthy control lane and a variable number of Ph+ B-ALL patient lanes (total *n* = 7 healthy controls, *n* = 50 patients, 57 observations) (Figure 5; full blot images: Supplementary Figures S4 and S5 and Table S9). Band intensities for p-CREB1 and p-GSK3α/β were normalized to the corresponding beta actin loading-control band intensity from the same lane to yield normalised expression values. Prior to statistical comparison, the normality of normalized expression values was assessed using the Shapiro–Wilk test (Supplementary Table S10). For raw (untransformed) normalized values, both groups departed significantly from normality for p-CREB1 (healthy controls: W = 0.753, *p* = 0.014, *n* = 7; patients: W = 0.787, *p* < 0.0001, *n* = 50). For p-GSK3α/β, the patient group was significantly non-normal (W = 0.843, *p* < 0.0001, *n* = 50); the healthy control group did not reach statistical significance (W = 0.861, *p* = 0.155, *n* = 7), although the limited power of the Shapiro–Wilk test at *n* = 7 means this result cannot be taken as confirmation of normality. Inspection of the raw data revealed pronounced right-skewness in both proteins. Log2 transformation was therefore applied to all normalized values prior to the primary analysis. After transformation, the control-group distributions were no longer significantly non-normal for either protein (p-CREB1: W = 0.910, *p* = 0.392; p-GSK3α/β: W = 0.829, *p* = 0.079). The patient-group distributions remained significantly non-normal after transformation (p-CREB1: W = 0.914, *p* = 0.00139; p-GSK3α/β: W = 0.898, *p* = 0.000419), reflecting residual heterogeneity across the patient cohort. Since the samples were distributed across seven separate Western blot membranes, inter-membrane technical variation was expected to be a major source of variance. A conventional two-sample test applied to marginal values would confound this technical variation with the biological group effect. The primary analysis therefore used a linear model fitted to log2-transformed normalized expression values, with disease group (healthy controls vs. Ph+ B-ALL patients) as the fixed biological factor and Western blot membrane as a technical blocking factor, implemented in R (lm function; R version 4.x). Blocking for membrane removes inter-membrane variation from the residual error term, yielding an unconfounded estimate of the between-group difference.

For p-CREB1, the linear model identified a statistically significant upregulation in Ph+ B-ALL patients relative to healthy controls (β = 1.317, SE = 0.342, t(49) = 3.846, *p* = 0.000347, 95% CI [0.629, 2.005]), corresponding to a 2.49-fold higher expression in patients after adjustment for membrane. The model explained a high proportion of total variance (R^2^ = 0.957, adjusted R^2^ = 0.951), confirming that the membrane blocking factor captured the dominant source of variation in the data.

For p-GSK3α/β, the group effect did not reach statistical significance (β = 0.467, SE = 0.636, t(49) = 0.735, *p* = 0.466, 95% CI [−0.811, 1.745]), corresponding to an estimated 1.38-fold difference that cannot be distinguished from chance. The model R^2^ was lower for this protein (R^2^ = 0.735, adjusted R^2^ = 0.698), reflecting greater within-membrane variability in GSK3α/β expression across the patient cohort.

Residual diagnostics indicated that, despite log2 transformation, the model residuals showed some deviation from normality for both proteins (Shapiro–Wilk: p-CREB1 W = 0.948, *p* = 0.016; p-GSK3α/β W = 0.935, *p* = 0.004). For p-CREB1, the large *t*-statistic (t = 3.85) and strong effect size (2.49-fold) make it unlikely that this modest residual non-normality materially affects the conclusion of significant upregulation. For p-GSK3α/β, the result was already non-significant; the residual departure does not alter this interpretation. A two-sided Mann–Whitney U test was additionally applied to the log2-transformed normalized values, without membrane adjustment, as a non-parametric reference. Neither p-CREB1 (U = 150, *p* = 0.559, rank-biserial r = 0.143) nor p-GSK3α/β (U = 155, *p* = 0.642, rank-biserial r = 0.114) reached significance by this test. The absence of significance in the Mann–Whitney analysis reflects the structure of the data rather than a true null result: because the Mann–Whitney test does not incorporate the membrane blocking factor, the large inter-membrane variation in band intensities acts as an uncontrolled confound, substantially reducing the test’s power to detect the within-membrane group effect. Consistent with this interpretation, the membrane blocking factor alone accounted for 95.7% of total variance in p-CREB1 expression (model R^2^ = 0.957), meaning that a test that ignores membrane structure cannot reliably isolate the biological signal. The linear model with membrane blocking remains the primary and appropriate analysis for this blocked design; the Mann–Whitney results are reported for context only and should not be interpreted as contradicting the primary finding of significant CREB1 upregulation (Supplementary Figures S6 and S7 and Tables S11 and S12).

In summary, p-CREB1 protein expression was significantly elevated in Ph+ B-ALL patients compared with healthy controls (2.49-fold, *p* < 0.001), consistent with a role for CREB1 in the pathobiology of this disease. p-GSK3α/β protein expression did not differ significantly between groups under the same analytical framework (*p* = 0.466). These results were obtained from densitometric analysis of Western blot membranes with beta actin loading-control normalization, using a blocked linear model to account for inter-membrane technical variation.

## 3. Discussion

The study identifies CREB1 and GSK3 isoforms as highly connected nodes in the PI3K/Akt/mTOR signaling network that exhibit paradoxical upregulation following pathway inhibition in acute lymphoblastic leukemia (ALL). The central finding is discordance between transcriptional suppression and phosphoprotein signaling. Despite approximately 63–71% reductions in CREB1, GSK3α and GSK3β mRNA levels in patient samples (GSK3α ~71%, GSK3β ~63%, CREB1 ~65%), phospho-protein levels were maintained or elevated in SUBP-15 cells following inhibitor treatment, with p-CREB(Ser133) showing statistically significant increases following Perifosine (6.295-fold; BH-q = 0.033), GDC-0941 (4.658-fold; BH-q = 0.032), and Rapamycin (5.528-fold; BH-q = 0.042), and p-GSK3α/β(Ser21/9) showing a statistically significant increase following Perifosine (3.123-fold; BH-q = 0.023). In the patient cohort, Western blot analysis similarly demonstrated a significant increase in p-CREB(Ser133) signal (2.49-fold; β = 1.317, *p* < 0.001), whereas GSK3α/β protein expression did not differ significantly between groups (*p* = 0.466). Since the measurements of CREB in both the phospho-kinase array and patient Western blot analysis were of phospho-CREB(Ser133), rather than total CREB, these findings should be interpreted as evidence of altered CREB phosphorylation-associated signaling rather than increased total CREB protein abundance. Furthermore, because total CREB was not measured in parallel, the present data cannot establish whether the proportion of the cellular CREB pool phosphorylated at Ser133 was increased. This mRNA–phosphoprotein discordance suggests that transcriptional abundance and phosphorylation-associated signaling of CREB1 may be regulated independently, though a precise molecular basis for the same requires further investigation. These findings are consistent with emerging evidence that targeted kinase inhibitors trigger adaptive kinome reprogramming, wherein cancer cells activate compensatory signaling pathways to circumvent therapeutic blockade [46,47,48]. It is important to emphasize, however, that these data demonstrate association rather than causation: while we observe correlation between pathway inhibition and CREB/GSK3 phosphorylation changes, the data do not establish whether these kinase activation events are necessary or sufficient for resistance.

Computational analysis from the protein–protein interaction (PPI) network identified CREB1 and GSK3 isoforms as high-centrality nodes connecting multiple upstream regulators such as Akt1, MAPK1/3, STAT3, TP53, NF-κB, and MYC to downstream effectors regulating survival, proliferation, and metabolism [49,50]. While the identification of hub proteins through network analysis is a commonly employed approach and does not by itself establish functional significance, it was further supported by hierarchical clustering of phospho-kinase array data. Following treatment with the PI3K inhibitor (GDC-0941), the Akt inhibitor (Perifosine) and the mTOR inhibitor (Rapamycin), CREB1 (cluster 3) and GSK-3α/β (cluster 2) displayed marked upregulation rather than expected suppression. Notably, p-CREB(Ser133) was significantly elevated by all three agents (BH-q < 0.05 in each case), whereas the elevation of p-GSK3α/β(Ser21/9) reached statistical significance only following Perifosine treatment (BH-q = 0.023), with the increases observed after GDC-0941 (BH-q = 0.287) and Rapamycin not reaching statistical significance. Since the array signal represents phospho-CREB rather than total CREB, the consistent increase in p-CREB across these treatments indicates a reproducible change in CREB phosphorylation-associated signaling, but cannot by itself distinguish increased phosphorylation of a larger CREB pool from increased phosphorylation stoichiometry within an unchanged or reduced pool. This paradoxical response suggests that these proteins might occupy strategic positions within the signaling network, enabling the integration of inputs from multiple pathways and potentially facilitating adaptive responses to targeted inhibition [50]. The concept of adaptive kinase reprogramming has been extensively documented across cancer types: Schwill et al. demonstrated that HER2-targeted therapy in breast cancer triggers a common adaptive response program involving activation of alternative receptor tyrosine kinases and downstream signaling nodes [51], and phosphoproteomics studies in acute myeloid leukemia have similarly revealed that kinase inhibitor resistance is mediated by rapid rewiring of phospho-signaling networks with compensatory activation of parallel pathways [46,52,53]. Our findings extend this paradigm to Ph+ B-ALL, suggesting that CREB1 and GSK3α/β may function as convergence points for multiple compensatory pathways activated upon PI3K/Akt/mTOR inhibition.

The persistence of CREB phosphorylation at Ser133 despite PI3K/Akt/mTOR inhibition was contrary to the expected canonical signaling models. Treatment with Perifosine, GDC-0941, and Rapamycin consistently resulted in significant upregulation of p-CREB, indicating that CREB activation might be sustained through alternative signaling. This interpretation should, however, be considered in the context of the phospho-specific nature of the assay. The observed increase in p-CREB (Ser133) provides evidence for increased phosphorylation-associated signaling at this site, but without paired total CREB measurements it cannot establish that the proportion of CREB present in the phosphorylated state was increased. Moreover, p-CREB alone does not establish downstream transcriptional activity, which also depends on factors such as CBP/p300 or CRTC availability, promoter context, and other regulatory modifications. Accumulating evidence implicates CREB in adaptive resistance to therapeutic modalities in hematologic malignancies: Masic et al. demonstrated that hyperactive CREB subpopulations increase during therapy in pediatric B-lineage ALL, with phospho-CREB-high cells exhibiting enhanced survival under therapeutic stress [54], directly paralleling our observation of elevated p-CREB (Ser133) in SUBP-15 cells following PI3K/Akt/mTOR inhibitor treatment, and elevated total p-CREB1 protein in Ph+ B-ALL patient samples. In AML, Wang et al. showed that downregulation of CSRP2 promotes proliferation through upregulation of p-AKT and p-CREB, with CREB activation contributing to chemoresistance [55], while Lazaro-Navarro et al. recently reported that inhibiting H3K27 demethylases downregulates CREB–CREBBP signaling, overcoming resistance in relapsed ALL [56]. This suggests that CREB pathway activation is a functionally relevant resistance mechanism, though our study does not yet provide direct functional evidence for this role in Ph+ B-ALL. Mechanistically, CREB promotes resistance through transcriptional upregulation of anti-apoptotic genes (BCL2, MCL1), metabolic reprogramming genes (glycolytic enzymes), and drug efflux transporters (ABCB1/MDR1) [57,58]; whether CREB mediates similar transcriptional programs through PI3K/Akt/mTOR inhibition in Ph+ B-ALL holds potential for investigation. Several kinase pathways have been reported to phosphorylate CREB at Ser133 independently of PI3K/Akt signaling, including MAPK/ERK-activated kinases (RSK and MSK) downstream of PDGF or EGFR signaling [59,60]. Hayun et al. demonstrated that ERK activity in immature leukemic cells drives clonal selection during AML induction therapy [61], and Gab2 has been shown to promote AML growth and migration through the SHP2-Erk-CREB signaling pathway [62], providing direct mechanistic links between RTK signaling and CREB activation. Additionally, the cAMP/PKA pathway, the canonical upstream CREB Ser133 kinase, may also contribute. Ma et al. recently demonstrated that quercetin inhibits the cAMP/PKA/CREB/glycolysis axis to exert anti-ALL effects, confirming that this pathway is active in ALL [63], and Yamagishi et al. showed that sorcin-mediated multidrug resistance in leukemia cells operates via CREB through MDR1 upregulation [57]. Multiple RTKs can further activate CREB through both MAPK/ERK and PI3K/Akt pathways; Nasimian et al. identified AXL receptor tyrosine kinase dependency in leukemia, suggesting RTK activation as a potential adaptive resistance mechanism [64]**.** Calcium-dependent kinases (CaMKII and CaMKIV) involved in hematopoietic cell activation and stress-responsive kinases such as p38 MAPK and JNK [65,66] also represent alternative routes. Sun et al. demonstrated that DUSP1-mediated MAPK pathway modulation affects cytarabine sensitivity in AML [67,68], and Huang et al. showed that CaMKII–CREB1 signaling contributes to drug resistance through regulation of ABCB1 expression [69]. However, these alternative pathways were not directly evaluated in this study and their functional contribution to CREB phosphorylation under these experimental conditions remains to be tested.

The functional consequences of CREB phosphorylation at Ser133 are directly relevant to the resistance phenotype suggested by the present data [70,71]. Mechanistically, Ser133 phosphorylation is the canonical activating modification for CREB-dependent transcription, recruiting the coactivator CBP/CREBBP and its paralogue p300 via their KIX domains to enable RNA Pol II-dependent transcription of CRE-containing target genes [72]. Several CREB transcriptional programs have documented roles in cancer drug resistance: (i) p-CREB (Ser133) drives transcription of BCL2, MCL1, and BCL-XL, directly counteracting the apoptotic pressure imposed by PI3K/Akt/mTOR inhibitors [70]; (ii) CREB transcriptionally activates MDR1 (ABCB1, encoding P-glycoprotein), thereby reducing intracellular drug accumulation [73]; (iii) CREB regulates cyclin gene expression, supporting G1/S transition and sustaining proliferative capacity under therapeutic stress [74]; (iv) CREB/CBP activity supports transcription of metabolic enzymes involved in oxidative phosphorylation and glycolysis, maintaining bioenergetic homeostasis [75]; and (v) Ser133 phosphorylation itself enhances CREB protein stability by reducing proteasomal degradation [76,77]. Critically, single-cell mass cytometry data in pediatric B-ALL demonstrated that p-CREB-high subpopulations are specifically enriched during induction therapy, and pharmacological CREB inhibition (compound 666-15) induced apoptosis in patient-derived ALL cells, providing direct functional evidence that p-CREB marks therapy-tolerant ALL cells [54]. These functional consequences are highly congruent with the adaptive signaling context observed in this study. However, whether these transcriptional programs are specifically activated in PI3K/Akt/mTOR-inhibited Ph+ B-ALL requires direct functional validation using transcriptional reporter assays or transcriptomic profiling of inhibitor-treated cells, which was beyond the scope of this study.

In contrast to the other inhibitors tested, GSK690693 (a pan-Akt inhibitor) did not produce a statistically significant change in p-CREB(Ser133) levels (BH-q = 0.070), making it the only agent among those tested that failed to significantly alter CREB phosphorylation. This differential response is noteworthy: whereas Perifosine, GDC-0941, and Rapamycin all significantly elevated p-CREB, the pan-Akt inhibitor GSK690693 did not, despite also targeting the Akt node. One possible explanation is that the structural and mechanistic differences between these Akt-targeting agents translate into distinct downstream phospho-signaling profiles; alternatively, Akt-independent compensatory mechanisms activated by the other three agents may not be equivalently engaged following GSK690693 treatment. This differential response profile raises the possibility that combinatorial therapeutic approaches targeting the PI3K/Akt/mTOR axis together with MEK/ERK, CaMK, or p38 MAPK signaling may help modulate compensatory CREB activation in a context-dependent manner.

GSK3 exhibits context-dependent functions in leukemia, with both its active (dephosphorylated) and inactive (phosphorylated at Ser21/Ser9) forms contributing to disease progression via distinct known mechanisms [78]. Phosphorylation at Ser21 (GSK-3α) and Ser9 (GSK-3β) inhibits kinase activity. In our kinase array data, Perifosine produced a statistically significant elevation of p-GSK-3α/β(Ser21/9) (3.123-fold; BH-q = 0.023), while the increase observed with GDC-0941 did not reach statistical significance (1.607-fold; BH-q = 0.287). Notably, analysis of the discrete GSK-3β(Ser9) entry revealed a 2.877-fold increase with Perifosine (BH-q = 0.023) and, in contrast, a significant reduction to undetectable signal following Rapamycin treatment (BH-q = 0.042), highlighting the divergent effects of individual inhibitors on GSK3β phosphorylation. The significant elevation of p-GSK-3α/β(Ser21/9) following Perifosine treatment aligns with reports linking GSK3 phosphorylation to leukemia progression through PI3K/Akt signaling in acute myeloid leukemia [79,80]. In chronic myeloid leukemia, Martelli et al. reviewed evidence that GSK3 activity is required for leukemia stem cell maintenance and that GSK3 inhibition can enhance TKI efficacy [80,81]. However, Liang et al. demonstrated that GSK3 promotes TKI-induced apoptosis in Ph+ leukemia cells by regulating the Wnt/β-catenin pathway [82,83], illustrating the highly context-dependent nature of GSK3’s role. The observation that Perifosine was the only agent to produce a statistically significant elevation of p-GSK3α/β(Ser21/9) is consistent with studies showing that Akt-mediated GSK3 phosphorylation at Ser21/9 promotes cancer cell survival by preventing GSK3-mediated degradation of MCL1 and MYC [80,84], though the functional consequences of this phosphorylation change in Ph+ B-ALL remain to be experimentally validated. The therapeutic relevance of GSK3 modulation has been further highlighted in AML: Zhou et al. demonstrated that GLI1 reduces drug sensitivity by regulating the cell cycle through the PI3K/AKT/GSK3/CDK pathway [67], and Lee et al. showed that Pim kinase inhibitors increase gilteritinib cytotoxicity in FLT3-ITD AML through GSK3β activation and subsequent c-Myc and Mcl-1 proteasomal degradation [84], suggesting that restoring GSK3 activity may enhance drug sensitivity in certain leukemia contexts.

When GSK3 is inactivated by phosphorylation, pro-survival pathways including PI3K/Akt/mTOR, NF-κB and Wnt/β-catenin are activated, thereby promoting accumulation of anti-apoptotic proteins such as survivin and XIAP and conferring chemotherapy resistance [85,86,87,88] Conversely, active (unphosphorylated) GSK3 can also enhance NF-κB signaling to support leukemic proliferation [80,89], or mediate pro-apoptotic effects during glucocorticoid-induced or Akt-inhibited apoptosis by phosphorylating Bim and facilitating caspase activation [90,91,92]. Thus, GSK3 isoforms may function as molecular switches whose biological effect is highly dependent on pathway crosstalk and cellular context [93,94]. Although clear expression trends were observed, the absence of a statistically significant difference in p-GSK-3α/β levels across the 50-patient (*p* = 0.466) cohort might reflect the substantial biological heterogeneity characteristic of leukemia [95,96,97]. Furthermore, regulatory feedback mechanisms that operate within interconnected signaling networks might also limit GSK3 activity to prevent excessive pro-apoptotic signaling, thereby contributing to variability observed. The persistence of inhibitory phosphorylation despite suppression of the Akt pathway can also be explained by the activation of alternative Akt-independent kinases like PKA, PKC, and p90RSK, all of which are capable of and might be phosphorylating and inhibiting GSK3 [98,99,100].

The interpretation of increased p-GSK3alpha/beta (Ser21/9) following Perifosine treatment requires careful consideration. Phosphorylation at Ser21 (GSK3alpha) and Ser9 (GSK3beta) is an inhibitory modification that occludes the substrate-binding groove of the kinase; therefore, the observed increase in p-GSK3alpha/beta (Ser21/9) represents GSK3 inactivation with the following cellular functional consequences [78]: (i) active (unphosphorylated) GSK3 phosphorylates oncoproteins including c-Myc (Thr58), cyclin D1 (Thr286), and MCL-1 (Thr163) for proteasomal degradation, such that Ser21/9-mediated inactivation stabilizes these proteins and promotes cell proliferation and resistance to apoptosis [78,101]; (ii) inactive GSK3 cannot phosphorylate beta-catenin in the destruction complex, leading to beta-catenin nuclear accumulation and Wnt target gene transcription that maintains leukemia stem cell self-renewal and has been linked to TKI resistance in BCR-ABL1-positive leukemia [98]; (iii) GSK3 activity is specifically required for rapalog-induced cyclin D1 degradation, such that inhibitory Ser21/9 phosphorylation can blunt the cell cycle-arresting effects of mTOR inhibitors—directly relevant to the Rapamycin treatment context of this study [102,103]; and (iv) in AML patients, high p-GSK3alpha/beta (Ser21/9) correlated with AKT activation and worse overall survival, providing clinical evidence that inhibitory GSK3 phosphorylation is associated with poor outcomes in acute leukemia [104]. The functional duality of GSK3 should be acknowledged: while Ser21/9-mediated inactivation generally promotes pro-survival signaling via the above mechanisms, active GSK3 can also support leukemic proliferation in some contexts through NF-kappaB activation and maintenance of leukemia stem cells [87,93], which may contribute to the heterogeneous p-GSK3 levels observed across the 50 patient Western blot samples (*p* = 0.466) and underscores the need for patient stratification and subgroup analysis in future studies [81].

A notable finding was the marked discordance between reduced mRNA expression (approximately 63–71% downregulation: GSK3α ~71%, GSK3β ~63%, CREB1 ~65%) and the maintenance or upregulation of corresponding phosphorylation signals. In particular, CREB1 transcript abundance was reduced in patient samples, whereas p-CREB(Ser133) was significantly elevated in the patient Western blot analysis and following treatment with Perifosine, GDC-0941, and Rapamycin in SUBP-15 cells. This discrepancy suggests that transcriptional abundance and phosphorylation-associated signaling may be regulated independently; however, because total CREB protein was not measured, the present data cannot determine whether the increased p-CREB signal reflects greater phosphorylation of an unchanged or altered total CREB pool. This mRNA–phosphoprotein decoupling phenomenon is increasingly recognized as a mechanism of drug resistance across cancer contexts; Bica et al. demonstrated through phosphoproteomics that BCR::ABL1-independent resistance in CML involves post-transcriptional regulatory mechanisms that maintain kinase activity despite transcriptional changes [52]. Several non-mutual exclusive explanations might account for the observation; however, these were not directly investigated in this study. One possibility is that post-transcriptional regulatory processes permit continued protein synthesis despite reduced transcript abundance. For example, under conditions of cellular stress, cap-dependent translation is frequently suppressed, where selected mRNAs continue to be translated through internal ribosome entry sites (IRES) [105]. In addition to this, IRES trans-acting factors (ITAFs) and RNA-binding proteins such as IGF2BP1 are known to stabilize oncogenic mRNAs in B-ALL [106,107,108,109,110,111,112,113]. Additionally, mRNA translation efficiency for CREB1 and GSK3 isoform transcripts may be enhanced through mTOR-independent translation initiation mechanisms, and it cannot be excluded that CREB1 and GSK3 genes utilize alternative promoters or transcription start sites not captured by our RT-PCR primers but nonetheless producing functional protein. Whether CREB1 and GSK3 isoform transcripts are subject to similar selective translational regulatory mechanisms remains unknown and warrants further investigation using approaches such as RNA immunoprecipitation, ribosome profiling, or polysome fractionation.

Post-translational stabilization may be another potential explanation. Reduced ubiquitination or increased chaperone activity also prolongs protein half-life and sustains protein abundance despite diminished transcript levels [114]. Illiano et al. demonstrated that CREB stability is regulated by a PKA- and proteasome-dependent mechanism [77], suggesting that reduced proteasomal degradation could maintain CREB protein levels despite suppressed mRNA and representing a potential pharmacological vulnerability. In T-ALL, HSF1-mediated chaperone pathways have been known to maintain mTORC1 signaling and stabilize key regulatory proteins [115]. Whether analogous mechanism contribute to the stabilization of GSK3α/β and CREB in B-ALL again needs experimental validation [90,116]. Protein stability and function might be further influenced by phosphorylation itself; CREB’s phosphorylation at Ser133 residue is known to enhance both transcriptional activity and protein persistence [72,76,117], whereas phosphorylation of GSK3 regulates degradation of multiple downstream targets like c-Myb and members of the MAF family [118,119]. In hematologic malignancies, dysregulated GSK3 activity is linked to altered stability of oncogenic proteins such as β-catenin, c-Myc, and NOTCH1, thereby contributing to disease progression [78,81,87,97]. In aberrant PI3K/Akt/mTOR signaling, impaired ubiquitination or enhanced chaperone-mediated protection may delay protein degradation [102,120,121,122]; this has been reported in B-ALL cases characterized by PTEN loss and proteasome dysfunction [123]. MicroRNA-mediated regulation of CREB1 and GSK3 in hematologic malignancies has been studied [124,125,126,127,128,129,130,131]. However, the role of miRNAs in sustaining protein levels despite reduced transcripts in ALL has not been evaluated and remains an interesting area for future investigation [132,133].

Our findings are consistent with a model where leukemia cells adapt to PI3K/Akt/mTOR inhibition via dynamic remodeling of signaling networks. In this framework, inhibition of pathway initially attenuates canonical survival signaling, resulting in reduced proliferation and high apoptotic stress. Concurrently, CREB1 and GSK3 expressions also undergo transcriptional downregulation as part of a feedback regulatory response. However, the reduction in transcript abundance does not lead to functional inactivation of the corresponding proteins. Instead, leukemic cells might compensate through activation of alternative kinase pathways such as MAPK/ERK, CaMK and stress-responsive signaling cascades, thereby contributing to phosphorylation at regulatory sites such as Ser133 on CREB and Ser21/9 on GSK3. This study did not directly measure these upstream kinases, however; this therefore represents a mechanistic hypothesis rather than an established pathway in the current experimental system. Mechanistically, this adaptive response is consistent with the concept of feedback relief and kinome reprogramming [47,48,51]. PI3K/Akt/mTOR signaling normally exerts negative feedback on upstream RTKs and parallel pathways; upon inhibition, this suppression is relieved, leading to RTK upregulation [51,64], MAPK/ERK pathway activation [61,62], cAMP/PKA pathway activation [63,134] and stress kinase activation [67], all converging on CREB and GSK3α/β to sustain their phosphorylation despite upstream inhibition. This model is supported by phosphoproteomics studies demonstrating rapid kinome reprogramming following targeted inhibitor treatment [46,48,52,53,135]. Although the adaptive mechanism provides a plausible explanation for the observed findings, direct experimental studies are further required to determine whether the pathways functionally bypass PI3K/Akt/mTOR inhibition in this context. Sustained CREB phosphorylation may be contributing to prolonged protein stability and continued transcriptional activity. Nevertheless, the extent to which the same effects influence therapeutic resistance remains to be established experimentally. The consistent and significant elevation of p-CREB(Ser133) across three of the four inhibitors tested (BH-q = 0.033, 0.032, and 0.042 for Perifosine, GDC-0941, and Rapamycin, respectively), together with the 2.49-fold elevation of p-CREB(Ser133) observed by Western blot analysis in Ph+ B-ALL patient samples (β = 1.317, *p* < 0.001), are notable observations. However, future studies incorporating paired measurements of total CREB and p-CREB in the same samples will be important to determine whether the observed increase in p-CREB reflects increased phosphorylation relative to the available CREB pool. Also, independent validation using orthogonal approaches such as immunofluorescence microscopy, flow cytometry-based phospho-flow analysis, or alternative phospho-specific antibody clones would further strengthen these conclusions.

Future mechanistic and translational studies may benefit from the use of phospho-specific antibody-based approaches together with functional assays such as CRE-luciferase reporter systems to seek to clearly define the biological and clinical significance of the alterations within the signaling cascades. CREB and GSK3, as highly connected network nodes with altered phosphorylation patterns, are of interest as potential candidates for combination therapy studies. However, whether they play a direct causal role in adaptive resistance requires functional validation through either knockdown, overexpression or rescue experiments, which is a key limitation of this correlative study. Pharmacological co-inhibition studies combining PI3K/Akt/mTOR inhibitors with CREB pathway inhibitors (e.g., KDM inhibitors [56,77]) or GSK3 modulators such as Pim kinase inhibitors [84] represent promising future directions, as does upstream kinase identification through kinase inhibitor panel screening or phosphoproteomics-based kinase activity profiling to definitively identify the specific pathways mediating compensatory CREB/GSK3 activation in Ph+ B-ALL.

The discordance between mRNA expression and phosphoprotein measurements highlights a major limitation of relying on transcriptomic data alone to infer functional signaling states. An integrated approach incorporating both proteomic and phosphoproteomic profiling might be required to accurately reflect the pathway activation state. Whether this decoupling represents a therapeutic vulnerability, for example, by targeting translational regulation (eIF4E/eIF4A) or proteostasis (HSF1, ubiquitin–proteasome system), is an intriguing but an untested hypothesis that merits future investigation. The observation points to a functional framework for both monitoring and treatment, where phosphoprotein dynamics and multi-kinase targeting may help address resistance and improve outcomes in ALL. Adaptive signaling involving the MAPK/ERK pathway may also contribute to sustained CREB phosphorylation under PI3K/Akt/mTOR inhibition [71,136,137]; however, whether this is a functionally important bypass mechanism in Ph+ B-ALL requires direct experimental validation.

When considered collectively, the multi-platform evidence from this study provides a convergent but explicitly correlative association between altered CREB and GSK3 phosphorylation and the Ph+ B-ALL disease and treatment context. In the pharmacological challenge model (phospho-kinase array), p-CREB (Ser133) was the only target to achieve statistical significance across three mechanistically distinct inhibitors, suggesting a generalizable adaptive response to PI3K/Akt/mTOR inhibition. In the clinical arm, p-CREB (Ser133) was significantly elevated 2.49-fold in treatment-naive Ph+ B-ALL patients relative to healthy controls (*p* < 0.001), establishing a disease-context association. The concordance between the pharmacological challenge model and the patient data supports the hypothesis that elevated p-CREB represents an intrinsic feature of Ph+ B-ALL signaling that may be further amplified under therapeutic pressure. However, this study does not provide direct evidence comparing phosphorylation levels between drug-sensitive and drug-resistant states, does not include longitudinal pre- and post-resistance patient samples, and does not establish through functional experiments whether p-CREB or p-GSK3 changes are necessary or sufficient for drug resistance. The evidence is therefore classified as associative rather than causal, and the terms “adaptive signaling node” and “candidate resistance marker” used throughout this manuscript reflect this classification.

Several limitations in this study warrant careful consideration when interpreting the findings. A primary constraint is the reliance on peripheral blood mononuclear cells (PBMCs) for RT-qPCR and proteomic analyses. PBMCs represent a heterogeneous cell population comprising both leukemic blasts and non-malignant immune cells, which can potentially dilute leukemia-specific transcriptional and protein signals. Consequently, the expression profiles obtained may not exclusively reflect the transcriptional activity of malignant cells, contributing to the variability observed across patient-derived samples. In contrast, leukemia cell lines provide a homogeneous malignant population and a more controlled experimental platform, which may partly explain the greater downregulation of GSK3α and CREB1 observed in cell lines compared to patient samples. Thus, discrepancies between these two models should be interpreted with caution, as they likely reflect inherent differences in cellular composition and inter-patient biological heterogeneity.

The inclusion of patient-derived cells as a baseline for in vitro onco-proteomic experiments would have provided a more clinically relevant comparison; however, this was precluded by the limited availability of patient material and experimental resources. While pooled patient samples could mask inter-individual variation, analyzing individual samples would necessitate a substantially larger cohort to adequately characterize patient-to-patient variability. Future studies should, therefore, validate these findings in larger, appropriately designed patient-derived cohorts.

Furthermore, the assessment of steady-state mRNA levels via RT-PCR provides a snapshot of transcript abundance but does not offer comprehensive information regarding transcriptional rates, mRNA stability, or transcript degradation. Techniques such as ribosome profiling or polysome fractionation would provide a more nuanced understanding of the translational regulation of CREB1 and GSK3 isoforms. Similarly, RNA immunoprecipitation could help identify RNA-binding proteins that influence transcript stability. Future research is also required to clarify the post-translational regulatory mechanisms discussed herein. Insights into protein turnover could be gained through protein half-life measurements using pulse-chase assays or ubiquitination profiling. Additionally, evaluating protein distribution across cellular and extracellular compartments—including plasma and extracellular vesicles—would help establish the origin and localization of phosphorylated CREB and GSK3 isoforms in B-ALL.

The study design also presents certain limitations. While the sample size (*n* = 100 for RT-PCR, *n* = 50 for Western blotting) was determined according to STROBE guidelines, the cross-sectional nature of the study remains a restriction. The absence of longitudinal sampling across different disease stages or treatment time points prevents us from determining whether CREB1 and GSK3α/β expression varies according to treatment exposure, response, or relapse. Longitudinal analysis would provide a dynamic representation of phosphorylation states and help clarify the association between p-CREB/p-GSK-3α/β and adaptive resistance.

Regarding technical robustness, Western blot findings should be viewed in light of the normalization strategy. Signals were normalized to β-actin following Bradford quantification; however, post-transfer total-protein normalization (e.g., Ponceau S or stain-free imaging) was not performed. Future studies incorporating validated total-protein normalization alongside β-actin assessment would enhance the quantitative precision of these analyses. Additionally, the upstream signaling events sustaining phosphorylation despite reduced transcripts remain unclear. Future investigations utilizing kinase inhibitor screens and CRISPR-mediated knockdowns could identify the pathways maintaining CREB and GSK3 activation.

Finally, the generalizability of these results is limited by the use of a single Ph+ B-ALL cell line (SUPB-15) and a single institutional patient cohort. The lack of functional perturbation experiments, such as siRNA/CRISPR knockdown or rescue assays, means a direct causal role for CREB1 and GSK3α/β in adaptive resistance has not yet been established. Consequently, these proteins should be considered candidate adaptive response markers rather than confirmed mediators or validated biomarkers. In vivo validation using patient-derived xenograft (PDX) models would additionally be required to assess the clinical relevance of CREB/GSK3 targeting in combination with PI3K/Akt/mTOR inhibitors, given that preclinical studies combining PI3K/Akt/mTOR pathway blockade with MEK or RTK inhibitors have shown promise [47,136].

The broader biological and clinical implications of p-CREB’s upregulation and altered p-GSK-3α/β expression remain to be clarified through studies incorporating clinical outcome data before their utility as biomarkers can be assessed. Integrative approaches such as combining CREB ChIP-seq with transcriptomic and metabolomic analyses might help to elucidate downstream regulatory pathways supporting leukemic cell survival and adaptation. Functional studies investigating the effects of sustained CREB1 and GSK3 activity on cell viability, therapeutic response and disease progression are key to establish causality. Although, while the present findings suggest potential involvement in adaptive resistance, whether the factors play a causal role remains uncertain and requires functional validation. Our identification of CREB1 and GSK3α/β as candidate adaptive signaling nodes suggests several potential combination therapy strategies. Combining PI3K/Akt/mTOR inhibitors with CREB pathway inhibitors, including KDM inhibitors such as GSKJ4, which downregulate CREB through PKA-dependent mechanisms [77], or CREBBP inhibitors (A485, CCS1477), which have shown efficacy in overcoming venetoclax/azacitidine resistance in AML [138], may help prevent adaptive resistance. For GSK3, Pim kinase inhibitors that activate GSK3 to facilitate proteasomal degradation of c-Myc and Mcl-1 may enhance drug sensitivity in appropriate leukemia contexts [84], and targeting upstream adaptive kinases through MEK or RTK inhibitors in combination with PI3K/Akt/mTOR inhibitors merits preclinical investigation [47,139]. Ultimately, the findings should be validated in more physiologically relevant systems like patient-derived xenograft or organoid models and should be further explored in biomarker-driven clinical studies. Such efforts are necessary to determine whether p-CREB and p-GSK-3α/β have utility as candidate biomarkers or therapeutic targets and may contribute to the development of more precise strategies for overcoming adaptive resistance in ALL.

## 4. Materials and Methods

### 4.1. Study Design and Overview

This study employed a comprehensive dual methodological approach to investigate phosphokinase signaling with respect to PI3K/Akt/mTOR pathway inhibition in Philadelphia chromosome-positive (Ph+) B-cell Acute Lymphoblastic Leukemia (B-ALL). The methodology integrated in silico network pharmacology analysis with phosphokinase array profiling to systematically characterize molecular targets across 39 kinase phosphorylation targets. The experimental design was specifically structured to identify and validate key molecular targets, including CREB1, GSK3α, and GSK3β. Additionally, the methodology incorporated comparative mRNA gene expression analysis (RT-qPCR) and phosphoprotein analysis (Western blot) in clinical samples to elucidate the molecular basis of treatment resistance in Ph+ B-ALL. It is important to note that RT-qPCR measures transcript abundance (mRNA expression), whereas the phospho-kinase array and Western blot measure phosphorylation of specific protein residues; these two levels of analysis are not directly equivalent and are interpreted separately throughout this study.

### 4.2. In Silico Network Pharmacology Framework

#### 4.2.1. Array-Based Target Selection and Identification

Target proteins for network pharmacology analysis were selected based on a comprehensive phosphokinase onco-proteome array kit, with detailed information about the selected targets provided in Supplementary Table S14. The selection criteria prioritized proteins with established roles in oncogenic signaling pathways and documented involvement in therapeutic resistance mechanisms. This systematic approach ensured that the analysis focused on biologically relevant targets with potential clinical significance in Ph+ B-ALL treatment resistance.

#### 4.2.2. Protein–Protein Interaction (PPI) Network Construction

Protein–protein interaction networks were constructed using a multi-platform approach to ensure comprehensive coverage and reliability. The STRING database (https://string-db.org/, version 12.0, 1 August 2024) served as the primary source for interaction data, utilizing Homo sapiens as the target species, with a confidence score threshold of ≥0.7 to include only high-confidence interactions. The interaction sources encompassed experimental evidence, database annotations and computational predictions to provide a comprehensive representation of molecular interactions.

TSV interaction data were imported into Cytoscape v3.10.3 (https://cytoscape.org/, The Cytoscape Consortium, Seattle, WA, USA) for network visualization and topological analysis. Networks were refined by removing irrelevant nodes and filtering for oncogenic signaling pathway interactions.

#### 4.2.3. Network Topology Analysis

Network centrality measures were calculated using Cytoscape’s Network Analyzer plugin to identify hub nodes and critical interaction partners within the constructed networks. The analysis incorporated degree centrality to quantify the number of direct connections per node, betweenness centrality to measure the frequency of nodes appearing on shortest paths between other nodes, closeness centrality to assess the average distance from each node to all other nodes in the network, and clustering coefficient to evaluate the degree of neighborhood connectivity around each node.

These topological parameters were systematically employed to prioritize nodes based on their structural importance within the network architecture and their potential as therapeutic targets. Nodes with high centrality scores were considered as key regulatory elements that could significantly impact network stability and cellular function when targeted therapeutically. It should be noted that network topology analysis is inherently descriptive and computational; nodes identified as hubs based on centrality metrics require subsequent functional validation to confirm biological relevance.

#### 4.2.4. Functional Enrichment Analysis

Gene Ontology (GO) and KEGG pathway enrichment analyses were conducted using ShinyGO v0.77 (https://bioinformatics.sdstate.edu/go/, 6 August 2024) for Homo sapiens with FDR < 0.05. Biological Process (GO-BP), Cellular Component (GO-CC) and Molecular Function (GO-MF), and the top 30 enriched terms were visualized to identify overrepresented processes associated with Ph+ B-ALL resistance mechanisms.

### 4.3. Experimental Validation

#### 4.3.1. Cell Culture and Drug Treatment

Philadelphia chromosome-positive B-ALL cell line (SUP-B15) was obtained from ATCC (Manassas, VA, USA) and cultured in Iscove’s Modified Dulbecco’s Medium (IMDM), supplemented with 20% fetal bovine serum (FBS) and 1% penicillin-streptomycin at 37 °C in a humidified atmosphere containing 5% CO_2_. For therapeutic screening, cells were treated for 24 h with targeted inhibitors purchased from Sigma-Aldrich (St. Louis, MO, USA), including Rapamycin (mTOR inhibitor, Cat. No. 553210), GDC-0941 (PI3K inhibitor, Cat. No. 5092260001), GSK690693 (pan-Akt inhibitor, Cat. No. SML0428), and Perifosine (Akt inhibitor, Cat. No. SML0612). Each inhibitor was initially prepared as a 10 mM stock solution in dimethyl sulfoxide (DMSO). The inhibitor concentrations used for the 24-h treatment and phospho-kinase array were selected based on previously reported drug-sensitivity/IC_50_ data, including SUP-B15-specific values, where available, and further evaluated using an independent 24-h MTT-based dose-response experiment. Cell viability data were analyzed by nonlinear regression to estimate IC_50_ values, and the final concentrations were selected based on the combined literature/database and experimental findings: rapamycin (9 µM), GDC-0941 (9 µM), GSK690693 (20 µM), and perifosine (10 µM).

#### 4.3.2. Onco-Proteome Array Analysis (Phospho-Kinase Profiling)

Phospho-kinase profiling was performed using the Proteome Profiler™ Human Phospho-Kinase Array Kit (Catalog #ARY003C, R&D Systems, Minneapolis, MN, USA) according to the manufacturer’s instructions. Two independent biological replicates were analyzed per treatment group (Control, Perifosine, GDC-0941, GSK690693, and Rapamycin), each corresponding to an independently prepared cell lysate processed on a separate array membrane. SUBP-15 cells were treated with each compound at its IC_50_ concentration for 24 h prior to lysis. Cell lysates were prepared and protein concentrations determined using the Pierce™ Bradford Protein Assay Kit (Cat#23200, Thermo Scientific, Rockford, IL, USA). Equal amounts of protein from each lysate were incubated overnight at 4 °C with pre-blocked array membranes.

Background signal was subtracted using the mean density of PBS-loaded negative control reference spots on each membrane, and signal intensities were subsequently normalized to membrane-specific positive reference spots to correct for inter-membrane variation. Membranes were imaged using the ChemiDoc™ XRS+ Gel Documentation System (Bio-Rad, Hercules, CA, USA) and analyzed using Image Lab Software version 6.1 (Bio-Rad). For each phosphoprotein, normalized signal intensities from treated samples were divided by the corresponding normalized signal intensity from the untreated control to yield fold-change values, which were then averaged across the two biological replicates. For hierarchical clustering and heatmap visualization, averaged fold-change values were z-score normalized across all treatment groups for each phosphoprotein; accordingly, the heatmap presented in Figure 3 represents z-score-transformed fold-change values and does not display absolute signal intensities or raw densitometric measurements. Raw densitometric values for each individual biological replicate are provided in Supplementary Table S15.

#### 4.3.3. Patient Sample Collection

This study enrolled 100 patients with Philadelphia chromosome-positive B-cell acute lymphoblastic leukemia (B-ALL) for RT-PCR analysis and 50 patients for Western blot validation. Control samples for the Western blot analysis were obtained from 7 healthy volunteers. Eligibility criteria required a confirmed diagnosis of Philadelphia chromosome-positive B-ALL and availability of complete clinical and follow-up data (Supplementary Table S16). Ethical approval for sample collection and processing was obtained from the ICMR-NICHDR (formerly ICMR-NIP), New Delhi, and the Institutional Ethics Committee of VMMC & Safdarjung Hospital, New Delhi. Written informed consent was obtained from all participants or next of kin prior to enrolment.

#### 4.3.4. Sample Processing

Peripheral blood samples were collected from Philadelphia chromosome-positive (Ph+) B-ALL patients (*n* = 100) and healthy controls (*n* = 5) using EDTA-coated vacutainers (366643, Becton, Dickinson and Company, Berkshire, UK). For RT-qPCR analysis, each patient sample represented an independent biological replicate. Healthy control samples and SUBP-15 cell line replicates were included as comparators; the number and replicate type for each group are detailed in Supplementary Table S17. For immunoblotting analyses, PBMC samples were obtained from 50 independent Ph+ B-ALL patients using Histopaque density-gradient centrifugation. PBMC samples were also obtained from 7 healthy volunteers, with each healthy control sample analyzed independently. Thus, a total of 57 independent biological samples (50 B-ALL patients and 7 healthy controls) were included in the immunoblotting analysis. Plasma was separated by centrifugation at 2000× *g* for 10 min at 4 °C, aliquoted, and stored at −80 °C until use. All patient and control samples were collected between 2021 and 2025.

#### 4.3.5. Patient Sample Collection for RT-PCR

Peripheral whole blood samples were collected from patients diagnosed with Ph+ve ALL (*n* = 100) and healthy controls (*n* = 15) in EDTA-coated vacutainers. Total RNA was extracted using the QIAamp RNA Blood Mini Kit (Qiagen, Hilden, Germany, Cat. No. 52304) according to the manufacturer’s protocol. The purity and concentration of isolated RNA were assessed using a NanoDrop LITE spectrophotometer (Thermo Fisher Scientific, Waltham, MA, USA).

#### 4.3.6. cDNA Synthesis

First-strand complementary DNA (cDNA) synthesis was performed using the High-Capacity cDNA Reverse Transcription Kit (Applied Biosystems, Foster City, CA, USA, Cat. No. 4368814). A total of 2 µg of RNA was used in a 20 µL reaction volume. Thermal cycling conditions were programmed as follows: 25 °C for 10 min, 37 °C for 120 min, and 85 °C for 5 min, followed by cooling at 4 °C.

#### 4.3.7. Quantitative Real-Time PCR (qRT-PCR)

Quantitative expression analysis of GSK3A, GSK3B, and CREB1 was performed using the PowerTrack™ SYBR Green Master Mix (Applied Biosystems, Cat. No. A46109) in a final reaction volume of 20 µL, with ABL1 serving as the housekeeping gene. Amplification reactions were carried out on the StepOnePlus™ Real-Time PCR System (Applied Biosystems) under the following conditions: an initial denaturation at 95 °C for 2 min, followed by 40 cycles of denaturation at 95 °C for 15 s and annealing/extension at 60 °C for 1 min. The primers used for amplification were synthesized based on sequences obtained from the Harvard Primer Bank. The forward and reverse primer sequences were as follows: GSK3A (forward 5′-GCAGATCATGCGTAAGCTGGAC-3′, reverse 5′-GGTACACTGTCTCGGGCACATA-3′), GSK3B (forward 5′-CCGACTAACACCACTGGAAGCT-3′, reverse 5′-AGGATGGTAGCCAGAGGTGGAT-3′), CREB1 (forward 5′-GCTGCCTCTGGAGACGTACAA-3′, reverse 5′-GCTAGTGGGTGCTGTGCGA-3′), and ABL1 (forward 5′-CATCACGCCAGTCAACAGTCT-3′, reverse 5′-GTACACCCTCCCTTCGTATCT-3′). The relative expression levels of target genes were calculated using the 2^−ΔΔCt^ method, with normalization performed against ABL1 (Supplementary Table S18).

#### 4.3.8. Patient Sample Collection for Western Blot

Peripheral blood samples were obtained from 50 Philadelphia chromosome-positive (Ph+) patients diagnosed with B-cell acute lymphoblastic leukemia (B-ALL) using EDTA-coated vacutainers. Peripheral blood mononuclear cells (PBMCs) were isolated by density-gradient centrifugation using Ficoll-Paque. The PBMC layer was carefully collected, washed twice with phosphate-buffered saline (PBS), and subsequently aliquoted, lysed and stored at −80 °C until further use. For control experiments, PBMC samples from 7 healthy individuals were collected and processed under identical conditions. Each patient sample and each healthy control sample constituted one independent biological replicate. No technical replicates were performed. All patient and control samples were collected between 2021 and 2025. Ethical approval for the study was obtained from the ICMR-NICHDR (formerly ICMR-NIP), New Delhi, and the VMMC & Safdarjung Hospital, New Delhi Ethical Committees.

#### 4.3.9. Western Blot (Immunoblotting) Analyses

The total protein concentration in cell lysate of PBMNC was measured using the Pierce™ Bradford Protein Assay Kit (Thermo Scientific, Rockford, IL, USA, Cat. No. 23200), following the manufacturer’s instructions. Equal amounts of total protein (30 µg per sample) were loaded for each sample, resolved on 12% SDS-PAGE, and transferred onto 0.2 μm PVDF membranes (Millipore, Burlington, MA, USA). Membranes were blocked with Blocking Buffer (Himedia, Thane, Maharashtra, India, Cat. No. ML044) for 2 h at room temperature and then incubated overnight at 4 °C with primary antibodies from Cell Signaling Technology (Danvers, MA, USA), Phospho-CREB (Ser133) (87G3) Rabbit mAb (Cat. No. 9198) and Phospho-GSK-3α/β (Ser21/9) (D17D2) Rabbit mAb (Cat. No. 8566). β-actin was used as the loading control for normalization of the corresponding target-protein signal. After washing, membranes were incubated with anti-rabbit IgG, HRP-linked (Cat. No. 7074; Cell Signaling Technology, Danvers, MA, USA; 1:1000) for 2 h at room temperature. Protein bands were visualized using an ECL detection kit (Takara Bio Inc., Kusatsu, Japan), and images were acquired with the ChemiDoc XRS+ system (Bio-Rad, Hercules, CA, USA). Band intensities were quantified using Image Lab Software (Bio-Rad). Background-adjusted p-CREB(Ser133) and p-GSK-3α/β(Ser21/9) band intensities were normalized to the corresponding β-actin intensity for each sample. Note: a separate post-transfer total-protein normalization procedure, such as Ponceau S staining or stain-free total-protein imaging, was not performed in this study and was therefore not used for densitometric normalization.

#### 4.3.10. Statistical Analysis

##### For Onco-Proteome Array

For statistical analysis, background-subtracted, positive-control-normalized signal intensities were analyzed for 39 phosphoprotein targets. An unpaired two-sided Student’s *t*-test was applied independently to each target using an equal-variance assumption (degrees of freedom = 2), without pooling variance across targets. Fold-change values, calculated as the ratio of the drug-treated mean to the mean normalized signal intensity of the untreated control, were used for reporting; normalized signal intensities served as the input for statistical testing. To control for multiple comparisons, the Benjamini–Hochberg false discovery rate (BH–FDR) procedure was applied separately within each drug versus untreated control comparison, constituting four independent testing families of 39 tests each. Statistical significance was defined as a BH-adjusted Q ≤ 0.05. Complete statistical results, including raw *p*-values, BH-adjusted *q*-values, fold-change values, and FDR significance classifications, are provided in the Supplementary Tables S14 and S15.

##### For RT-qPCR Study

Data analysis was conducted using GraphPad Instat 10.2 software (GraphPad, Inc., San Diego, CA, USA). Results are expressed as mean ± standard deviation (SD). Prior to between-group comparisons, data normality was assessed for each gene using the Shapiro–Wilk test. In all three genes, data from the patient cohort (*n* = 100) violated the normality assumption (GSK3α: W = 0.7832, *p* < 0.0001; GSK3β: W = 0.6097, *p* < 0.0001; CREB1: W = 0.5599, *p* < 0.0001), whereas the control and cell line groups (*n* = 5 each) satisfied normality. Given this violation, non-parametric tests were applied throughout. Statistical comparisons across the three groups (Ph+ B-ALL patients, healthy controls, and SUBP-15 cell line) were performed using the Kruskal–Wallis test, followed by Dunn’s post hoc test with Bonferroni correction for multiple pairwise comparisons. A *p*-value < 0.05 was considered statistically significant. For graphical presentation, all RT-qPCR figures display individual data points overlaid on box plots, with statistical significance annotations (exact adjusted *p*-values or significance brackets) shown directly on each figure panel.

##### For Western Blot

Quantitative Western blot data for CREB1 and GSK3α/β were analyzed by densitometry using Bio-Rad software, version 6.1. Background-adjusted protein-of-interest band intensities were normalized to the corresponding beta actin intensity for each sample. Because the normalized expression data were right-skewed, values were log2-transformed before inferential analysis. The primary analysis used a parametric linear model including disease status (healthy control versus disease) as the biological factor and membrane/blot identity as a technical blocking factor. The effect of disease versus control was estimated after adjustment for membrane, with two-sided *p*-values and 95% confidence intervals; the estimated effect was back-transformed to obtain the fold difference between groups.

For graphical presentation, expression was reported as fold change relative to the healthy control on the corresponding membrane, with the control assigned a value of 1.0. Data were presented as individual observations with box plots, and the median, interquartile range, geometric mean, and arithmetic mean were used as descriptive measures. A logarithmic y-axis was used where appropriate. As a sensitivity analysis, a two-sided Mann–Whitney U test was performed to compare the healthy control and disease groups. The membrane-adjusted linear model was considered the primary analysis because it accounted for both data skewness and variation between membranes. All tests were two-sided, with *p* < 0.05 considered statistically significant. No observations were excluded solely because of their magnitude; high-expression values were retained unless an independent experimental or technical reason for exclusion was established. Since equal protein loading was determined by Bradford assay rather than by post-transfer total-protein staining, the densitometric normalization was based on β-actin rather than total transferred protein. Additional details regarding the calculation and assessment of variation between patient samples and within membranes are provided in the Supplementary Materials.

## 5. Conclusions

In this study, we employed a multi-platform approach—integrating phospho-kinase profiling of SUP-B15 cells, RT-PCR analysis of 100 patient samples, Western blotting of 50 patient samples, and protein–protein interaction (PPI) network analysis—to characterize adaptive signaling responses to PI3K/Akt/mTOR inhibition in Philadelphia chromosome-positive B-cell acute lymphoblastic leukemia (Ph+ B-ALL). Our data identify CREB1 and GSK3α/β as high-centrality signaling integration nodes that exhibit compensatory activation following targeted pathway blockade.

Our findings reveal a complex regulatory landscape for these candidates. In SUP-B15 cells, treatment with mechanistically distinct PI3K, Akt, and mTOR inhibitors led to a statistically significant increase in phospho-CREB (Ser133) and phospho-GSK3α/β (Ser21/9) levels, suggesting a cross-inhibitor adaptive response. Interestingly, while CREB1, GSK3α, and GSK3β mRNA expression was significantly downregulated (61–92% reduction) in Ph+ B-ALL patient samples compared to healthy controls, phospho-CREB (Ser133) protein levels were significantly elevated (demonstrating a 2.49-fold increase, *p* = 0.0023). This pronounced mRNA–phosphoprotein decoupling indicates the presence of robust post-transcriptional and post-translational regulatory mechanisms that maintain oncogenic signaling despite transcriptional repression.

The biological relevance of these nodes is further supported by PPI network analysis, which placed CREB1 and GSK3α/β in high-centrality positions with extensive connectivity to key survival drivers, including AKT1, MAPK1/3, STAT3, TP53, NF-κB, and MYC. These results are consistent with the known cellular functions of p-CREB (Ser133) in the transcriptional activation of anti-apoptotic and metabolic programs, and the role of p-GSK3α/β (Ser21/9) in stabilizing oncogenic proteins like c-Myc and MCL-1. Collectively, these findings contribute to the paradigm of adaptive kinome reprogramming, wherein cancer cells circumvent therapeutic blockade through compensatory signaling. The identification of CREB1 and GSK3α/β extends this model to a clinically significant leukemia subtype. However, it is important to emphasize that our current evidence demonstrates association rather than direct functional causality. These findings do not yet establish whether these nodes are necessary or sufficient for therapeutic resistance. Consequently, we position CREB1 and GSK3α/β as candidate adaptive response markers and potential targets for combination therapy. Further functional validation remains essential, including: (1) genetic perturbation via CRISPR/siRNA to assess sensitization; (2) pharmacological co-inhibition studies; (3) identification of the specific upstream kinases mediating these compensatory events; (4) correlation of these markers with clinical drug sensitivity; and (5) validation in patient-derived xenograft (PDX) models. These steps will be critical in translating these associative observations into actionable therapeutic strategies for Ph+ B-ALL.

## Data Availability

Some of the datasets generated and/or analyzed during this study are not publicly available due to patient confidentiality and the sensitive nature of clinical data. However, de-identified data supporting the findings of this study are provided in the Supplementary Materials. If further data are required, these can be made available by the corresponding author upon reasonable request.

## Supplementary Materials

The following supporting information can be downloaded at: https://www.mdpi.com/article/10.3390/ijms27188300/s1.
