# Supplementary material for: CREB1 and GSK3 Isoforms as Potential Adaptive Signaling Nodes in PI3K/Akt/mTOR Inhibitor Treated Philadelphia Chromosome-Positive B-ALL
Source: Int J Mol Sci. 2026 Sep 17;27(18):8300. doi: 10.3390/ijms27188300 (PMC13607625; doi:10.3390/ijms27188300)
Supplement: Supplementary file 1 [file ijms-27-08300-s001.zip › ijms-4433073-supplementary.pdf]

Supplementary Figures S1-S7

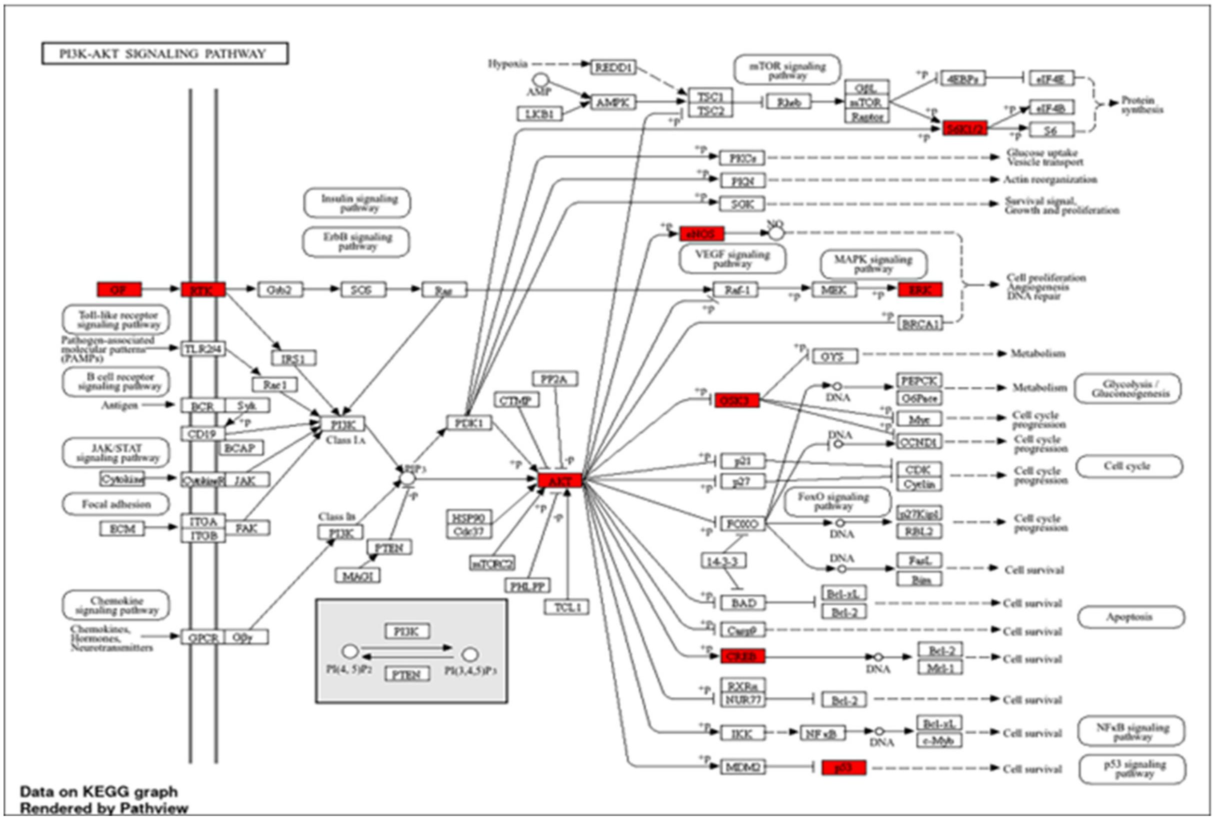

(A)

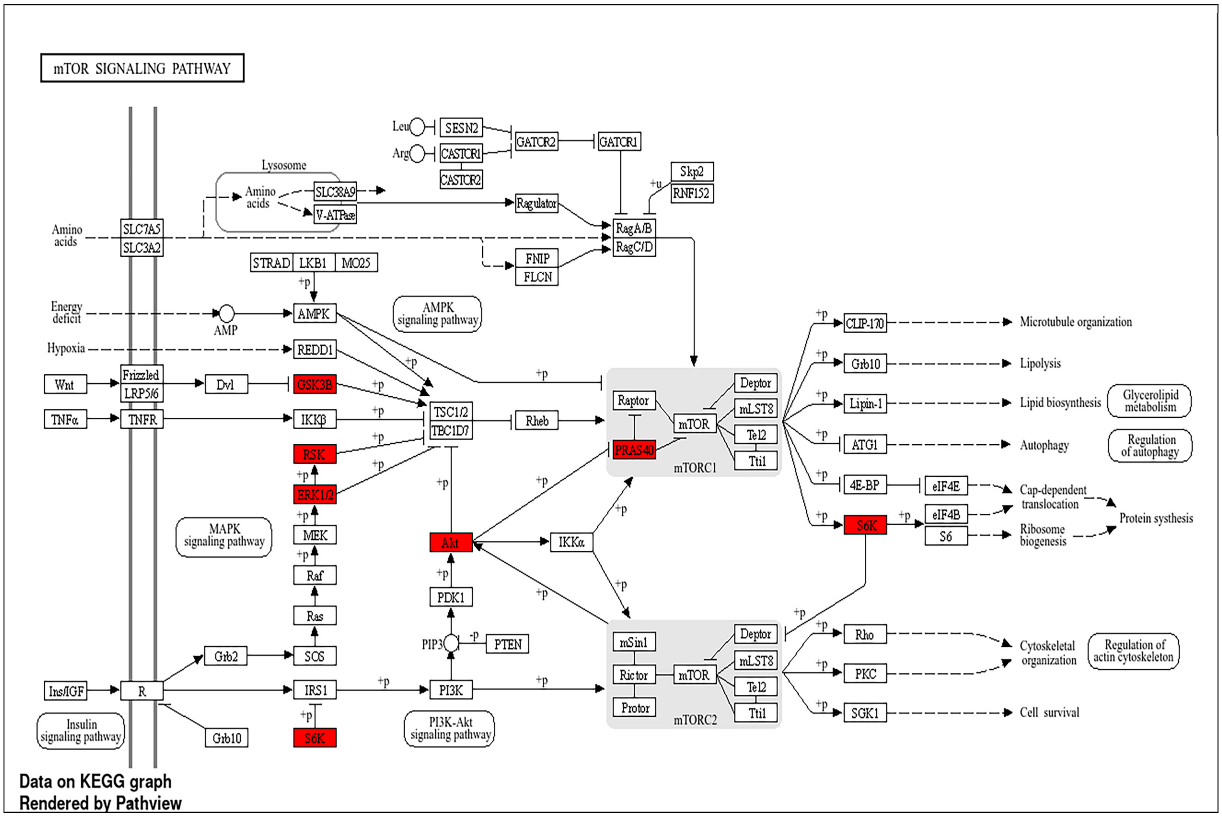

(B)

(C)

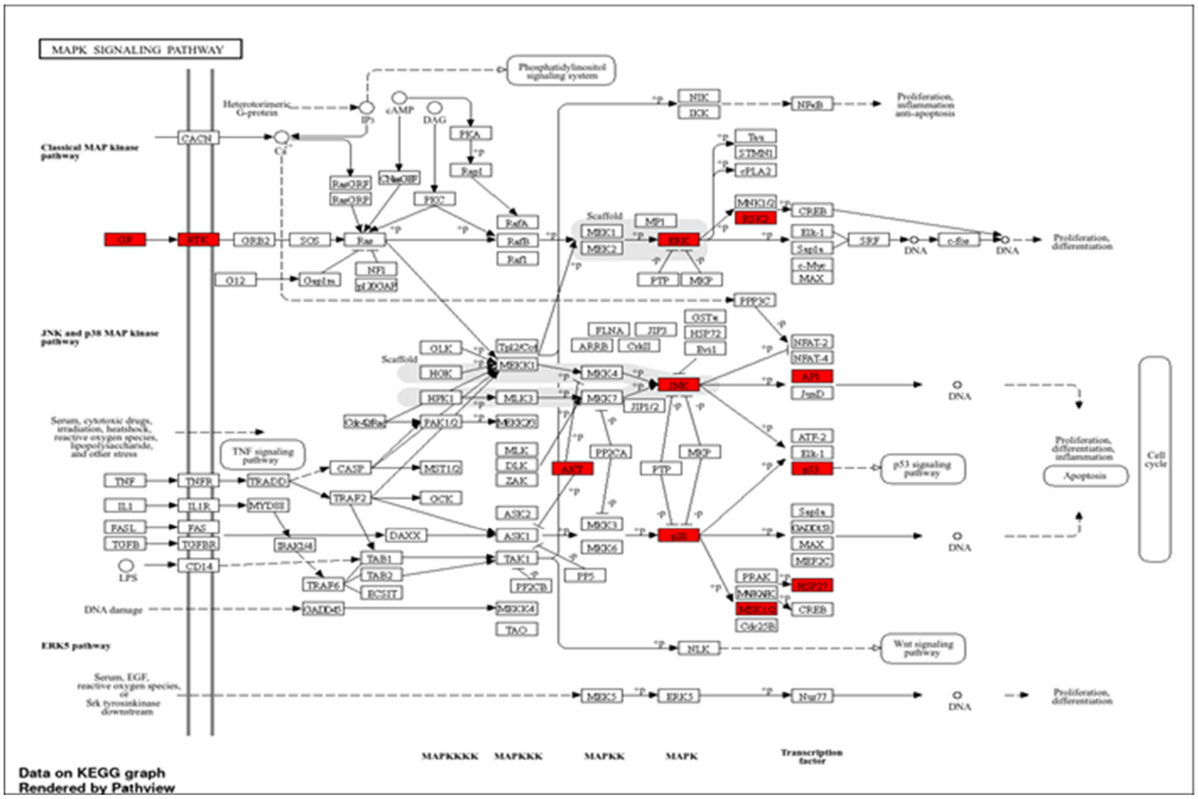

Figure S1 . KEGG illustrating diagram of biological pathways for T2DM. **A-C.** All the key targets were marked in red.

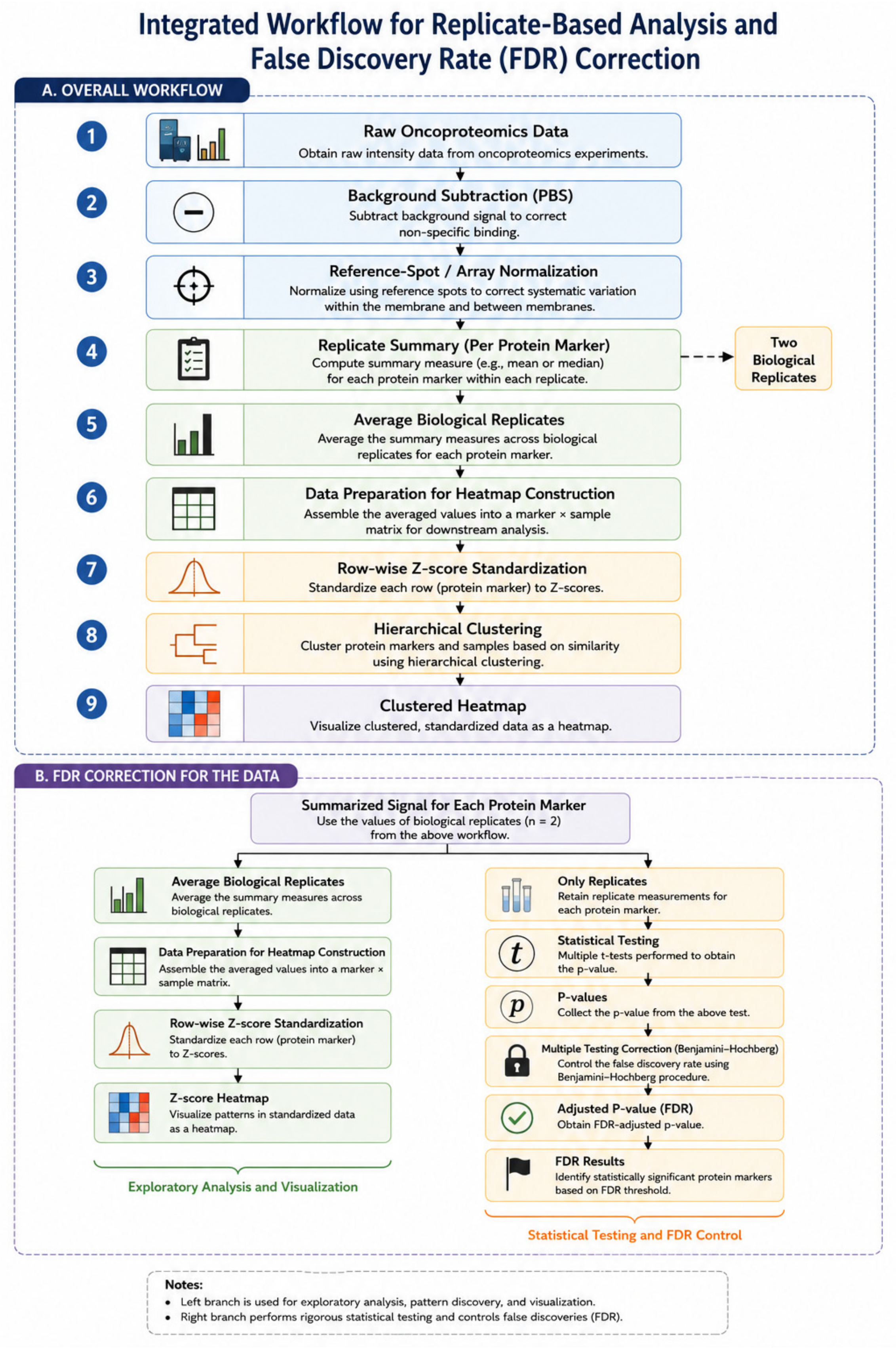

Figure S2. Integrated workflow for replicate-based analysis, visualization, and false discovery rate

(FDR) correction of Oncoproteomics data. (A) Overall analytical workflow showing sequential processing of raw oncoproteomics intensity data, including background subtraction using PBS, reference-spot/array normalization to account for systematic variation within and between membranes, summary of measurements for each protein marker within biological replicates, averaging across biological replicates, and preparation of the protein marker × sample matrix. Row-wise Z-score standardization is subsequently performed for visualization, followed by hierarchical clustering and generation of the clustered heatmap. (B) FDR correction workflow using the summarized protein-marker measurements from two biological replicates (n = 2). The averaged biological replicates are used for exploratory visualization and heatmap generation, whereas replicate-level measurements are retained for statistical testing. Multiple t-tests are performed for the individual protein markers, followed by Benjamini–Hochberg correction for multiple comparisons. Adjusted p-values are used to identify protein markers meeting the predefined FDR significance threshold. The two branches therefore distinguish exploratory data visualization from statistical testing and FDR control.

- **Details of the program cycle used for the run**

Real-time PCR amplification for **CREB1, GSK3A, and GSK3B** was performed using SYBR Green chemistry under optimized conditions suitable for multiplex analysis. For CREB1, the cycling program included an initial enzyme activation step at 95 °C for 15 minutes, followed by 40 cycles of denaturation at 95 °C for 15 seconds, annealing at 60 °C for 1 minute, and extension at 72 °C for 1 minute. For GSK3A and GSK3B, primer sets validated by OriGene were amplified using an initial activation at 50 °C for 2 minutes, a pre-soak step at 95 °C for 10 minutes, and 40 cycles consisting of denaturation at 95 °C for 15 seconds and annealing at 60 °C for 1 minute. For multiplex RT-PCR optimization, a unified cycling protocol was applied where appropriate, consisting of an initial activation at 95 °C for 15 minutes followed by 40 cycles of 95 °C for 15 seconds, 60 °C for 1 minute, and 72 °C for 1 minute. A melting curve analysis (65–95 °C, gradual increment) was performed immediately after amplification to verify product specificity and to exclude primer-dimer formation. A no-template control (nuclease-free water) was included in each run to monitor contamination.

Plate - 1

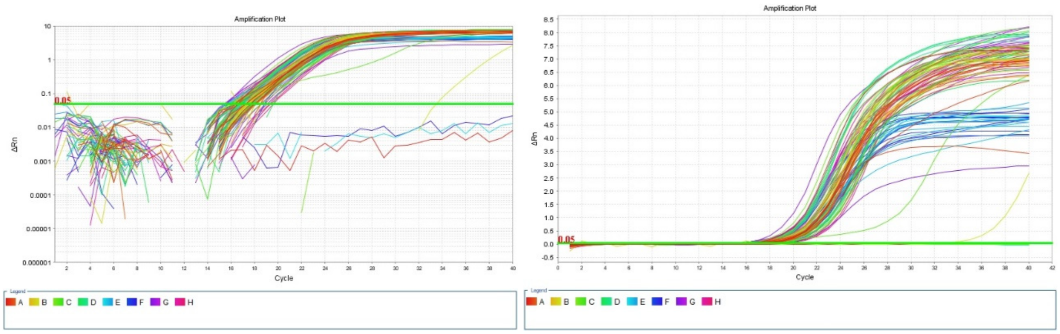

Plate - 2

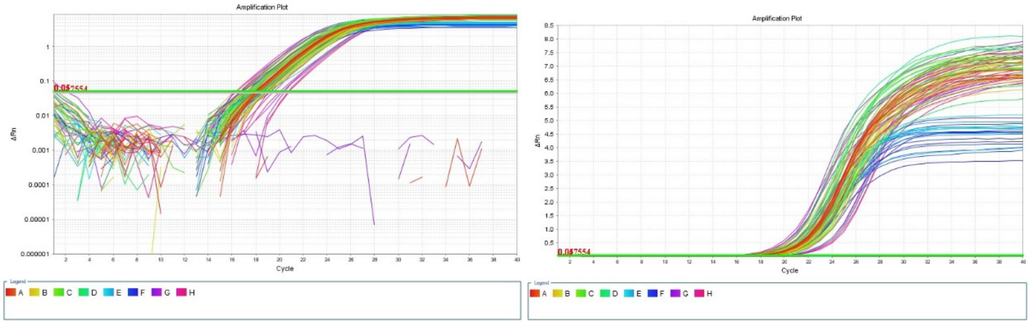

Plate - 3

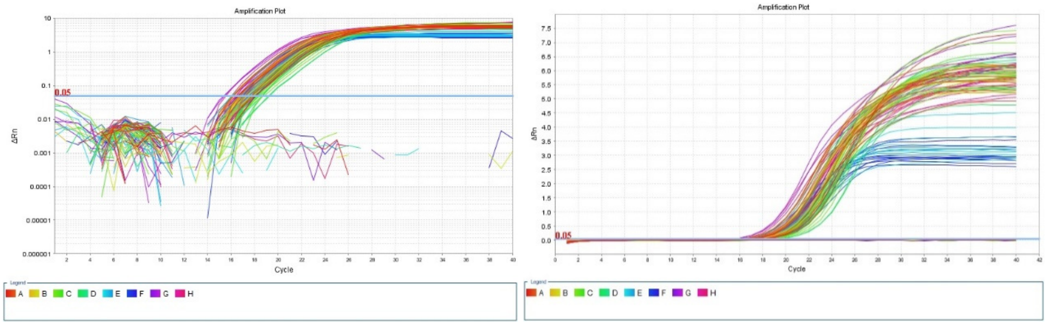

Plate - 4

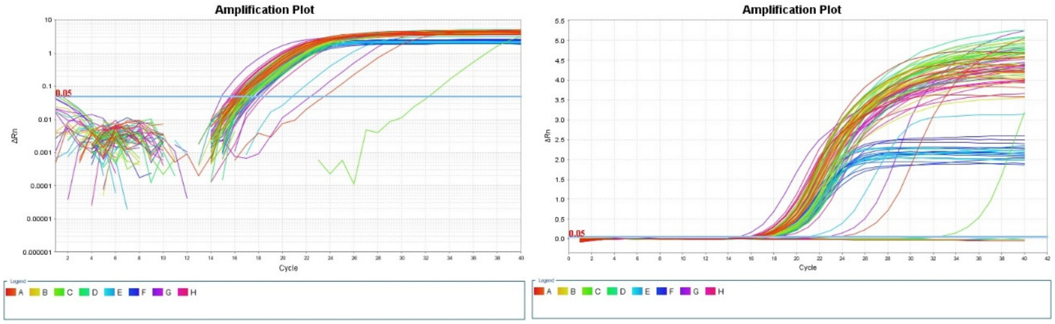

Plate - 5

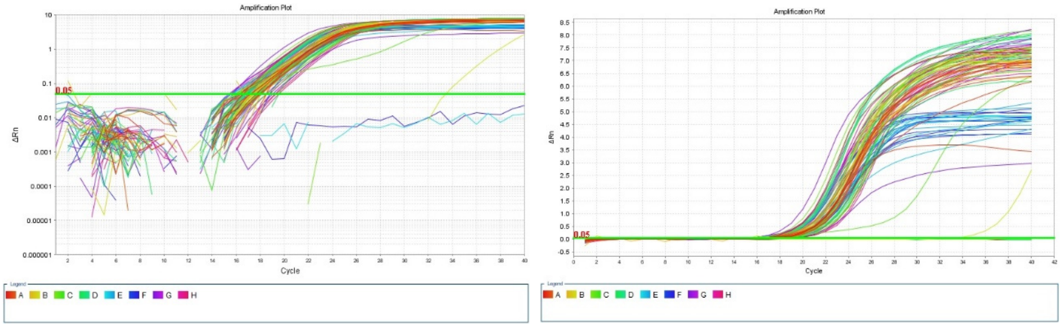

All controls

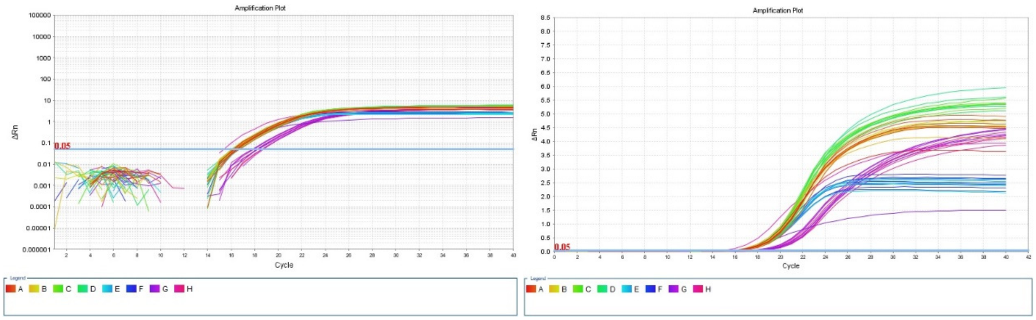

Figure S3: The image represents all the amplification curves that were generated for each target gene and the housekeeping gene across all experimental runs (plates) respectively. The plots show the delta Rn vs cycle, allowing determination of Ct values.

- Supplementary Data for western blot

Ladder used: hi media pre - stained protein ladder

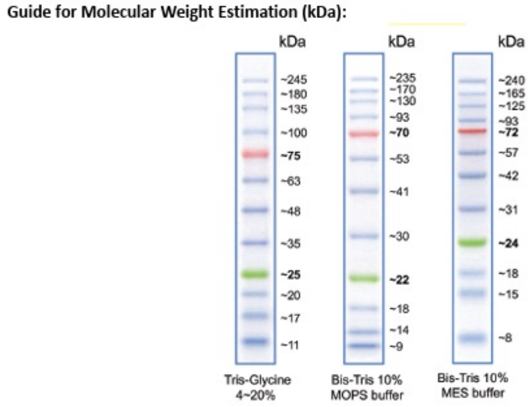

Antibody used :

#8566 Store at -20C

Phospho-GSK-3 alpha/beta (Ser21/9) (D17D2) Rabbit Monoclonal Antibody

Cell Signaling TECHNOLOGY®

Orders: 877-616-CELL (2355)

Support: 877-678-TECH (8324)

Web: cellsignal.com

3 Trask Lane | Danvers | Massachusetts | 01923 | USA

For Research Use Only. Not for Use in Diagnostic Procedures.

Applications:	Reactivity:	Sensitivity:	MW (kDa):	Source/Isotype:	UniProt ID:	Entrez-Gene Id:
W, IP	H M R Hm Mk	Endogenous	46 GSK-3beta, 51 GSK-3alpha	Rabbit IgG	#P49840, #P49841	2931, 2932

P – GSK3alpha/beta

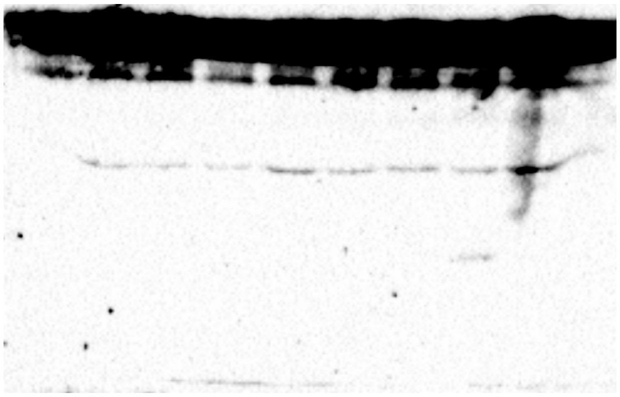

Uncropped image:

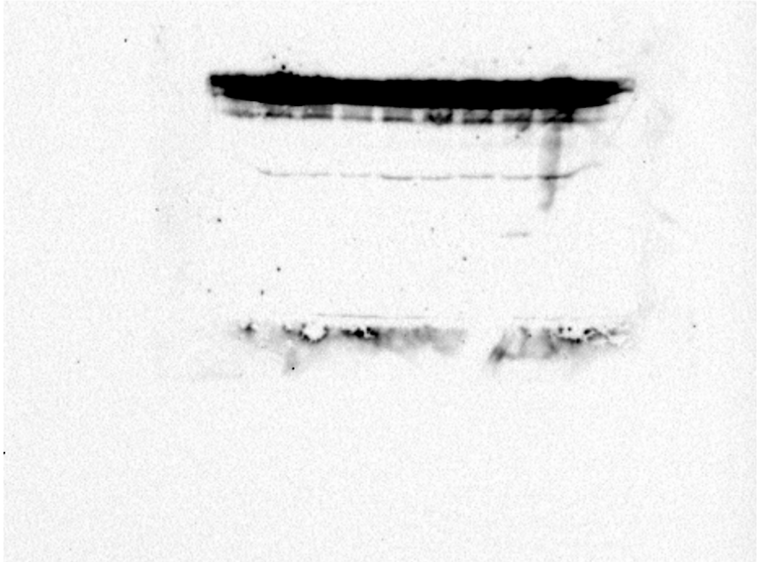

Ladder :

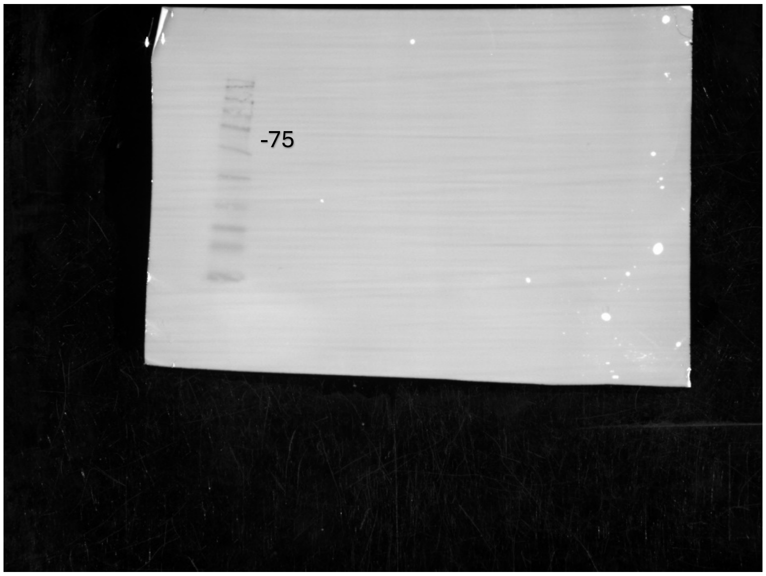

Ladder superimposed :

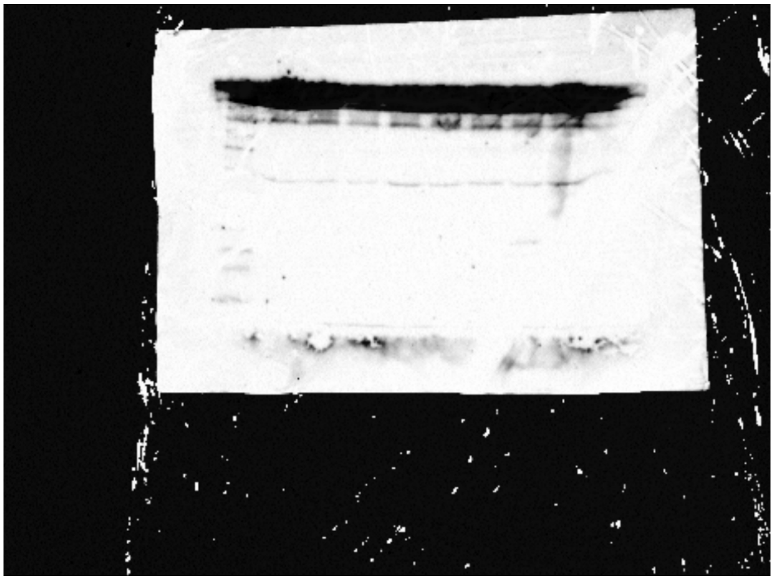

1)

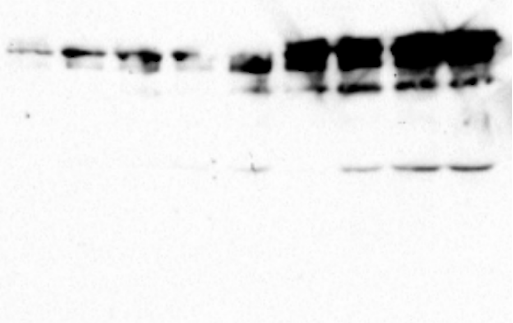

uncropped image :

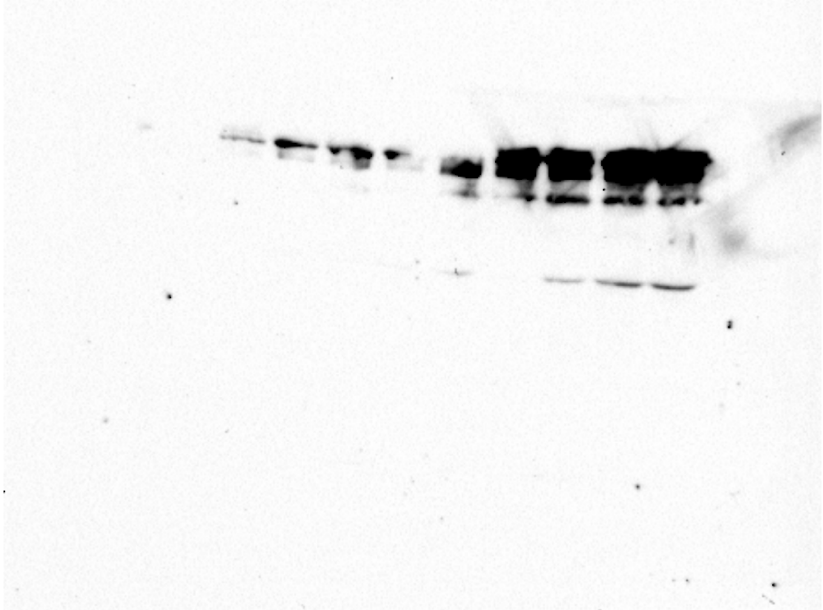

Ladder:

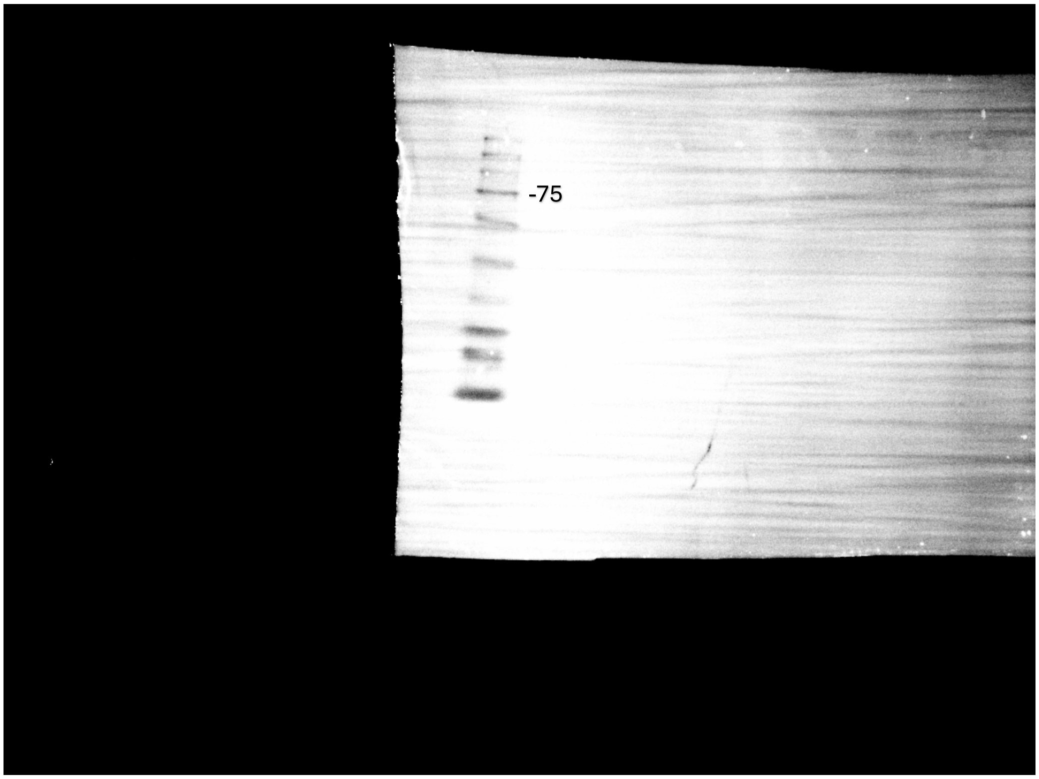

Ladder superimposed :

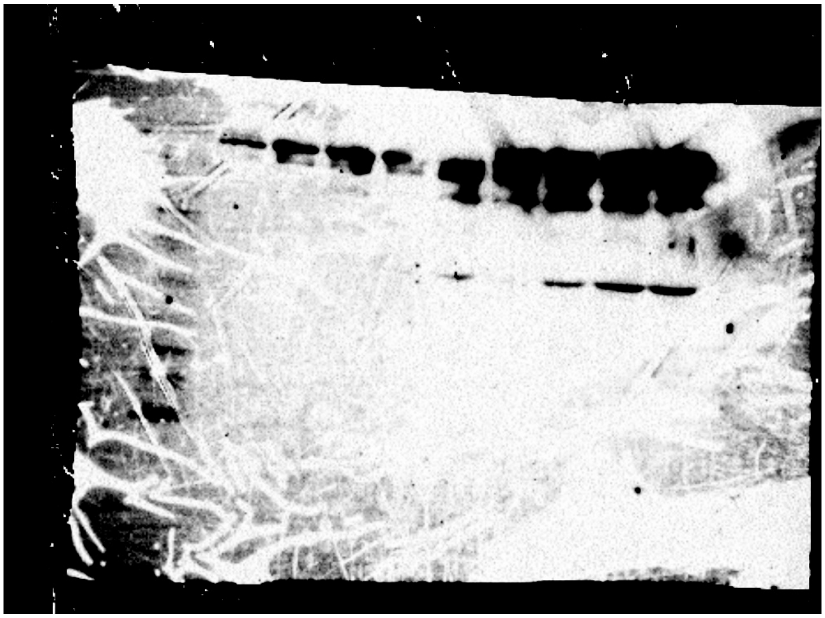

2) 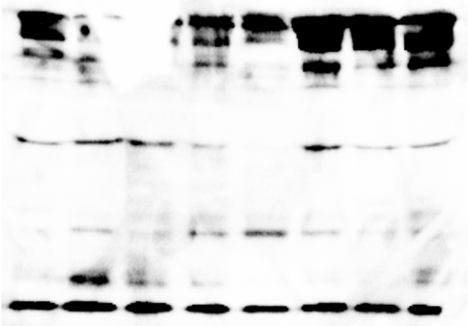

uncropped image :

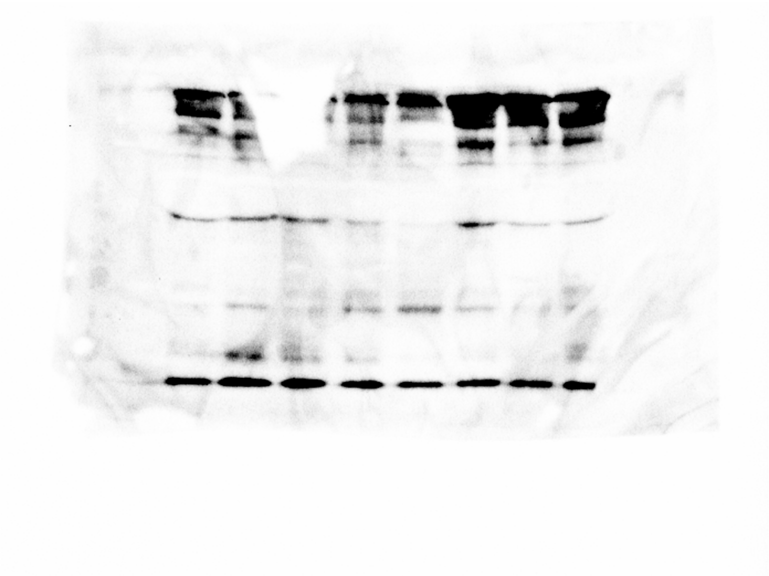

Ladder :

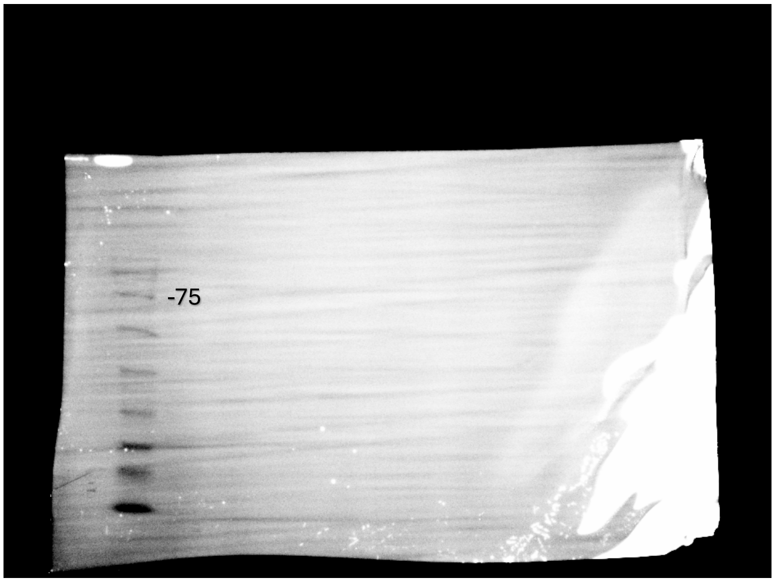

3)

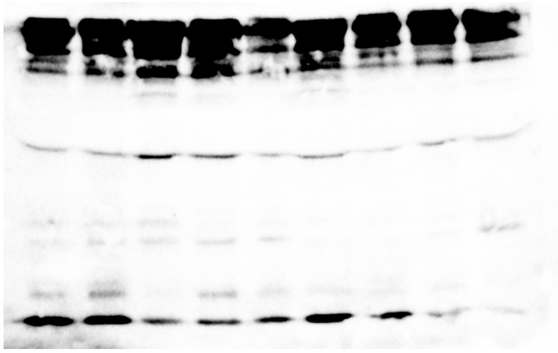

uncropped image :

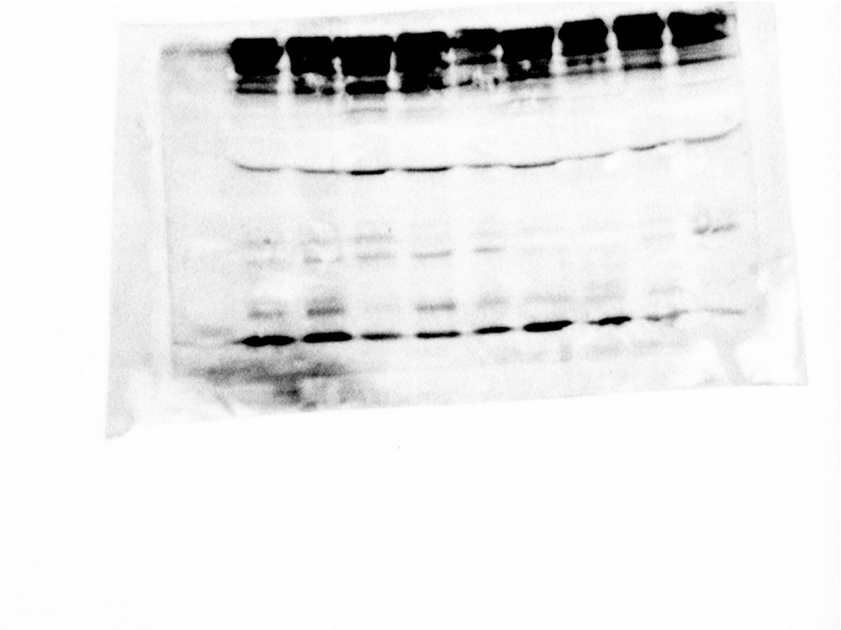

Ladder :

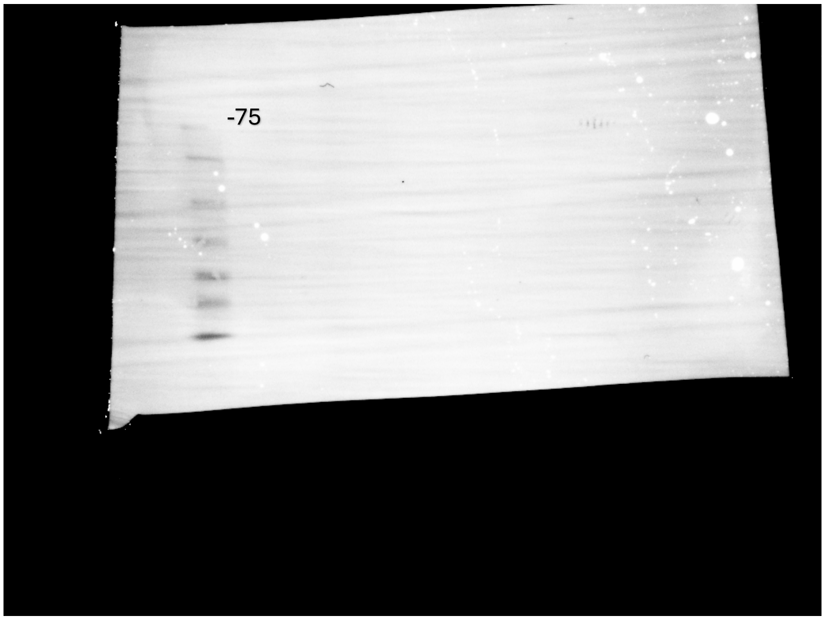

Ladder superimposed :

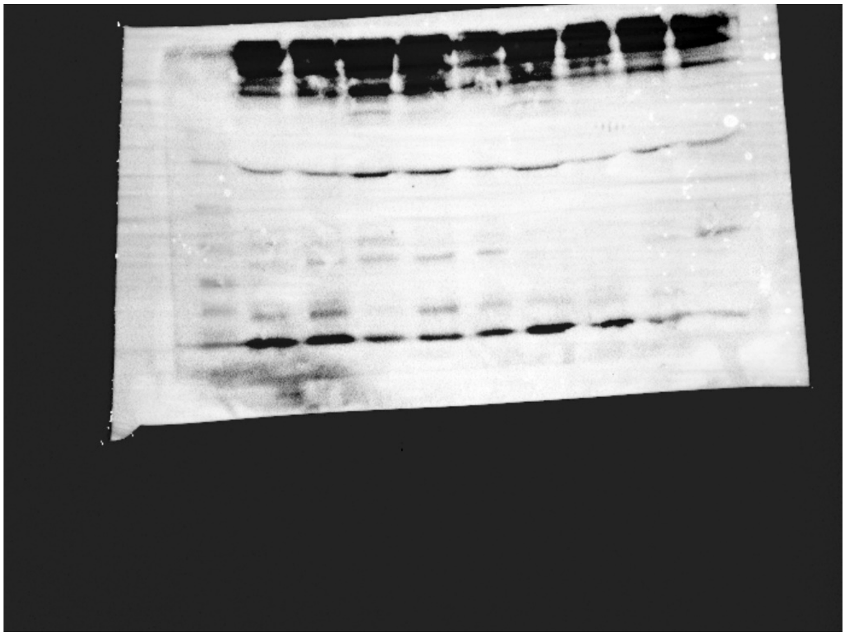

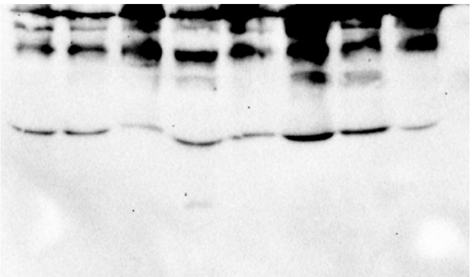

4)

uncropped image :

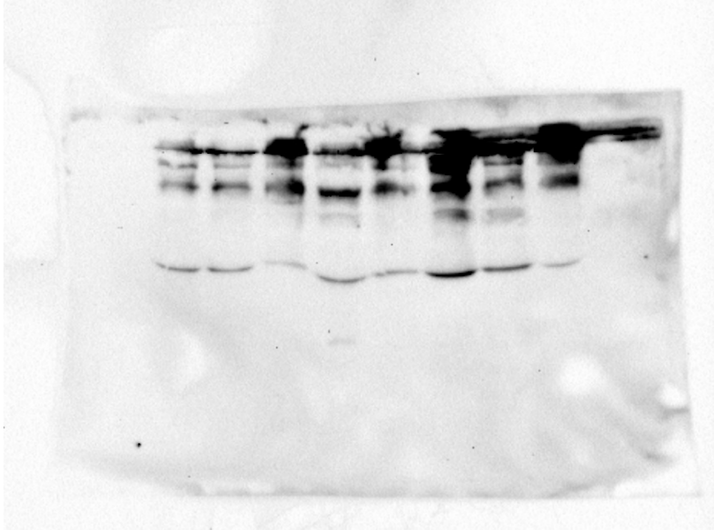

Ladder :

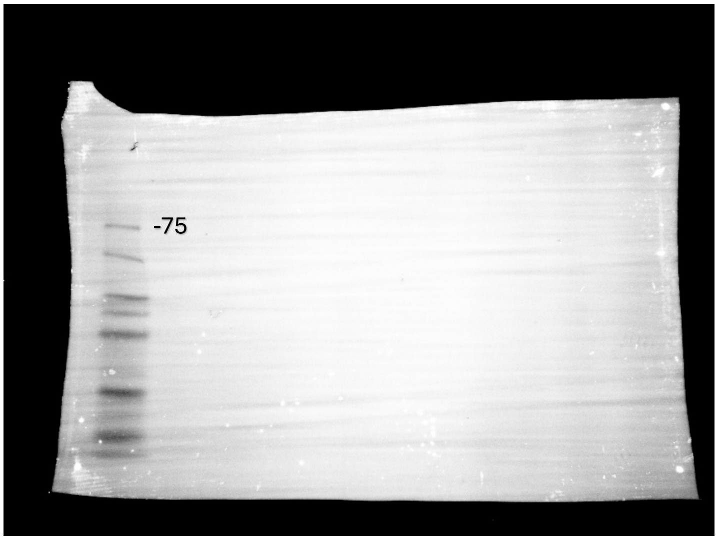

Ladder superimposed :

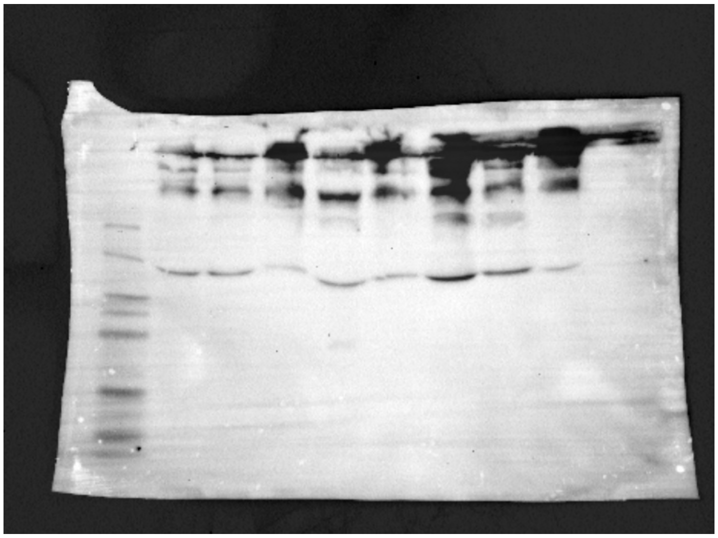

5)

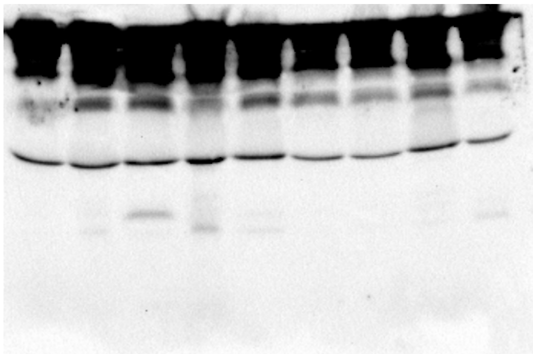

uncropped image :

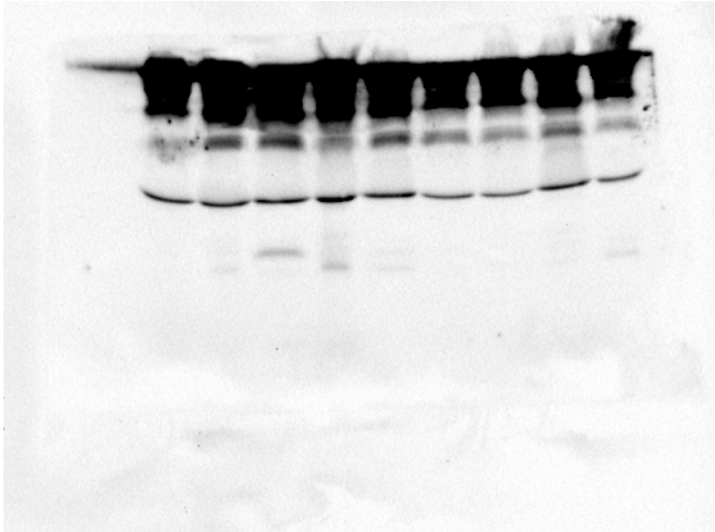

Ladder :

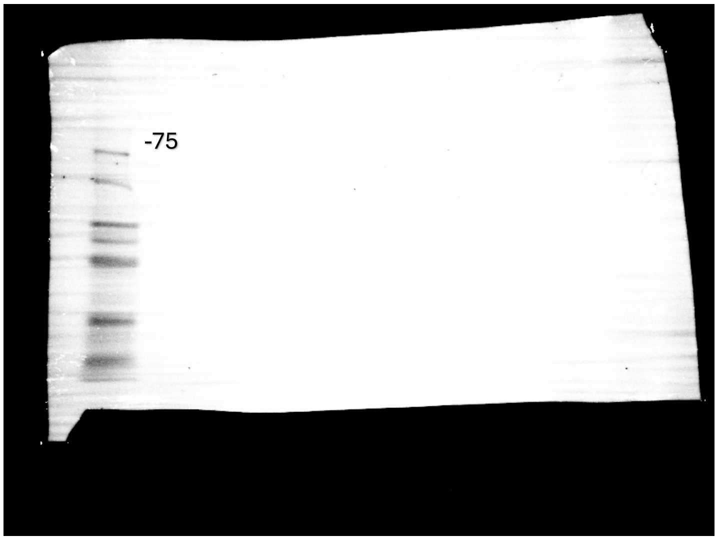

Ladder superimposed :

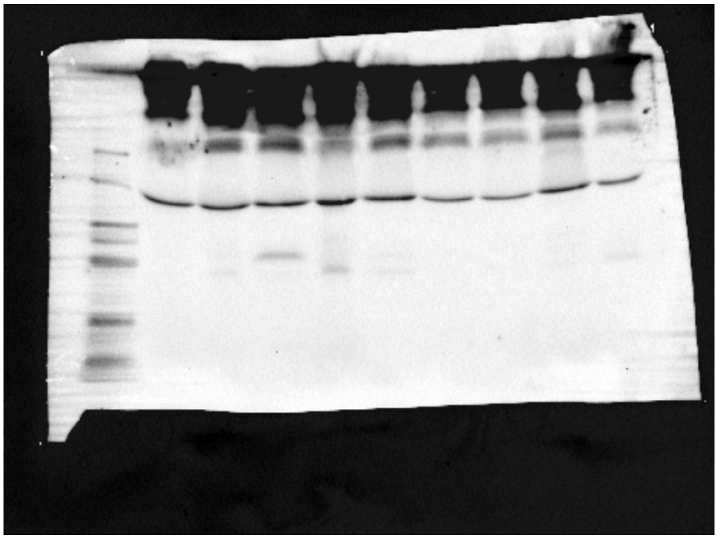

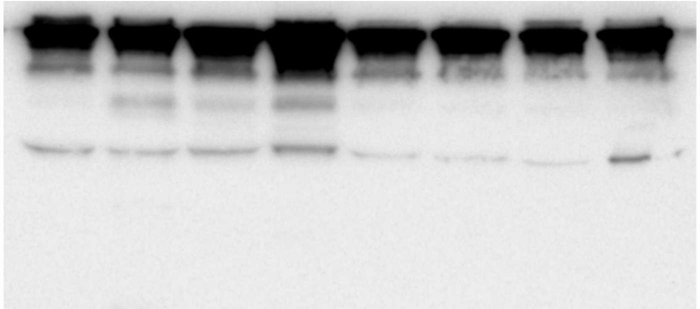

6)

uncropped image :

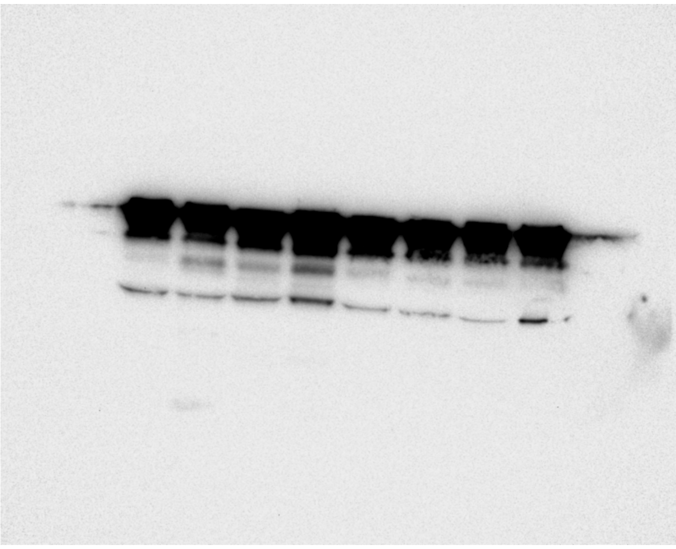

Ladder :

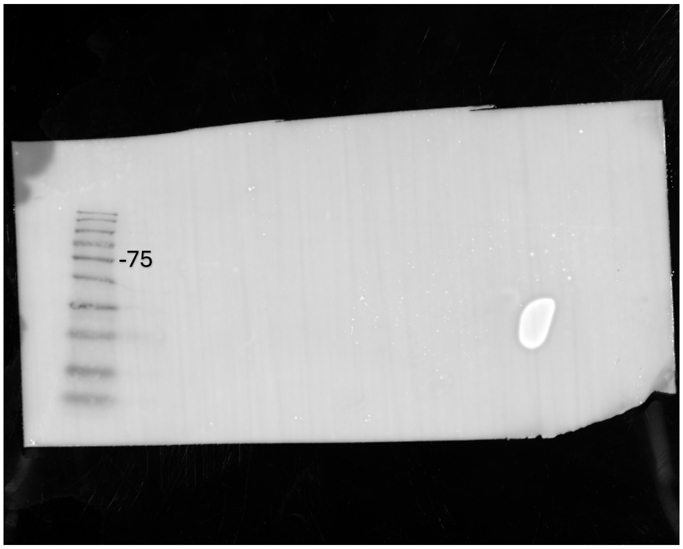

Ladder superimposed:

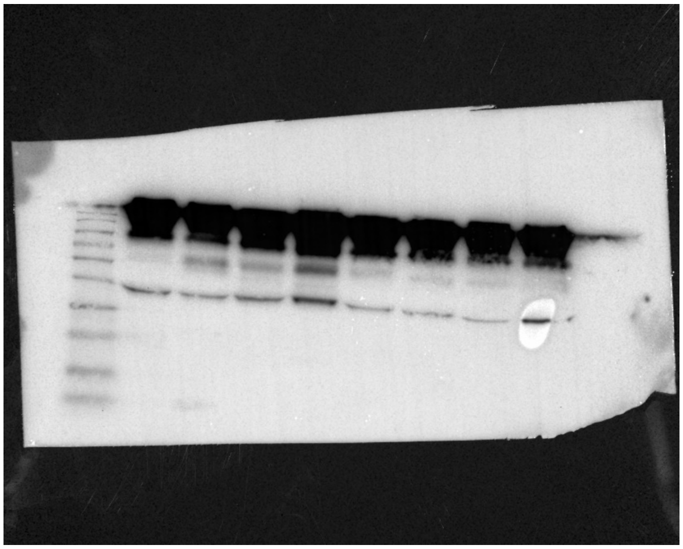

P – CREB

Ladder used: hi media pre - stained protein ladder

Guide for Molecular Weight Estimation (kDa):

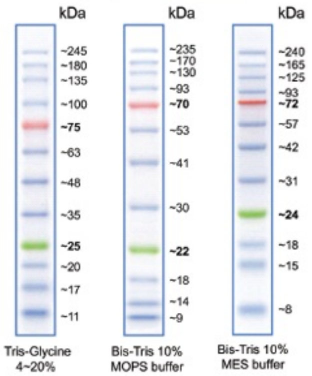

Antibody used :

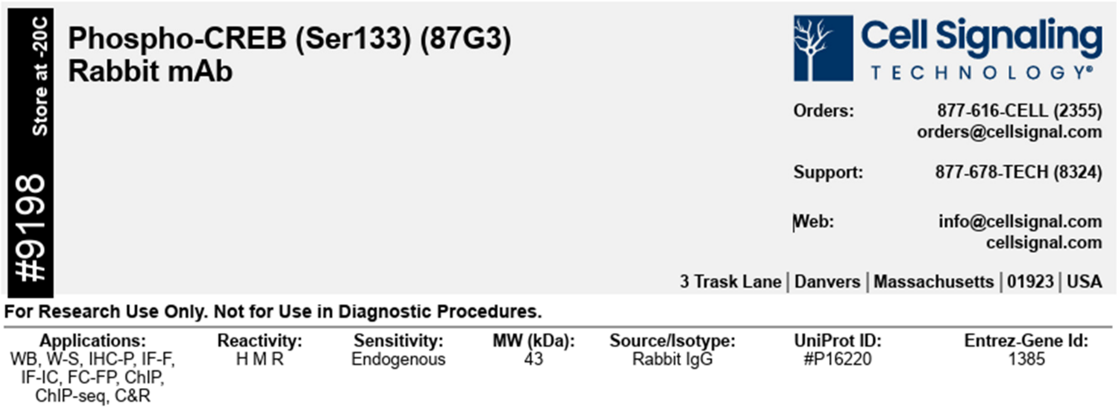

#9198 Store at -20C

Phospho-CREB (Ser133) (87G3) Rabbit mAb

Cell Signaling TECHNOLOGY®

Orders: 877-616-CELL (2355) orders@cellsignal.com

Support: 877-678-TECH (8324)

Web: info@cellsignal.com cellsignal.com

3 Trask Lane | Danvers | Massachusetts | 01923 | USA

For Research Use Only. Not for Use in Diagnostic Procedures.

Applications:	Reactivity:	Sensitivity:	MW (kDa):	Source/Isotype:	UniProt ID:	Entrez-Gene Id:
WB, W-S, IHC-P, IF-F, IF-IC, FC-FP, ChIP, ChIP-seq, C&R	H M R	Endogenous	43	Rabbit IgG	#P16220	1385

1) 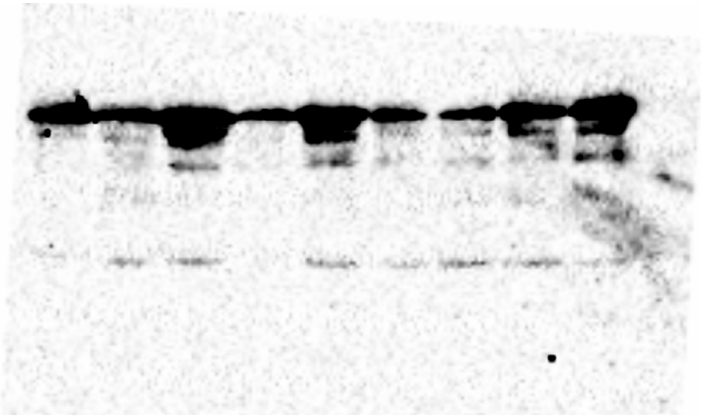

Uncropped image :

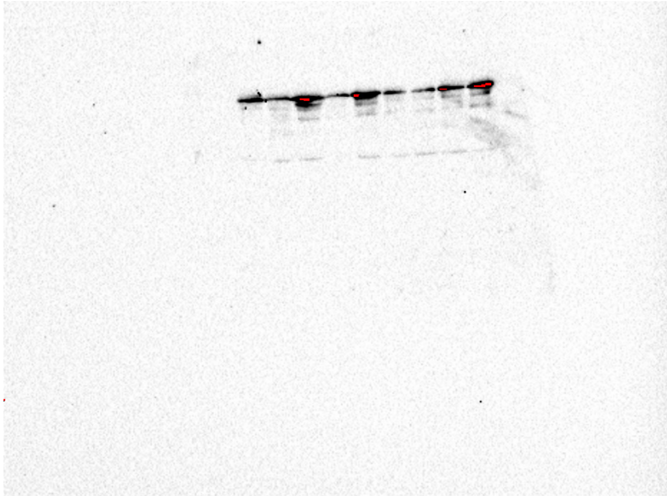

Ladder :

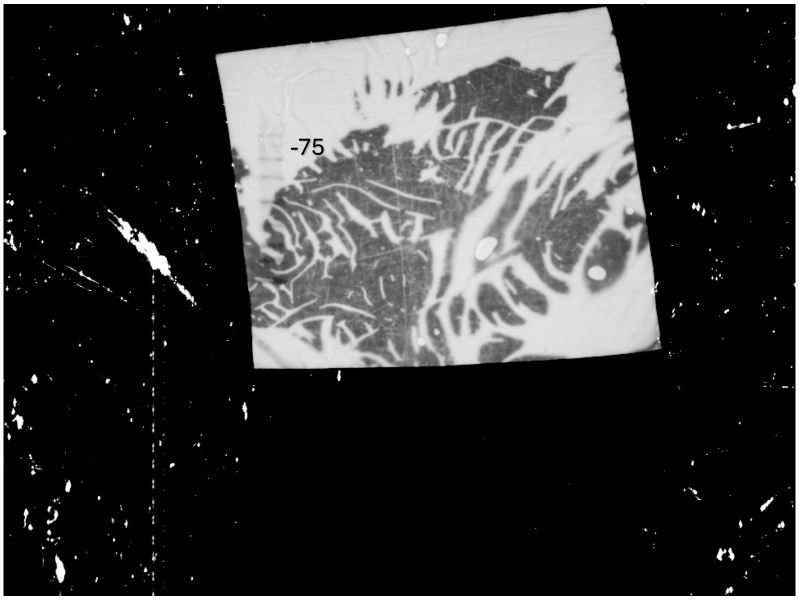

Ladder superimposed :

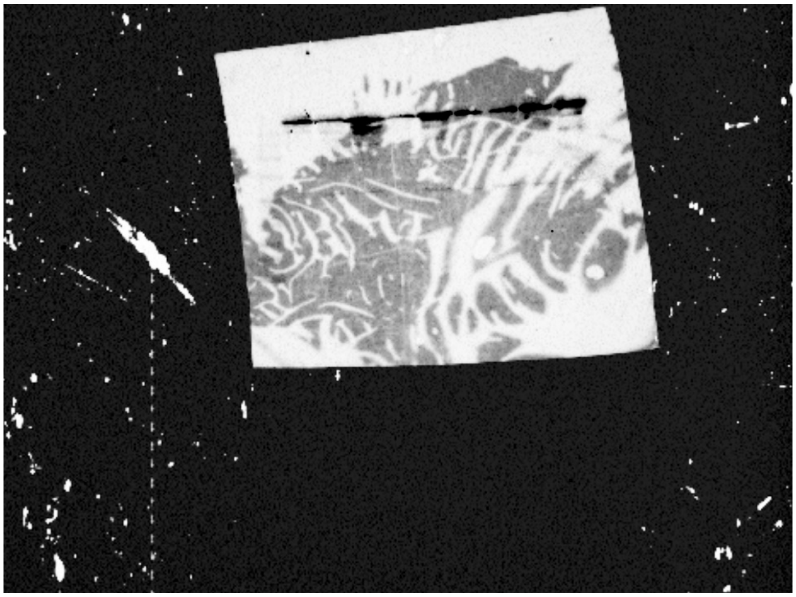

2) 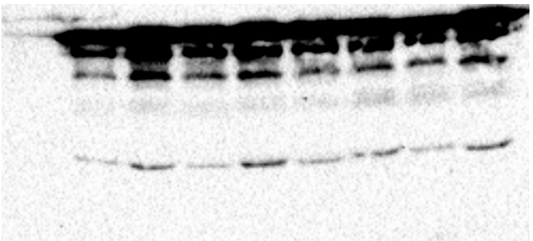

Uncropped image :

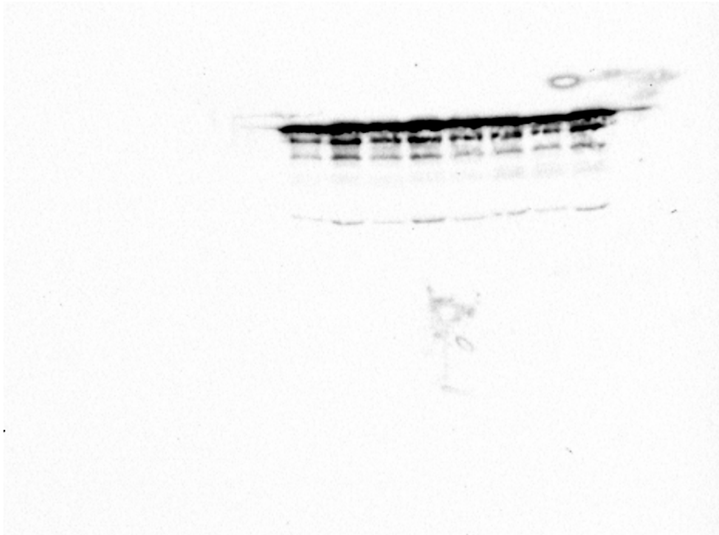

Ladder :

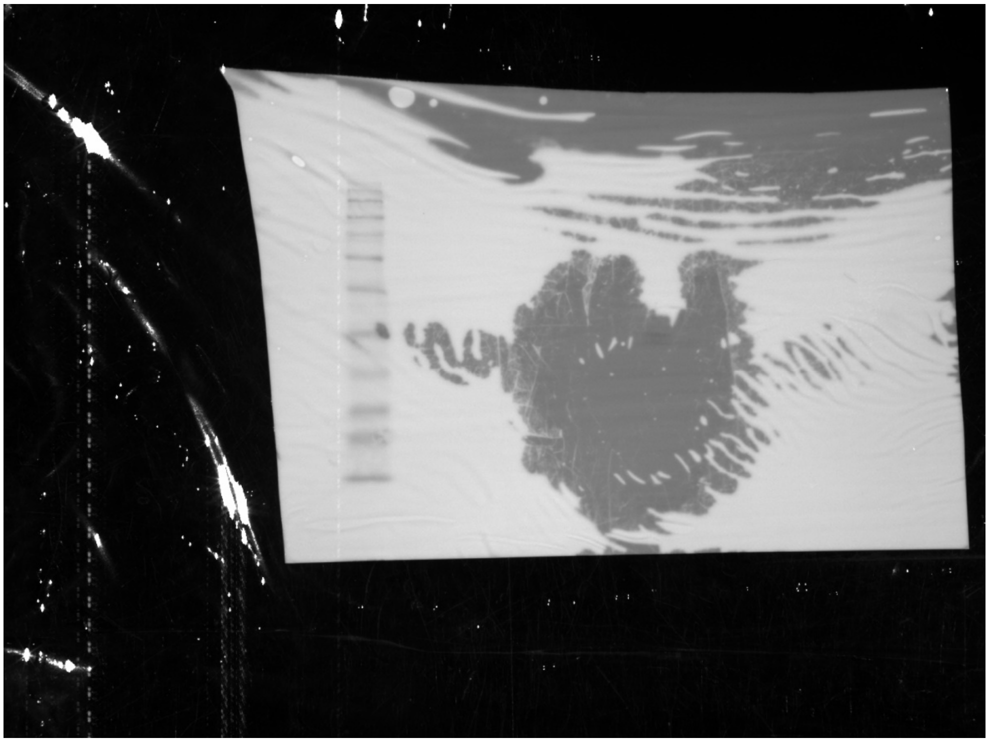

Ladder superimposed :

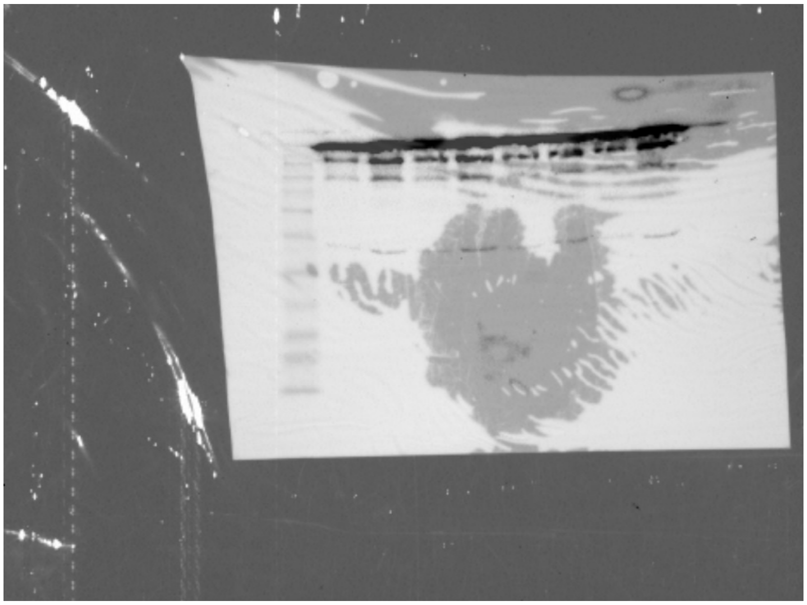

3) 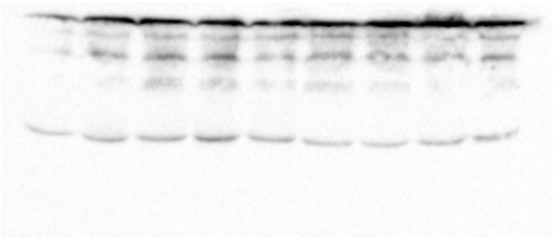

Uncropped image :

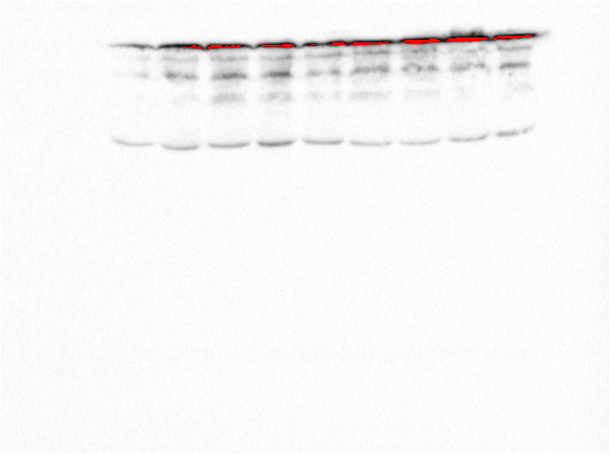

Ladder :

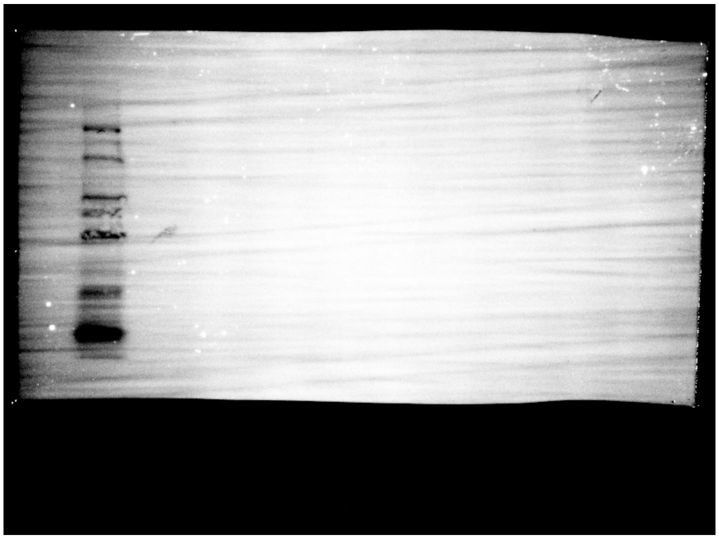

Ladder superimposed :

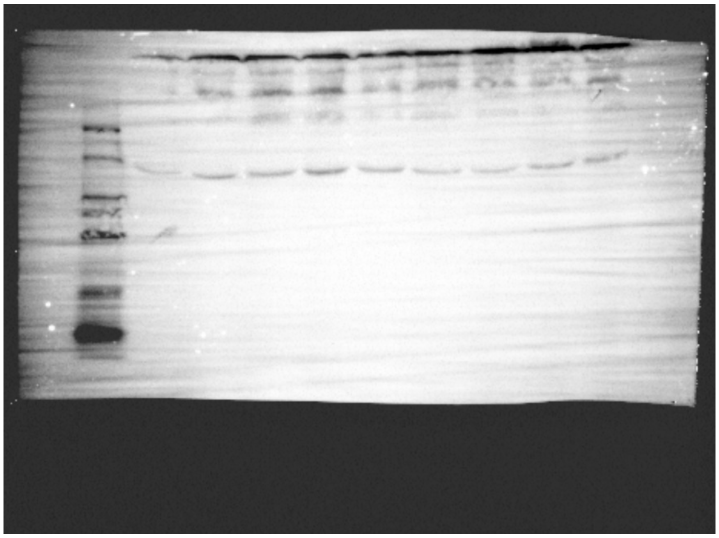

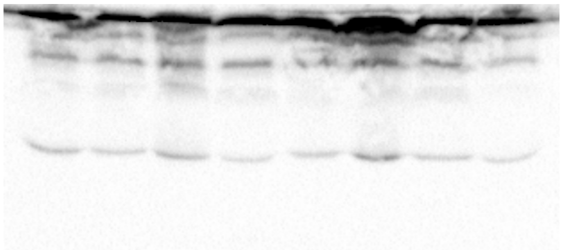

4)

Ladder :

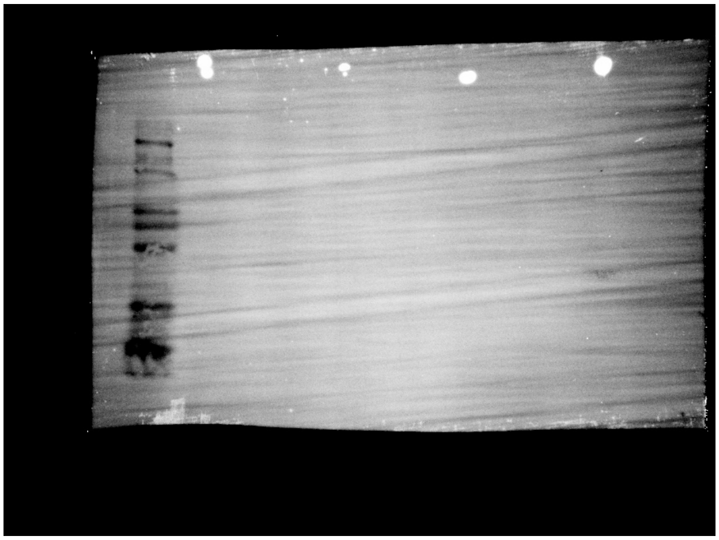

Ladder superimposed :

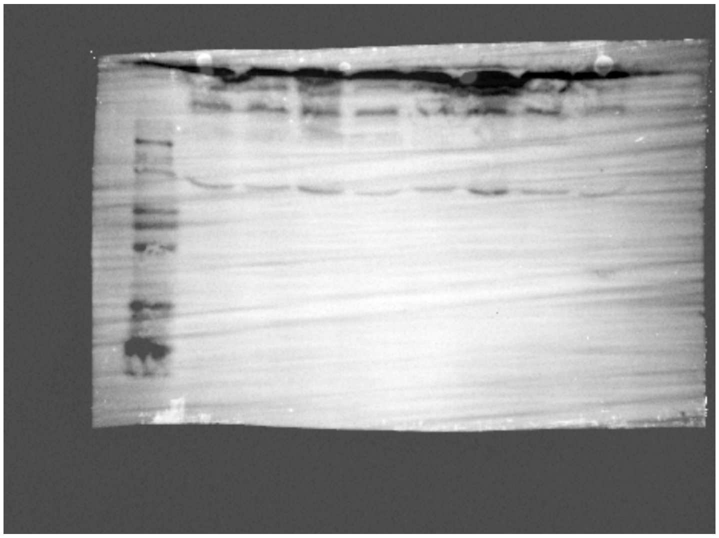

5) 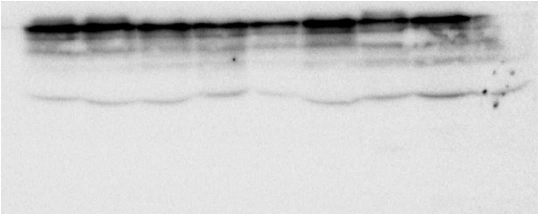

Ladder :

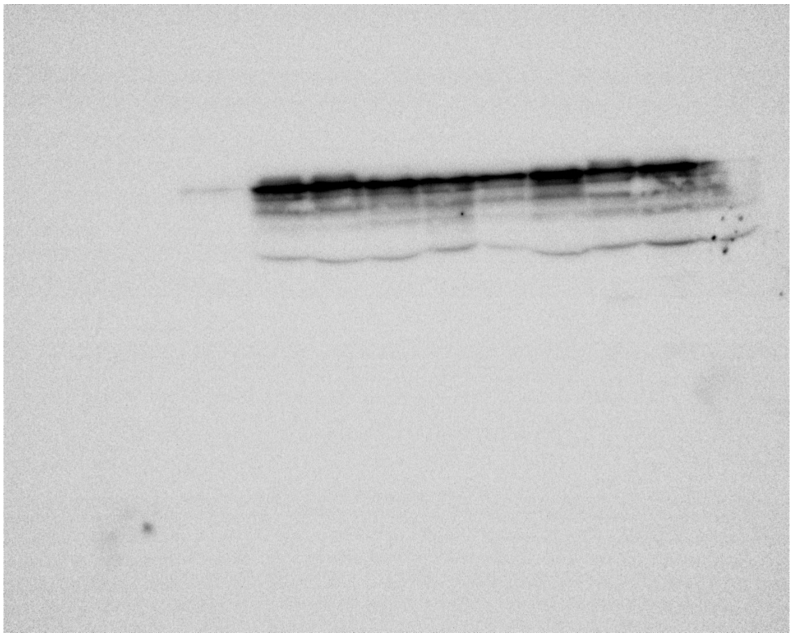

Ladder superimposed :

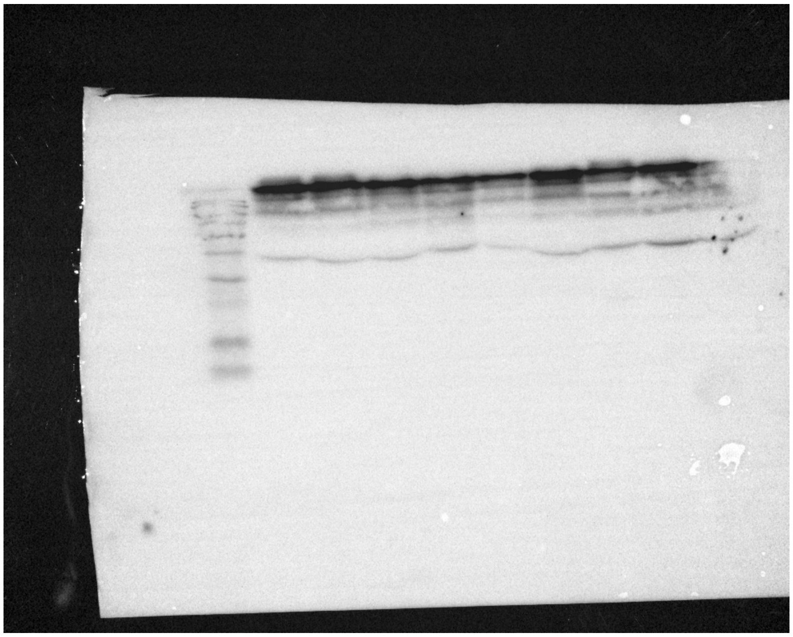

6) 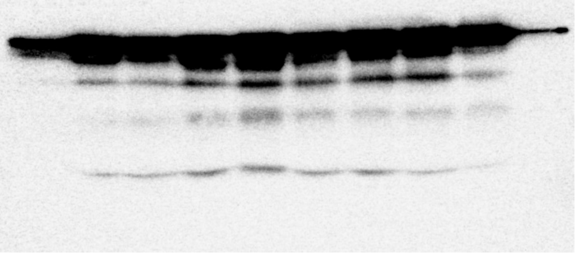

Uncropped image :

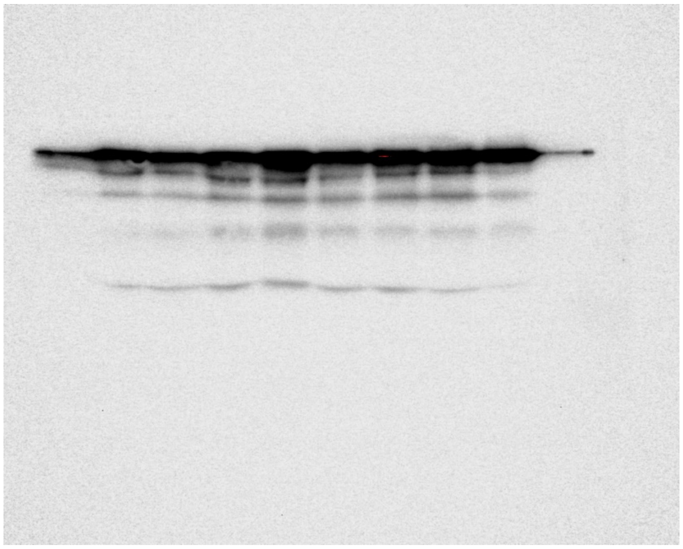

Ladder :

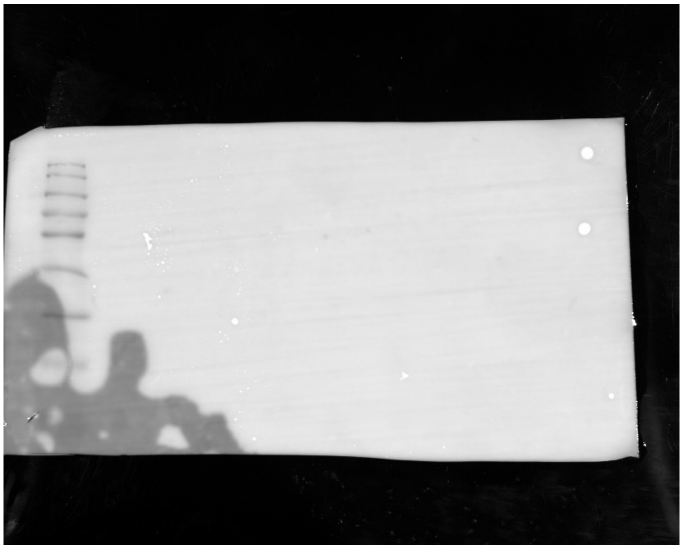

Ladder superimposed :

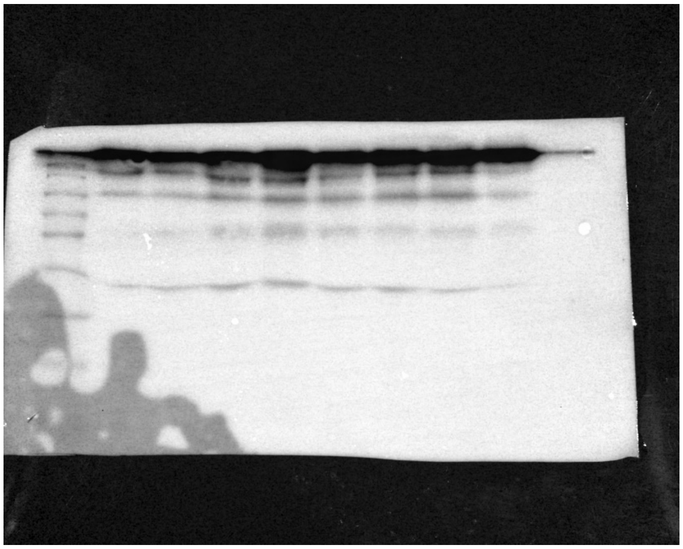

7) 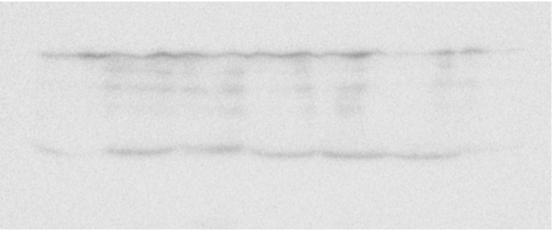

uncropped image :

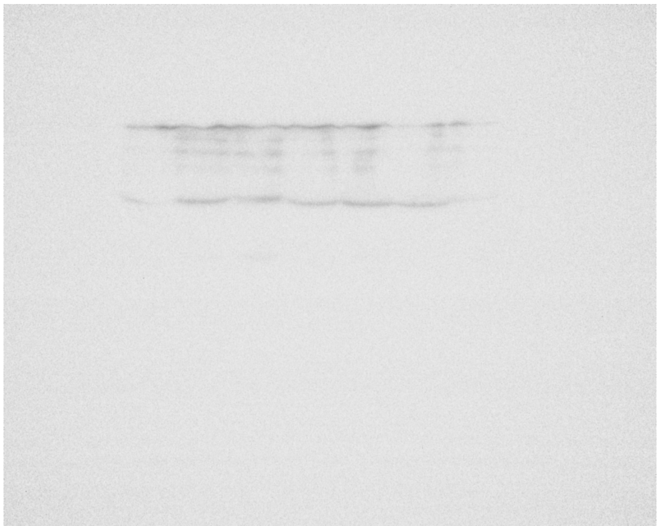

Ladder :

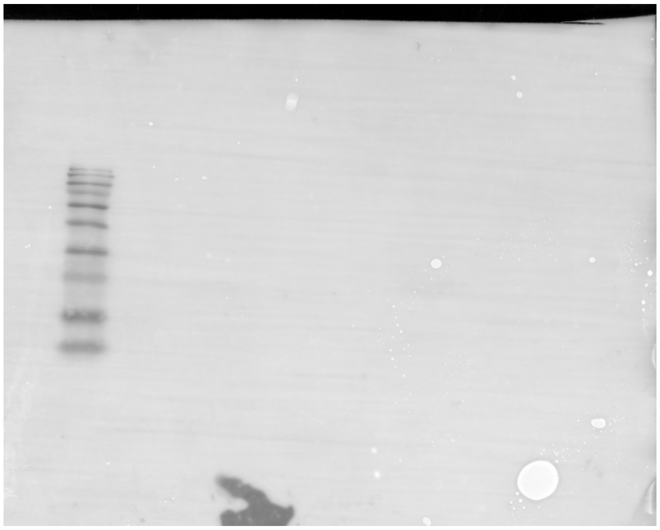

Ladder superimposed :

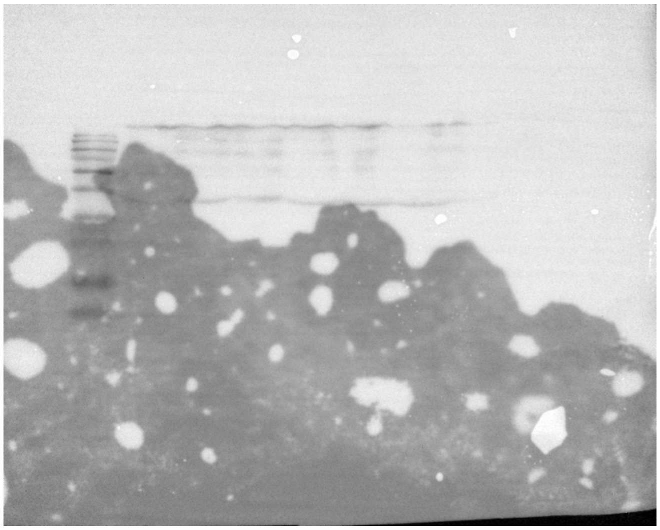

Supplementary Figure S4: Full Western blot images demonstrating expression profile of CREB and GSK3 alpha/beta in Patient Samples vs Pooled healthy patient as control. The sample placement in images are in the same order as specified in the manuscript main figure and are numbered accordingly.

Note: β-Actin was used as the loading control and was detected using two different primary antibodies. One antibody was a rabbit monoclonal anti-β-actin antibody (clone 13E5; Cell Signaling Technology, Cat. No. 4970), while the other was a rabbit polyclonal anti-β-actin antibody (Abgenex, Cat. No. 11-13012). The specifications of both antibodies are provided.

Following the reviewer's comments, the Abgenex anti-β-actin antibody (Cat. No. 11-13012) was additionally used for validation of the β-actin loading control. Accordingly, several membranes were reincubated with this antibody to obtain improved and more consistent β-actin detection. The results obtained using this antibody were used where appropriate to strengthen the assessment of protein loading and to address the reviewer's concerns.

#4970 Store at -20C

beta-Actin (13E5) Rabbit Monoclonal Antibody

Cell Signaling TECHNOLOGY®

Orders: 877-616-CELL (2355) orders@cellsignal.com

Support: 877-678-TECH (8324)

Web: info@cellsignal.com cellsignal.com

3 Trask Lane | Danvers | Massachusetts | 01923 | USA

For Research Use Only. Not for Use in Diagnostic Procedures.

Applications:	Reactivity:	Sensitivity:	MW (kDa):	Source/Isotype:	UniProt ID:	Entrez-Gene Id:
W, W-S, IHC-P, IF-F, IF-IC, FC-FP	H M R Mk B Pg	Endogenous	45	Rabbit IgG	#P60709	60

11-13012: Polyclonal Antibody to Beta actin

**Clonality :**	Polyclonal
**Application :**	WB
**Reactivity :**	Rat,Mouse,Human
**Gene :**	ACTB
**Gene ID :**	60
**Uniprot ID :**	P60709
**Format :**	Purified
**Alternative Name :**	Actin, cytoplasmic 1, ACTB
**Isotype :**	Rabbit IgG

- Supplementary on the loading control along with the main blot image (GSK3 alpha/beta) – membrane 1 to 7

1. 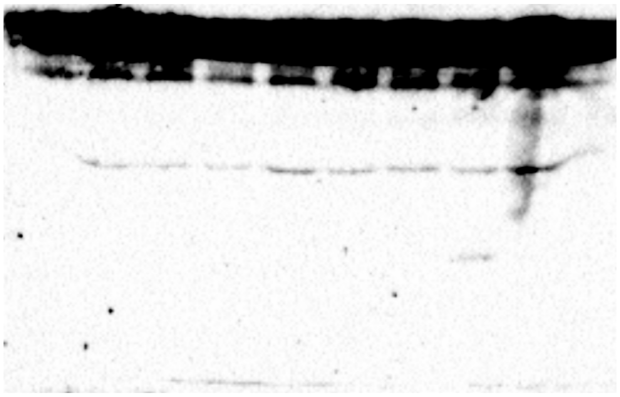

LC – full blot image

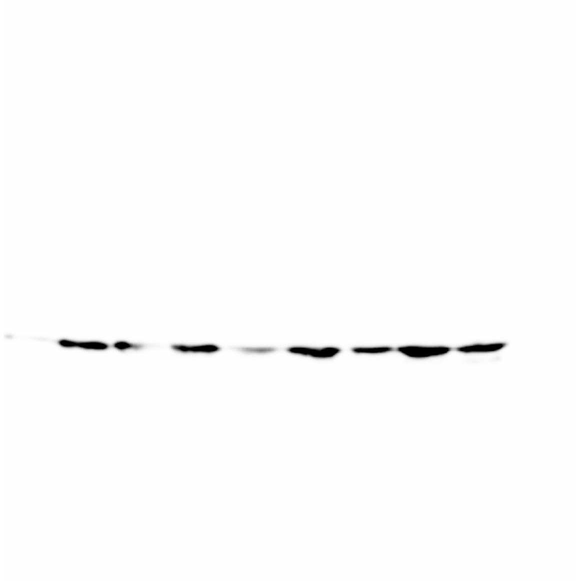

2. 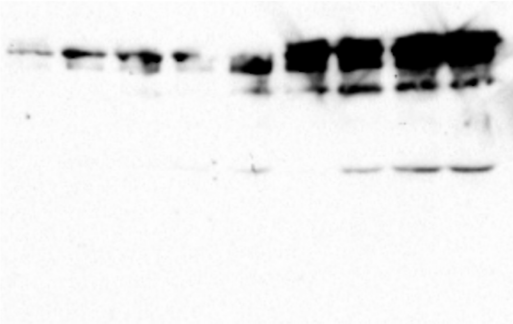

LC – full blot image

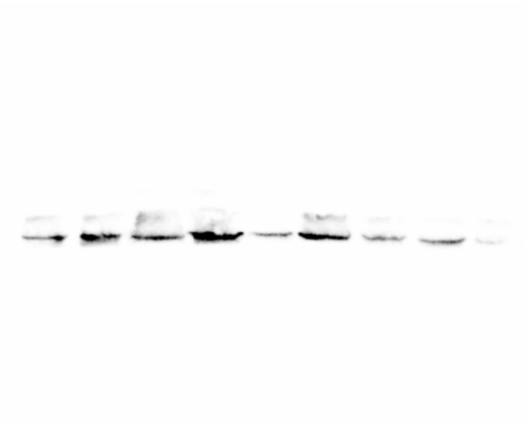

3. 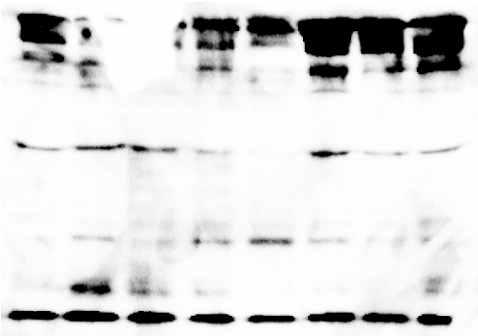

LC – full blot image

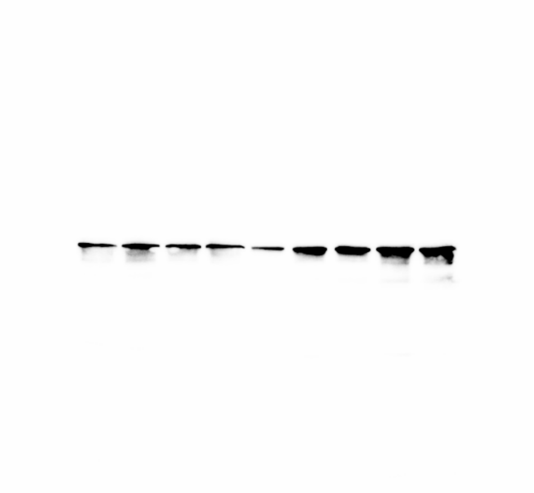

4. 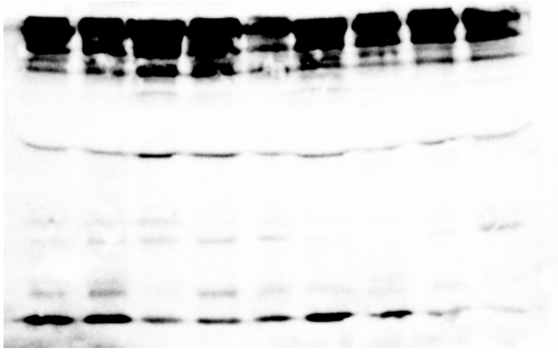

LC – full blot image

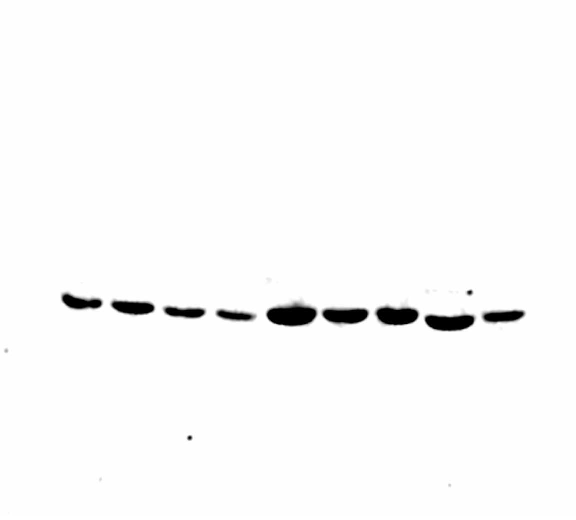

5. 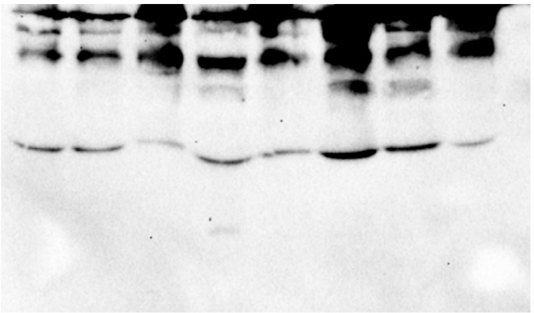

LC – full blot image

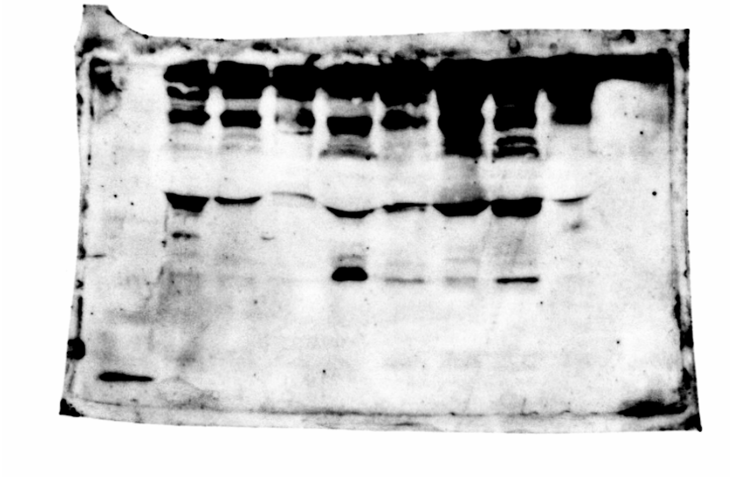

6. 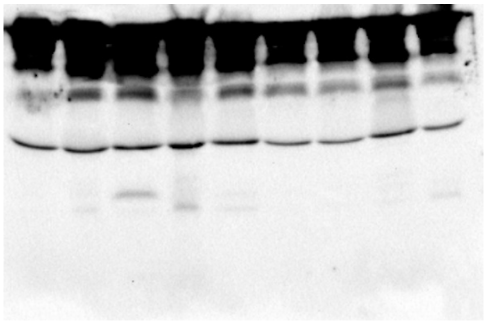

LC – full blot image

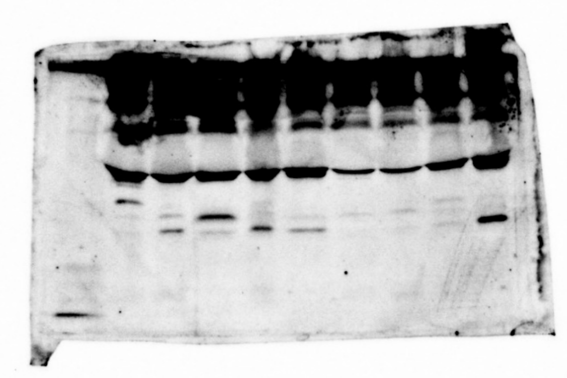

7. 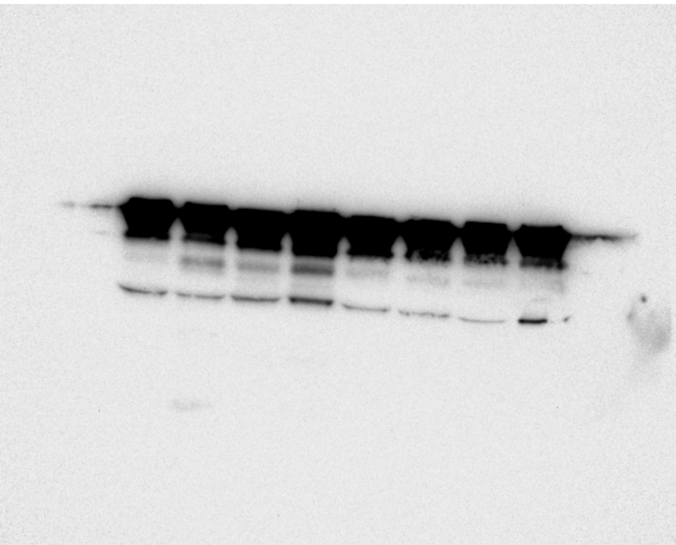

LC – full blot image

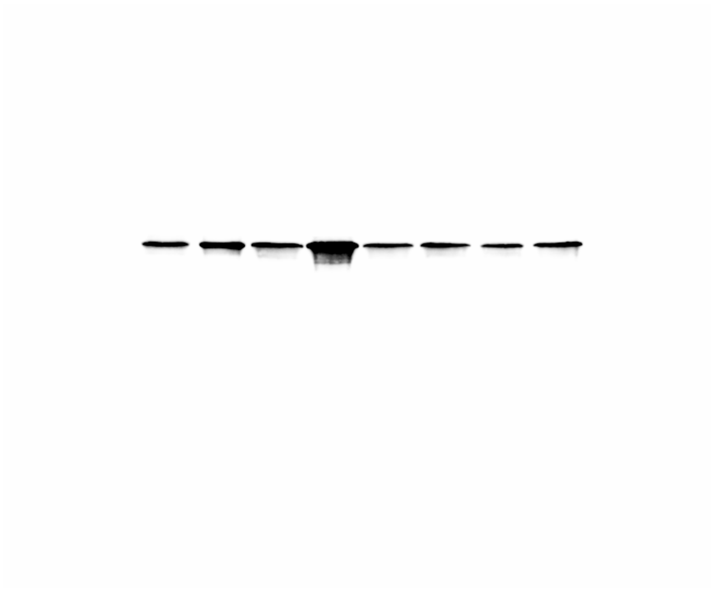

- Supplementary on the loading control along with the main blot image (p – creb1 (ser133)) – membrane 1 to 7

1. 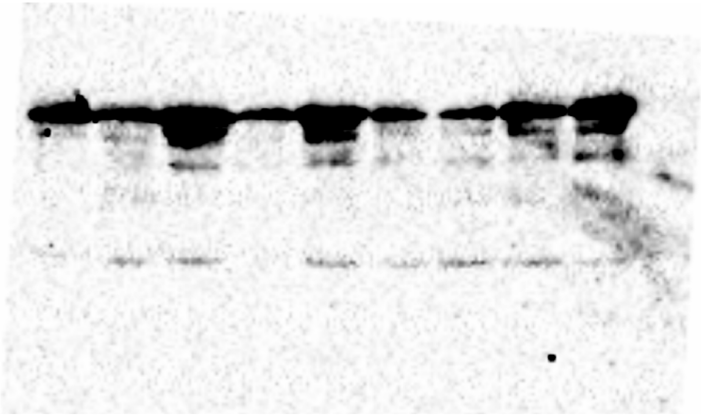

LC – full blot image

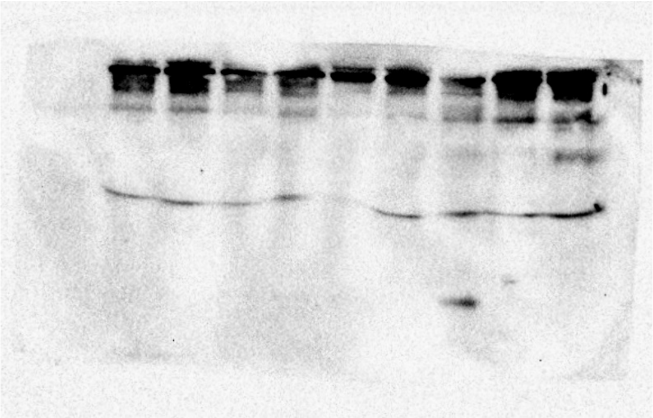

2. 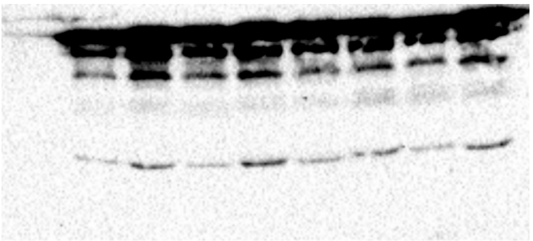

LC – full blot image

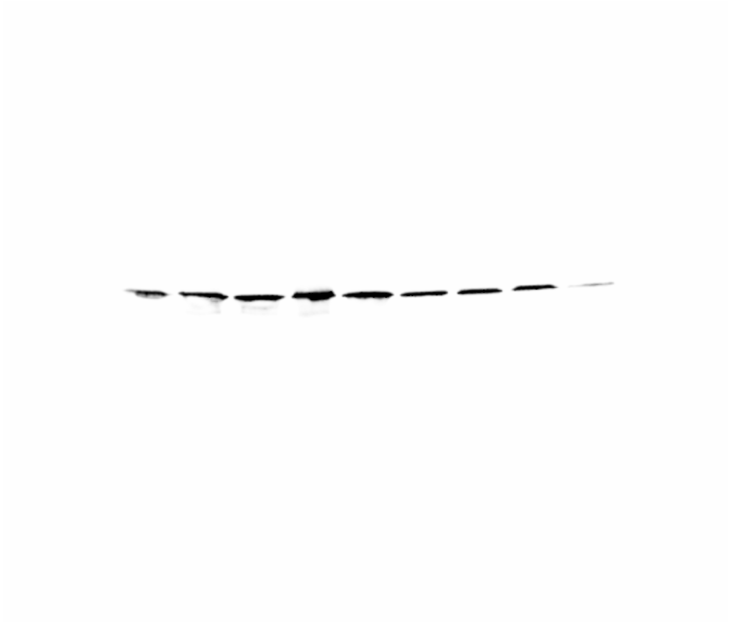

3. 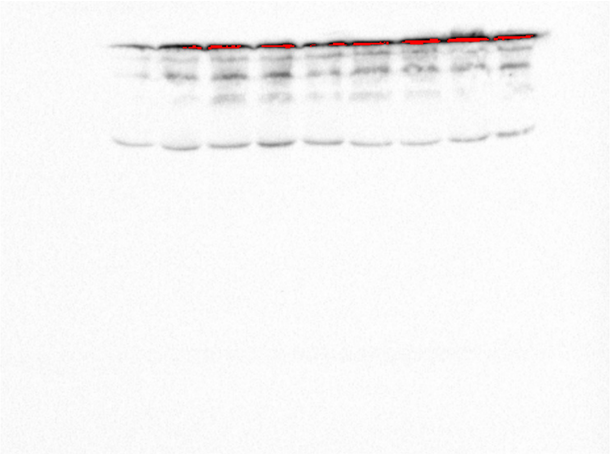

LC – full blot image

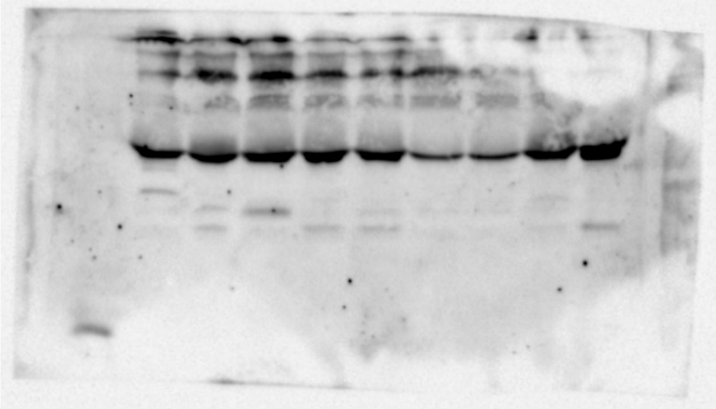

4. 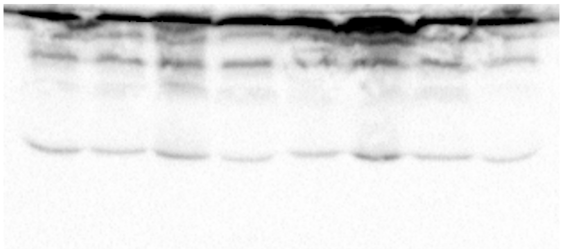

LC – full blot image

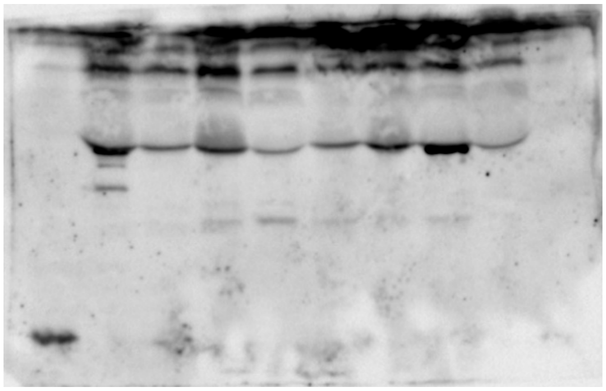

5. 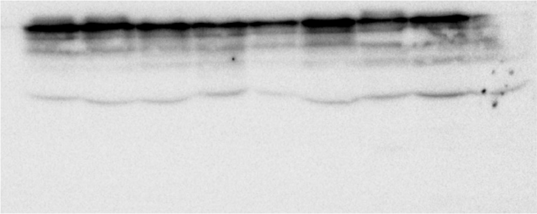

LC – full blot image

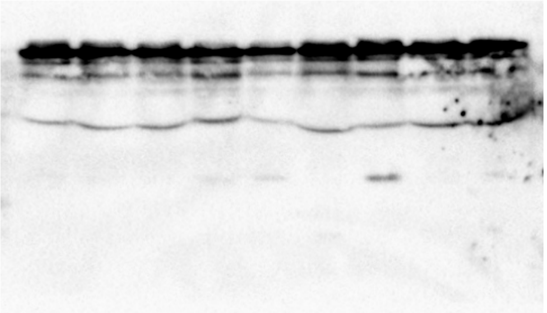

6. 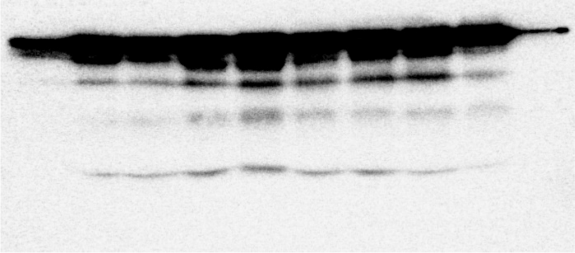

LC – full blot image

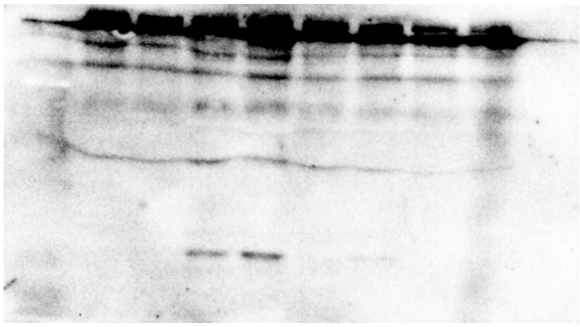

7. 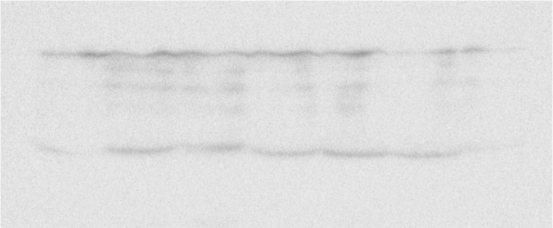

LC – full blot image

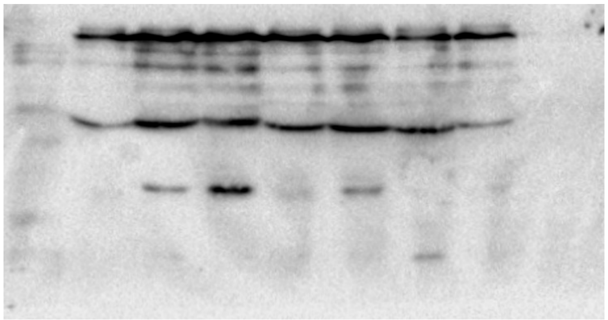

Figure S5. p-GSK3α/β and p- CREB1 (Ser133) expression with β-actin as the loading control. Representative Western blot images showing GSK3α/β and phosphorylated CREB1 (p-CREB1; Ser133) expression, together with the corresponding β-actin loading controls. Blots from membranes 1–7 are shown for each target protein, with the respective β-actin loading-control blots presented alongside the main blot images to demonstrate comparable protein loading and transfer across the membranes.

- Flow chart showing the work through for western blot:

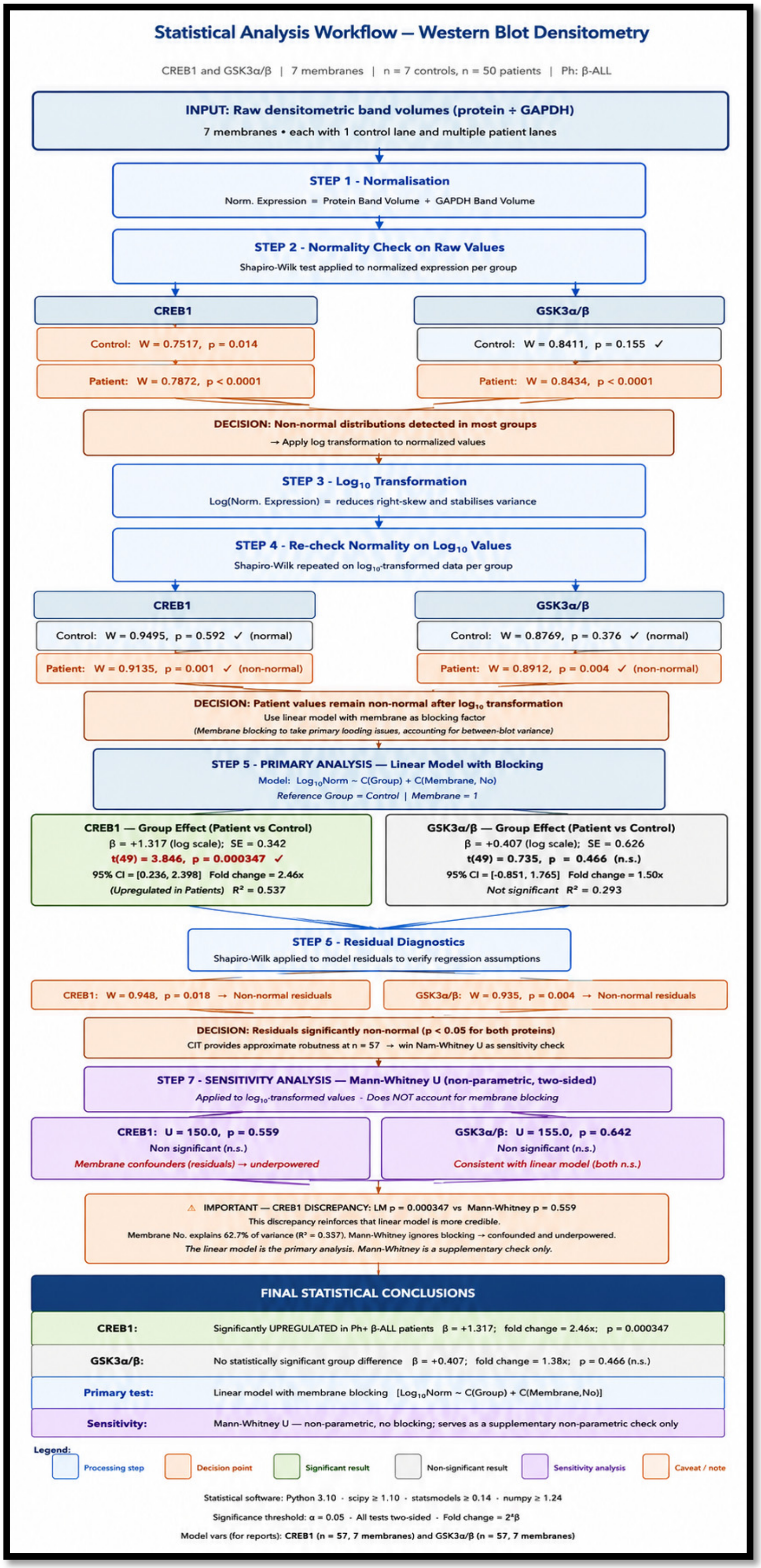
Statistical Analysis Workflow — Western Blot Densitometry
CREB1 and GSK3α/β | 7 membranes | n = 7 controls, n = 50 patients | Ph: β-ALL
INPUT: Raw densitometric band volumes (protein ÷ GAPDH)
7 membranes • each with 1 control lane and multiple patient lanes
STEP 1 - Normalisation
Norm. Expression = Protein Band Volume ÷ GAPDH Band Volume
STEP 2 - Normality Check on Raw Values
Shapiro-Wilk test applied to normalized expression per group
CREB1
Control: W = 0.7517, p = 0.014
Patient: W = 0.7872, p < 0.0001
GSK3α/β
Control: W = 0.8411, p = 0.155 ✓
Patient: W = 0.8434, p < 0.0001
DECISION: Non-normal distributions detected in most groups
→ Apply log transformation to normalized values
STEP 3 - Log10 Transformation
Log(Norm. Expression) = reduces right-skew and stabilises variance
STEP 4 - Re-check Normality on Log10 Values
Shapiro-Wilk repeated on log10-transformed data per group
CREB1
Control: W = 0.9495, p = 0.592 ✓ (normal)
Patient: W = 0.9135, p = 0.001 ✓ (non-normal)
GSK3α/β
Control: W = 0.8769, p = 0.376 ✓ (normal)
Patient: W = 0.8912, p = 0.004 ✓ (non-normal)
DECISION: Patient values remain non-normal after log10 transformation
Use linear model with membrane as blocking factor
(Membrane blocking to take primary looding issues, accounting for between-blot variance)
STEP 5 - PRIMARY ANALYSIS — Linear Model with Blocking
Model: Log10Norm ~ C(Group) + C(Membrane, No)
Reference Group = Control | Membrane = 1
CREB1 — Group Effect (Patient vs Control)
β = +1.317 (log scale); SE = 0.342
t(49) = 3.846, p = 0.000347 ✓
95% CI = [0.236, 2.398] Fold change = 2.46x
(Upregulated in Patients) R² = 0.537
GSK3α/β — Group Effect (Patient vs Control)
β = +0.407 (log scale); SE = 0.626
t(49) = 0.735, p = 0.466 (n.s.)
95% CI = [-0.851, 1.765] Fold change = 1.50x
Not significant R² = 0.293
STEP 6 - Residual Diagnostics
Shapiro-Wilk applied to model residuals to verify regression assumptions
CREB1: W = 0.948, p = 0.018 → Non-normal residuals
GSK3α/β: W = 0.935, p = 0.004 → Non-normal residuals
DECISION: Residuals significantly non-normal (p < 0.05 for both proteins)
CIT provides approximate robutness at n = 57 → win Nam-Whitney U as sensitivity check
STEP 7 - SENSITIVITY ANALYSIS — Mann-Whitney U (non-parametric, two-sided)
Applied to log10-transformed values - Does NOT account for membrane blocking
CREB1: U = 150.0, p = 0.559
Non significant (n.s.)
Membrane confounders (residuals) → underpowered
GSK3α/β: U = 155.0, p = 0.642
Non significant (n.s.)
Consistent with linear model (both n.s.)
⚠ IMPORTANT — CREB1 DISCREPANCY: LM p = 0.000347 vs Mann-Whitney p = 0.559
This discrepancy reinforces that linear model is more credible.
Membrane No. explains 62.7% of variance (R² = 0.357). Mann-Whitney ignores blocking → confounded and underpowered.
The linear model is the primary analysis. Mann-Whitney is a supplementary check only.
FINAL STATISTICAL CONCLUSIONS
CREB1: Significantly UPREGULATED in Ph+ β-ALL patients β = +1.317; fold change = 2.46x; p = 0.000347
GSK3α/β: No statistically significant group difference β = +0.407; fold change = 1.38x; p = 0.466 (n.s.)
Primary test: Linear model with membrane blocking [Log10Norm ~ C(Group) + C(Membrane,No)]
Sensitivity: Mann-Whitney U — non-parametric, no blocking; serves as a supplementary non-parametric check only
Legend:
Processing step
Decision point
Significant result
Non-significant result
Sensitivity analysis
Caveat / note
Statistical software: Python 3.10 · scipy ≥ 1.10 · statsmodels ≥ 0.14 · numpy ≥ 1.24
Significance threshold: α = 0.05 · All tests two-sided · Fold change = 2^β
Model vars (for reports): CREB1 (n = 57, 7 membranes) and GSK3α/β (n = 57, 7 membranes)

Figure S6. Statistical analysis workflow for Western blot densitometry of CREB1 and GSK3α/β. Schematic representation of the statistical analysis workflow applied to densitometric measurements from seven membranes containing control and patient samples from Ph+ B-ALL. Normalized protein expression was assessed for normality using the Shapiro–Wilk test. Because non-normality was observed in most groups, $\log_{10}$ transformation was applied and normality was reassessed. Primary comparisons between patients and controls were performed using a linear model with membrane included as a blocking factor to account for between-membrane variability. The primary analysis showed a significant increase in CREB1 expression in patients compared with controls ($\beta = 1.317$, $t_{49} = 3.846$, $p = 0.000347$; 95% CI: 0.236–2.398; fold change = 2.46; $R^2 = 0.537$), whereas GSK3α/β showed no significant difference between patients and controls ($\beta = 0.407$, $t_{49} = 0.735$, $p = 0.466$; 95% CI: −0.851–1.765; $R^2 = 0.293$). Model residuals were non-normally distributed for both proteins; therefore, two-sided Mann–Whitney U tests were additionally performed as sensitivity analyses. These yielded non-significant differences for CREB1 ($U = 150$, $p = 0.559$) and GSK3α/β ($U = 155$, $p = 0.642$). The workflow identifies the membrane-blocked linear model as the primary analysis and the Mann–Whitney U test as a supplementary sensitivity analysis.

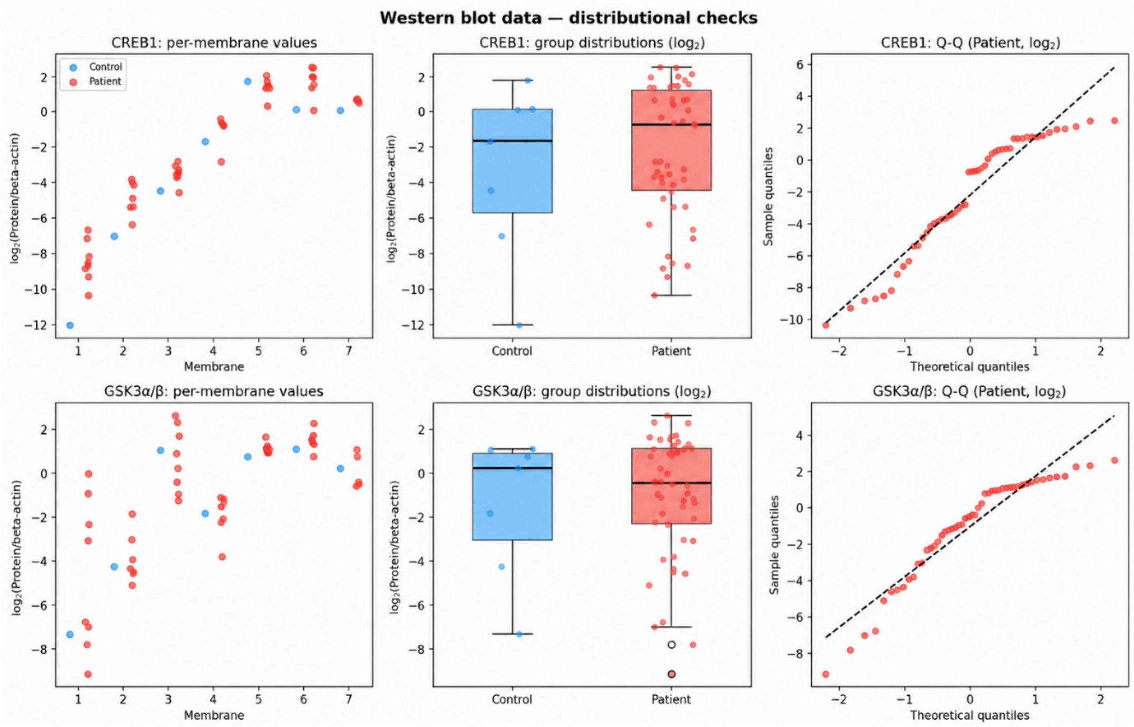

Figure S7 : **Western blot analysis of phosphorylated CREB1 and GSK3α/β across seven membranes.** Western blot protein signals for phosphorylated CREB1 (pCREB1; upper panels) and phosphorylated GSK3α/β (pGSK3α/β; lower panels) were quantified across seven blot membranes. Individual normalized values are shown for each membrane, with Control samples shown in blue and Patient samples in red. Protein signal was normalized to β-actin, and values are presented on a log2 scale as protein/β-actin ratios. Left panels show the individual normalized values by blot membrane (1–7). Middle panels show the distributions of normalized values for the Control and Patient groups using box-and-whisker plots with individual observations overlaid. Right panels show quantile–quantile (Q–Q) plots of the Patient distributions against a theoretical normal distribution. No statistical inference or significance is indicated in this figure.

Supplementary Tables S1-S18

Table S1: Network centrality measures for all proteins in the PPI network.

Protein	Degree	Degree rank (n=52)	Betweenness centrality	Closeness centrality	Highly connected*
**CREB1**	**40**	**16**	**0.019215**	**0.822581**	**Yes**
**GSK3B**	**36**	**21**	**0.006594**	**0.772727**	**Yes**
**GSK3A**	**22**	**37**	**0.001249**	**0.6375**	**No**
JUN	46	1	0.040816	0.910714	Yes
TP53	45	2	0.019157	0.894737	Yes
STAT3	45	3	0.018886	0.894737	Yes
SRC	44	4	0.016434	0.87931	Yes
MYC	44	5	0.019626	0.87931	Yes
ACTB	43	6	0.014458	0.864407	Yes
HSP90AA1	43	7	0.034509	0.864407	Yes
AKT1	42	8	0.026016	0.85	Yes
TNF	42	9	0.031136	0.85	Yes
PTEN	42	10	0.011532	0.85	Yes
CTNNB1	41	11	0.010299	0.836066	Yes
INS	40	12	0.009258	0.822581	Yes
MAPK1	40	13	0.020149	0.822581	Yes
BCL2	40	14	0.009188	0.822581	Yes
MAPK3	40	15	0.013181	0.822581	Yes
EGFR	39	17	0.011078	0.809524	Yes
EGF	39	18	0.011884	0.809524	Yes
KRAS	38	19	0.010955	0.796875	Yes
ESR1	37	20	0.007931	0.784615	Yes
STAT5A	34	22	0.006665	0.75	Yes
STAT5B	33	23	0.003521	0.73913	Yes
AKT2	33	24	0.004238	0.73913	Yes
MAPK14	33	25	0.014584	0.73913	Yes
STAT1	33	26	0.003711	0.73913	Yes
MAPK8	31	27	0.002785	0.71831	Yes
LCK	29	28	0.003626	0.69863	No
LYN	29	29	0.004315	0.69863	No
RPS6KA1	29	30	0.009527	0.69863	No
RPS6KB1	28	31	0.001322	0.689189	No
RPS6KA3	26	32	0.006368	0.662338	No
PDGFRB	25	33	0.001348	0.662338	No
PLCG1	25	34	0.001633	0.662338	No
HSPB1	24	35	0.002572	0.653846	No
STAT6	23	36	0.000794	0.64557	No
MAPK9	22	38	0.000129	0.6375	No

HSPB3	22	39	0.001471	0.6375	No
PTK2B	21	40	0.001751	0.62963	No
NOS3	20	41	0.000257	0.621951	No
AKT1S1	19	42	0.006448	0.614458	No
STAT2	16	43	0.000452	0.586207	No
YES1	16	44	0.000935	0.579545	No
RPS6KA2	16	45	0.002308	0.579545	No
CHEK2	14	46	0	0.579545	No
FGR	11	47	0.000504	0.548387	No
RPS6KA5	11	48	0.000657	0.554348	No
HSPD1	10	49	0.002231	0.548387	No
RPS6KA4	7	50	0.000095	0.51	No
HSPA14	3	51	0	0.485714	No
WNK1	3	52	0.000041	0.5	No

Table S2: KEGG pathway enrichment analysis.

- Raw values post normalization and all the relevant calculations to account for the membrane to membrane differences

Enrichment FDR	nGenes	Pathway Genes	Fold Enrichment	Pathway	URL	Genes
2.47E-26	18	162	62.00813	Path:hsa05161 Hepatitis B	http://www.genome.jp/kegg-bin/show_pathway?hsa05161	CREB1 MAPK14 AKT1 AKT2 PTK2B JUN MAPK1 MAPK3 MAPK8 MAPK9 SRC STAT1 STAT2 STAT3 STAT5A STAT5B STAT6 TP53
3.63E-25	18	194	51.77998	Path:hsa05167 Kaposi sarcoma-associated herpesvirus infection	http://www.genome.jp/kegg-bin/show_pathway?hsa05167	CREB1 MAPK14 CTNNB1 AKT1 AKT2 GSK3B JUN LYN PLCG1 MAPK1 MAPK3 MAPK8 MAPK9 SRC STAT1 STAT2 STAT3 TP53
5.21E-23	14	84	93.0122	Path:hsa04012 ErbB signaling pathway	http://www.genome.jp/kegg-bin/show_pathway?hsa04012	EGF AKT1 AKT2 GSK3B JUN PLCG1 MAPK1 MAPK3 MAPK8 MAPK9 RPS6KB1 SRC STAT5A STAT5B
2.52E-22	21	530	22.11233	Path:hsa05200 Pathways in cancer	http://www.genome.jp/kegg-bin/show_pathway?hsa05200	CTNNB1 EGF AKT1 AKT2 GSK3B JUN PDGFRB PLCG1 MAPK1 MAPK3 MAPK8 MAPK9 RPS6KB1 STAT1 STAT2 STAT3 STAT5A STAT5B STAT6 TP53 RPS6KA5
3.06E-22	13	70	103.6422	Path:hsa04917 Prolactin signaling pathway	http://www.genome.jp/kegg-bin/show_pathway?hsa04917	MAPK14 AKT1 AKT2 GSK3B MAPK1 MAPK3 MAPK8 MAPK9 SRC STAT1 STAT3 STAT5A STAT5B
3.06E-22	14	100	78.13024	Path:hsa04933 AGE-RAGE signaling pathway in diabetic complications	http://www.genome.jp/kegg-bin/show_pathway?hsa04933	MAPK14 AKT1 AKT2 JUN NOS3 PLCG1 MAPK1 MAPK3 MAPK8 MAPK9 STAT1 STAT3 STAT5A STAT5B
3.06E-22	15	137	61.1029	Path:hsa05135 Yersinia infection	http://www.genome.jp/kegg-bin/show_pathway?hsa05135	MAPK14 AKT1 AKT2 PTK2B GSK3B JUN LCK PLCG1 MAPK1 MAPK3 MAPK8 MAPK9 RPS6KA2 RPS6KA3 SRC
3.17E-21	14	119	65.65567	Path:hsa04722 Neurotrophin signaling pathway	http://www.genome.jp/kegg-bin/show_pathway?hsa04722	MAPK14 AKT1 AKT2 GSK3B JUN PLCG1 MAPK1 MAPK3 MAPK8 MAPK9 RPS6KA2 RPS6KA3 TP53 RPS6KA5
3.17E-21	14	120	65.10854	Path:hsa04935 Growth hormone synthesis secretion and action	http://www.genome.jp/kegg-bin/show_pathway?hsa04935	CREB1 MAPK14 AKT1 AKT2 GSK3B PLCG1 MAPK1 MAPK3 MAPK8 MAPK9 STAT1 STAT3 STAT5A STAT5B
3.17E-21	16	214	41.7251	Path:hsa05417 Lipid and atherosclerosis	http://www.genome.jp/kegg-bin/show_pathway?hsa05417	MAPK14 AKT1 AKT2 GSK3B HSPD1 JUN LYN NOS3 PLCG1 MAPK1 MAPK3 MAPK8 MAPK9 SRC STAT3 TP53
2.67E-20	16	246	36.29744	Path:hsa05131 Shigellosis	http://www.genome.jp/kegg-bin/show_pathway?hsa05131	MAPK14 AKT1 AKT2 GSK3A GSK3B JUN PLCG1 MAPK1 MAPK3 MAPK8 MAPK9 RPS6KB1 SRC TP53 AKT1S1 RPS6KA5
3.10E-20	15	191	43.82774	Path:hsa04062 Chemokine signaling pathway	http://www.genome.jp/kegg-bin/show_pathway?hsa04062	AKT1 AKT2 PTK2B FGR GSK3A GSK3B LYN PLCG1 MAPK1 MAPK3 SRC STAT1 STAT2 STAT3 STAT5B

6.18E-20	13	108	67.17547	Path:hsa04659 Th17 cell differentiation	http://www.genome.jp/kegg-bin/show_pathway?hsa04659	MAPK14 JUN LCK PLCG1 MAPK1 MAPK3 MAPK8 MAPK9 STAT1 STAT3 STAT5A STAT5B STAT6
3.43E-19	12	86	77.87067	Path:hsa05210 Colorectal cancer	http://www.genome.jp/kegg-bin/show_pathway?hsa05210	CTNNB1 EGF AKT1 AKT2 GSK3B JUN MAPK1 MAPK3 MAPK8 MAPK9 RPS6KB1 TP53
3.51E-19	16	294	30.37133	Path:hsa04010 MAPK signaling pathway	http://www.genome.jp/kegg-bin/show_pathway?hsa04010	MAPK14 EGF AKT1 AKT2 HSPB1 JUN PDGFRB MAPK1 MAPK3 MAPK8 MAPK9 RPS6KA2 RPS6KA3 TP53 RPS6KA4 RPS6KA5
4.65E-19	12	89	75.24582	Path:hsa05235 PD-L1 expression and PD-1 checkpoint pathway in cancer	http://www.genome.jp/kegg-bin/show_pathway?hsa05235	MAPK14 EGF AKT1 AKT2 JUN LCK PLCG1 MAPK1 MAPK3 RPS6KB1 STAT1 STAT3
6.66E-19	12	92	72.79215	Path:hsa04658 Th1 and Th2 cell differentiation	http://www.genome.jp/kegg-bin/show_pathway?hsa04658	MAPK14 JUN LCK PLCG1 MAPK1 MAPK3 MAPK8 MAPK9 STAT1 STAT5A STAT5B STAT6
2.29E-18	14	197	39.66002	Path:hsa05207 Chemical carcinogenesis-receptor activation	http://www.genome.jp/kegg-bin/show_pathway?hsa05207	CREB1 EGF AKT1 AKT2 JUN MAPK1 MAPK3 RPS6KA2 RPS6KA3 RPS6KB1 SRC STAT3 STAT5A STAT5B
7.28E-18	11	76	80.77375	Path:hsa05212 Pancreatic cancer	http://www.genome.jp/kegg-bin/show_pathway?hsa05212	EGF AKT1 AKT2 MAPK1 MAPK3 MAPK8 MAPK9 RPS6KB1 STAT1 STAT3 TP53
1.09E-17	11	79	77.70639	Path:hsa01521 EGFR tyrosine kinase inhibitor resistance	http://www.genome.jp/kegg-bin/show_pathway?hsa01521	EGF AKT1 AKT2 GSK3B PDGFRB PLCG1 MAPK1 MAPK3 RPS6KB1 SRC STAT3
2.78E-17	12	126	53.14983	Path:hsa04380 Osteoclast differentiation	http://www.genome.jp/kegg-bin/show_pathway?hsa04380	CREB1 MAPK14 AKT1 AKT2 JUN LCK MAPK1 MAPK3 MAPK8 MAPK9 STAT1 STAT2
8.38E-17	11	95	64.619	Path:hsa01522 Endocrine resistance	http://www.genome.jp/kegg-bin/show_pathway?hsa01522	MAPK14 AKT1 AKT2 JUN MAPK1 MAPK3 MAPK8 MAPK9 RPS6KB1 SRC TP53
8.55E-17	12	139	48.17898	Path:hsa05162 Measles	http://www.genome.jp/kegg-bin/show_pathway?hsa05162	AKT1 AKT2 GSK3B JUN MAPK8 MAPK9 STAT1 STAT2 STAT3 STAT5A STAT5B TP53
1.10E-16	11	98	62.64087	Path:hsa05231 Choline metabolism in cancer	http://www.genome.jp/kegg-bin/show_pathway?hsa05231	EGF AKT1 AKT2 JUN PDGFRB PLCG1 MAPK1 MAPK3 MAPK8 MAPK9 RPS6KB1
1.87E-16	11	103	59.60005	Path:hsa04660 T cell receptor signaling pathway	http://www.genome.jp/kegg-bin/show_pathway?hsa04660	MAPK14 AKT1 AKT2 GSK3B JUN LCK PLCG1 MAPK1 MAPK3 MAPK8 MAPK9
2.00E-16	11	104	59.02697	Path:hsa04625 C-type lectin receptor signaling pathway	http://www.genome.jp/kegg-bin/show_pathway?hsa04625	MAPK14 AKT1 AKT2 JUN MAPK1 MAPK3 MAPK8 MAPK9 SRC STAT1 STAT2
2.97E-16	11	108	56.84079	Path:hsa04931 Insulin resistance	http://www.genome.jp/kegg-bin/show_pathway?hsa04931	CREB1 AKT1 AKT2 GSK3B NOS3 MAPK8 MAPK9 RPS6KA2 RPS6KA3 RPS6KB1 STAT3
3.42E-16	10	72	77.51016	Path:hsa05223 Non-small cell lung cancer	http://www.genome.jp/kegg-bin/show_pathway?hsa05223	EGF AKT1 AKT2 PLCG1 MAPK1 MAPK3 STAT3 STAT5A STAT5B TP53
4.07E-16	11	112	54.81076	Path:hsa04668 TNF signaling pathway	http://www.genome.jp/kegg-bin/show_pathway?hsa04668	CREB1 MAPK14 AKT1 AKT2 JUN MAPK1 MAPK3 MAPK8 MAPK9 RPS6KA4 RPS6KA5

4.07E-16	13	222	32.67996	Path:hsa05166 Human T-cell leukemia virus 1 infection	http://www.genome.jp/kegg-bin/show_pathway?hsa05166	CHEK2 CREB1 AKT1 AKT2 JUN LCK MAPK1 MAPK3 MAPK8 MAPK9 STAT5A STAT5B TP53
4.44E-16	13	224	32.38818	Path:hsa05163 Human cytomegalovirus infection	http://www.genome.jp/kegg-bin/show_pathway?hsa05163	CREB1 MAPK14 CTNNB1 AKT1 AKT2 PTK2B GSK3B MAPK1 MAPK3 RPS6KB1 SRC STAT3 TP53
1.88E-15	11	129	47.58763	Path:hsa04926 Relaxin signaling pathway	http://www.genome.jp/kegg-bin/show_pathway?hsa04926	CREB1 MAPK14 AKT1 AKT2 JUN NOS3 MAPK1 MAPK3 MAPK8 MAPK9 SRC
4.93E-15	9	59	85.12981	Path:hsa04370 VEGF signaling pathway	http://www.genome.jp/kegg-bin/show_pathway?hsa04370	MAPK14 AKT1 AKT2 HSPB1 NOS3 PLCG1 MAPK1 MAPK3 SRC
4.94E-15	12	200	33.48439	Path:hsa04510 Focal adhesion	http://www.genome.jp/kegg-bin/show_pathway?hsa04510	CTNNB1 EGF AKT1 AKT2 GSK3B JUN PDGFRB MAPK1 MAPK3 MAPK8 MAPK9 SRC
6.22E-15	10	97	57.53332	Path:hsa05215 Prostate cancer	http://www.genome.jp/kegg-bin/show_pathway?hsa05215	CREB1 CTNNB1 EGF AKT1 AKT2 GSK3B PDGFRB MAPK1 MAPK3 TP53
1.52E-14	11	157	39.10067	Path:hsa05160 Hepatitis C	http://www.genome.jp/kegg-bin/show_pathway?hsa05160	CTNNB1 EGF AKT1 AKT2 GSK3B MAPK1 MAPK3 STAT1 STAT2 STAT3 TP53
1.70E-14	9	68	73.86263	Path:hsa04664 Fc epsilon RI signaling pathway	http://www.genome.jp/kegg-bin/show_pathway?hsa04664	MAPK14 AKT1 AKT2 LYN PLCG1 MAPK1 MAPK3 MAPK8 MAPK9
5.60E-14	10	121	46.12175	Path:hsa04919 Thyroid hormone signaling pathway	http://www.genome.jp/kegg-bin/show_pathway?hsa04919	CTNNB1 AKT1 AKT2 GSK3B PLCG1 MAPK1 MAPK3 SRC STAT1 TP53
5.61E-14	13	331	21.91828	Path:hsa05165 Human papillomavirus infection	http://www.genome.jp/kegg-bin/show_pathway?hsa05165	CREB1 CTNNB1 EGF AKT1 AKT2 GSK3B PDGFRB MAPK1 MAPK3 RPS6KB1 STAT1 STAT2 TP53
5.90E-14	11	179	34.295	Path:hsa05152 Tuberculosis	http://www.genome.jp/kegg-bin/show_pathway?hsa05152	CREB1 MAPK14 AKT1 AKT2 HSPD1 MAPK1 MAPK3 MAPK8 MAPK9 SRC STAT1
1.98E-13	10	138	40.44008	Path:hsa05418 Fluid shear stress and atherosclerosis	http://www.genome.jp/kegg-bin/show_pathway?hsa05418	MAPK14 CTNNB1 AKT1 AKT2 JUN NOS3 MAPK8 MAPK9 SRC TP53
2.09E-13	11	202	30.39012	Path:hsa05169 Epstein-Barr virus infection	http://www.genome.jp/kegg-bin/show_pathway?hsa05169	MAPK14 AKT1 AKT2 JUN LYN MAPK8 MAPK9 STAT1 STAT2 STAT3 TP53
2.09E-13	11	202	30.39012	Path:hsa05205 Proteoglycans in cancer	http://www.genome.jp/kegg-bin/show_pathway?hsa05205	MAPK14 CTNNB1 AKT1 AKT2 PLCG1 MAPK1 MAPK3 RPS6KB1 SRC STAT3 TP53
3.14E-13	11	210	29.2324	Path:hsa05170 Human immunodeficiency virus 1 infection	http://www.genome.jp/kegg-bin/show_pathway?hsa05170	MAPK14 AKT1 AKT2 PTK2B JUN PLCG1 MAPK1 MAPK3 MAPK8 MAPK9 RPS6KB1
3.43E-13	10	147	37.96416	Path:hsa05224 Breast cancer	http://www.genome.jp/kegg-bin/show_pathway?hsa05224	CTNNB1 EGF AKT1 AKT2 GSK3B JUN MAPK1 MAPK3 RPS6KB1 TP53
3.73E-13	8	58	76.97561	Path:hsa05213 Endometrial cancer	http://www.genome.jp/kegg-bin/show_pathway?hsa05213	CTNNB1 EGF AKT1 AKT2 GSK3B MAPK1 MAPK3 TP53

5.97E-13	9	102	49.24175	Path:hsa04914 Progesterone-mediated oocyte maturation	http://www.genome.jp/kegg-bin/show_pathway?hsa04914	MAPK14 AKT1 AKT2 MAPK1 MAPK3 MAPK8 MAPK9 RPS6KA2 RPS6KA3
6.40E-13	9	103	48.76368	Path:hsa04620 Toll-like receptor signaling pathway	http://www.genome.jp/kegg-bin/show_pathway?hsa04620	MAPK14 AKT1 AKT2 JUN MAPK1 MAPK3 MAPK8 MAPK9 STAT1
8.43E-13	10	162	34.44896	Path:hsa04630 JAK-STAT signaling pathway	http://www.genome.jp/kegg-bin/show_pathway?hsa04630	EGF AKT1 AKT2 PDGFRB STAT1 STAT2 STAT3 STAT5A STAT5B STAT6
1.04E-12	9	109	46.07944	Path:hsa04066 HIF-1 signaling pathway	http://www.genome.jp/kegg-bin/show_pathway?hsa04066	EGF AKT1 AKT2 NOS3 PLCG1 MAPK1 MAPK3 RPS6KB1 STAT3
1.13E-12	8	67	66.6356	Path:hsa05221 Acute myeloid leukemia	http://www.genome.jp/kegg-bin/show_pathway?hsa05221	AKT1 AKT2 MAPK1 MAPK3 RPS6KB1 STAT3 STAT5A STAT5B
1.28E-12	9	112	44.84517	Path:hsa05145 Toxoplasmosis	http://www.genome.jp/kegg-bin/show_pathway?hsa05145	MAPK14 AKT1 AKT2 MAPK1 MAPK3 MAPK8 MAPK9 STAT1 STAT3
2.19E-12	9	119	42.20721	Path:hsa04071 Sphingolipid signaling pathway	http://www.genome.jp/kegg-bin/show_pathway?hsa04071	MAPK14 AKT1 AKT2 NOS3 MAPK1 MAPK3 MAPK8 MAPK9 TP53
2.74E-12	8	75	59.5278	Path:hsa05214 Glioma	http://www.genome.jp/kegg-bin/show_pathway?hsa05214	EGF AKT1 AKT2 PDGFRB PLCG1 MAPK1 MAPK3 TP53
5.08E-12	9	131	38.3409	Path:hsa04068 FoxO signaling pathway	http://www.genome.jp/kegg-bin/show_pathway?hsa04068	MAPK14 EGF AKT1 AKT2 MAPK1 MAPK3 MAPK8 MAPK9 STAT3
6.75E-12	10	202	27.62738	Path:hsa05203 Viral carcinogenesis	http://www.genome.jp/kegg-bin/show_pathway?hsa05203	CREB1 JUN LYN MAPK1 MAPK3 SRC STAT3 STAT5A STAT5B TP53
9.76E-12	10	210	26.57491	Path:hsa04015 Rap1 signaling pathway	http://www.genome.jp/kegg-bin/show_pathway?hsa04015	MAPK14 CTNNB1 EGF AKT1 AKT2 PDGFRB PLCG1 MAPK1 MAPK3 SRC
1.46E-11	9	148	33.93688	Path:hsa05226 Gastric cancer	http://www.genome.jp/kegg-bin/show_pathway?hsa05226	CTNNB1 EGF AKT1 AKT2 GSK3B MAPK1 MAPK3 RPS6KB1 TP53
1.48E-11	8	93	48.00629	Path:hsa04912 GnRH signaling pathway	http://www.genome.jp/kegg-bin/show_pathway?hsa04912	MAPK14 PTK2B JUN MAPK1 MAPK3 MAPK8 MAPK9 SRC
1.69E-11	10	223	25.0257	Path:hsa05208 Chemical carcinogenesis-reactive oxygen species	http://www.genome.jp/kegg-bin/show_pathway?hsa05208	MAPK14 EGF AKT1 AKT2 JUN MAPK1 MAPK3 MAPK8 MAPK9 SRC
2.11E-11	9	155	32.40425	Path:hsa04150 mTOR signaling pathway	http://www.genome.jp/kegg-bin/show_pathway?hsa04150	AKT1 AKT2 GSK3B MAPK1 MAPK3 RPS6KA2 RPS6KA3 RPS6KB1 AKT1S1
2.42E-11	10	232	24.05488	Path:hsa05171 Coronavirus disease-COVID-19	http://www.genome.jp/kegg-bin/show_pathway?hsa05171	MAPK14 JUN PLCG1 MAPK1 MAPK3 MAPK8 MAPK9 STAT1 STAT2 STAT3

2.72E-11	8	101	44.20382	Path:hsa05142 Chagas disease	http://www.genome.jp/kegg-bin/show_pathway?hsa05142	MAPK14 AKT1 AKT2 JUN MAPK1 MAPK3 MAPK8 MAPK9
3.94E-11	9	167	30.0758	Path:hsa05225 Hepatocellular carcinoma	http://www.genome.jp/kegg-bin/show_pathway?hsa05225	CTNNB1 AKT1 AKT2 GSK3B PLCG1 MAPK1 MAPK3 RPS6KB1 TP53
6.22E-11	11	354	17.34126	Path:hsa04151 PI3K-Akt signaling pathway	http://www.genome.jp/kegg-bin/show_pathway?hsa04151	CREB1 EGF AKT1 AKT2 GSK3B NOS3 PDGFRB MAPK1 MAPK3 RPS6KB1 TP53
1.08E-10	7	70	55.80732	Path:hsa05120 Epithelial cell signaling in Helicobacter pylori infection	http://www.genome.jp/kegg-bin/show_pathway?hsa05120	MAPK14 JUN LYN PLCG1 MAPK8 MAPK9 SRC
1.30E-10	7	72	54.25711	Path:hsa05218 Melanoma	http://www.genome.jp/kegg-bin/show_pathway?hsa05218	EGF AKT1 AKT2 PDGFRB MAPK1 MAPK3 TP53
1.33E-10	8	124	36.00472	Path:hsa04611 Platelet activation	http://www.genome.jp/kegg-bin/show_pathway?hsa04611	MAPK14 AKT1 AKT2 LYN NOS3 MAPK1 MAPK3 SRC
1.86E-10	7	76	51.40148	Path:hsa05220 Chronic myeloid leukemia	http://www.genome.jp/kegg-bin/show_pathway?hsa05220	AKT1 AKT2 MAPK1 MAPK3 STAT5A STAT5B TP53
2.14E-10	8	132	33.82262	Path:hsa04728 Dopaminergic synapse	http://www.genome.jp/kegg-bin/show_pathway?hsa04728	CREB1 MAPK14 AKT1 AKT2 GSK3A GSK3B MAPK8 MAPK9
2.54E-10	6	41	81.66924	Path:hsa05219 Bladder cancer	http://www.genome.jp/kegg-bin/show_pathway?hsa05219	EGF MAPK1 MAPK3 SRC TP53 RPS6KA5
2.65E-10	8	136	32.82783	Path:hsa04210 Apoptosis	http://www.genome.jp/kegg-bin/show_pathway?hsa04210	AKT1 AKT2 JUN MAPK1 MAPK3 MAPK8 MAPK9 TP53
2.74E-10	7	81	48.22855	Path:hsa04662 B cell receptor signaling pathway	http://www.genome.jp/kegg-bin/show_pathway?hsa04662	AKT1 AKT2 GSK3B JUN LYN MAPK1 MAPK3
2.74E-10	8	137	32.58821	Path:hsa04910 Insulin signaling pathway	http://www.genome.jp/kegg-bin/show_pathway?hsa04910	AKT1 AKT2 GSK3B MAPK1 MAPK3 MAPK8 MAPK9 RPS6KB1
2.86E-10	8	138	32.35207	Path:hsa04915 Estrogen signaling pathway	http://www.genome.jp/kegg-bin/show_pathway?hsa04915	CREB1 AKT1 AKT2 JUN NOS3 MAPK1 MAPK3 SRC
3.35E-10	8	141	31.66373	Path:hsa04140 Autophagy-animal	http://www.genome.jp/kegg-bin/show_pathway?hsa04140	AKT1 AKT2 MAPK1 MAPK3 MAPK8 MAPK9 RPS6KB1 AKT1S1
3.70E-10	8	143	31.22088	Path:hsa04550 Signaling pathways regulating pluripotency of stem cells	http://www.genome.jp/kegg-bin/show_pathway?hsa04550	MAPK14 CTNNB1 AKT1 AKT2 GSK3B MAPK1 MAPK3 STAT3
4.82E-10	8	148	30.16612	Path:hsa04072 Phospholipase D signaling pathway	http://www.genome.jp/kegg-bin/show_pathway?hsa04072	EGF AKT1 AKT2 PTK2B PDGFRB PLCG1 MAPK1 MAPK3

6.72E-10	9	235	21.37302	Path:hsa04014 Ras signaling pathway	http://www.genome.jp/kegg-bin/show_pathway?hsa04014	EGF AKT1 AKT2 PDGFRB PLCG1 MAPK1 MAPK3 MAPK8 MAPK9
6.72E-10	7	93	42.00551	Path:hsa04657 IL-17 signaling pathway	http://www.genome.jp/kegg-bin/show_pathway?hsa04657	MAPK14 GSK3B JUN MAPK1 MAPK3 MAPK8 MAPK9
6.72E-10	8	155	28.80378	Path:hsa04932 Non-alcoholic fatty liver disease	http://www.genome.jp/kegg-bin/show_pathway?hsa04932	MAPK14 AKT1 AKT2 GSK3A GSK3B JUN MAPK8 MAPK9
8.12E-10	8	159	28.07915	Path:hsa04217 Necroptosis	http://www.genome.jp/kegg-bin/show_pathway?hsa04217	MAPK8 MAPK9 STAT1 STAT2 STAT3 STAT5A STAT5B STAT6
8.84E-10	7	97	40.27332	Path:hsa04666 Fc gamma R-mediated phagocytosis	http://www.genome.jp/kegg-bin/show_pathway?hsa04666	AKT1 AKT2 LYN PLCG1 MAPK1 MAPK3 RPS6KB1
1.06E-09	9	249	20.17132	Path:hsa05132 Salmonella infection	http://www.genome.jp/kegg-bin/show_pathway?hsa05132	MAPK14 CTNNB1 AKT1 AKT2 JUN MAPK1 MAPK3 MAPK8 MAPK9
2.40E-09	8	183	24.39664	Path:hsa04621 NOD-like receptor signaling pathway	http://www.genome.jp/kegg-bin/show_pathway?hsa04621	MAPK14 JUN MAPK1 MAPK3 MAPK8 MAPK9 STAT1 STAT2
5.89E-09	6	70	47.83484	Path:hsa05230 Central carbon metabolism in cancer	http://www.genome.jp/kegg-bin/show_pathway?hsa05230	AKT1 AKT2 PDGFRB MAPK1 MAPK3 TP53
9.63E-09	6	76	44.05841	Path:hsa05133 Pertussis	http://www.genome.jp/kegg-bin/show_pathway?hsa05133	MAPK14 JUN MAPK1 MAPK3 MAPK8 MAPK9
1.02E-08	8	221	20.20174	Path:hsa04024 cAMP signaling pathway	http://www.genome.jp/kegg-bin/show_pathway?hsa04024	CREB1 AKT1 AKT2 JUN MAPK1 MAPK3 MAPK8 MAPK9
1.15E-08	7	141	27.70576	Path:hsa04936 Alcoholic liver disease	http://www.genome.jp/kegg-bin/show_pathway?hsa04936	MAPK14 CTNNB1 AKT1 AKT2 GSK3B MAPK8 MAPK9
1.66E-08	7	149	26.2182	Path:hsa04261 Adrenergic signaling in cardiomyocytes	http://www.genome.jp/kegg-bin/show_pathway?hsa04261	CREB1 MAPK14 AKT1 AKT2 MAPK1 MAPK3 RPS6KA5
2.26E-08	7	156	25.04174	Path:hsa04218 Cellular senescence	http://www.genome.jp/kegg-bin/show_pathway?hsa04218	CHEK2 MAPK14 AKT1 AKT2 MAPK1 MAPK3 TP53
2.38E-08	6	89	37.62291	Path:hsa04211 Longevity regulating pathway	http://www.genome.jp/kegg-bin/show_pathway?hsa04211	CREB1 AKT1 AKT2 RPS6KB1 TP53 AKT1S1
2.76E-08	7	161	24.26405	Path:hsa05206 MicroRNAs in cancer	http://www.genome.jp/kegg-bin/show_pathway?hsa05206	PDGFRB PLCG1 MAPK1 MAPK3 STAT3 TP53 RPS6KA5
8.24E-08	7	189	20.66938	Path:hsa04613 Neutrophil extracellular trap formation	http://www.genome.jp/kegg-bin/show_pathway?hsa04613	MAPK14 AKT1 AKT2 PLCG1 MAPK1 MAPK3 SRC
1.08E-07	7	197	19.83001	Path:hsa05130 Pathogenic Escherichia coli infection	http://www.genome.jp/kegg-bin/show_pathway?hsa05130	MAPK14 JUN MAPK1 MAPK3 MAPK8 MAPK9 SRC

1.32E-07	7	203	19.24 39	Path:hsa05415 Diabetic cardiomyopathy	http://www.genome.jp/kegg-bin/show_pathway?hsa05415	MAPK14 AKT1 AKT2 GSK3B NOS3 MAPK8 MAPK9
2.64E-07	5	68	41.03 479	Path:hsa05211 Renal cell carcinoma	http://www.genome.jp/kegg-bin/show_pathway?hsa05211	AKT1 AKT2 JUN MAPK1 MAPK3
2.81E-07	5	69	40.44 008	Path:hsa04920 Adipocytokine signaling pathway	http://www.genome.jp/kegg-bin/show_pathway?hsa04920	AKT1 AKT2 MAPK8 MAPK9 STAT3
3.06E-07	6	138	24.26 405	Path:hsa04371 Apelin signaling pathway	http://www.genome.jp/kegg-bin/show_pathway?hsa04371	AKT1 AKT2 NOS3 MAPK1 MAPK3 RPS6KB1
3.19E-07	5	71	39.30 093	Path:hsa04520 Adherens junction	http://www.genome.jp/kegg-bin/show_pathway?hsa04520	CTNNB1 MAPK1 MAPK3 SRC YES1
3.39E-07	5	72	38.75 508	Path:hsa04137 Mitophagy-animal	http://www.genome.jp/kegg-bin/show_pathway?hsa04137	JUN MAPK8 MAPK9 SRC TP53
3.59E-07	5	73	38.22 419	Path:hsa01524 Platinum drug resistance	http://www.genome.jp/kegg-bin/show_pathway?hsa01524	AKT1 AKT2 MAPK1 MAPK3 TP53
4.36E-07	5	76	36.71 534	Path:hsa05140 Leishmaniasis	http://www.genome.jp/kegg-bin/show_pathway?hsa05140	MAPK14 JUN MAPK1 MAPK3 STAT1
6.16E-07	8	384	11.62 652	Path:hsa05010 Alzheimer disease	http://www.genome.jp/kegg-bin/show_pathway?hsa05010	CTNNB1 AKT1 AKT2 GSK3B MAPK1 MAPK3 MAPK8 MAPK9
8.60E-07	6	166	20.17 132	Path:hsa04022 cGMP-PKG signaling pathway	http://www.genome.jp/kegg-bin/show_pathway?hsa04022	CREB1 AKT1 AKT2 NOS3 MAPK1 MAPK3
8.81E-07	5	88	31.70 87	Path:hsa04540 Gap junction	http://www.genome.jp/kegg-bin/show_pathway?hsa04540	EGF PDGFRB MAPK1 MAPK3 SRC
8.81E-07	7	273	14.30 957	Path:hsa05020 Prion disease	http://www.genome.jp/kegg-bin/show_pathway?hsa05020	CREB1 MAPK14 GSK3B MAPK1 MAPK3 MAPK8 MAPK9
9.58E-07	6	170	19.69 67	Path:hsa04310 Wnt signaling pathway	http://www.genome.jp/kegg-bin/show_pathway?hsa04310	CTNNB1 GSK3B JUN MAPK8 MAPK9 TP53
9.58E-07	4	37	60.33 223	Path:hsa05216 Thyroid cancer	http://www.genome.jp/kegg-bin/show_pathway?hsa05216	CTNNB1 MAPK1 MAPK3 TP53
9.77E-07	6	171	19.58 151	Path:hsa05164 Influenza A	http://www.genome.jp/kegg-bin/show_pathway?hsa05164	AKT1 AKT2 MAPK1 MAPK3 STAT1 STAT2
1.44E-06	5	98	28.47 312	Path:hsa04750 Inflammatory mediator regulation of TRP channels	http://www.genome.jp/kegg-bin/show_pathway?hsa04750	MAPK14 PLCG1 MAPK8 MAPK9 SRC
1.66E-06	5	101	27.62 738	Path:hsa04916 Melanogenesis	http://www.genome.jp/kegg-bin/show_pathway?hsa04916	CREB1 CTNNB1 GSK3B MAPK1 MAPK3

2.26E-06	4	46	48.52 81	Path:hsa04930 Type II diabetes mellitus	http://www.genome.jp/kegg-bin/show_pathway?hsa04930	MAPK1 MAPK3 MAPK8 MAPK9
2.82E-06	8	476	9.379 381	Path:hsa05022 Pathways of neurodegeneration-multiple diseases	http://www.genome.jp/kegg-bin/show_pathway?hsa05022	MAPK14 CTNNB1 GSK3B PLCG1 MAPK1 MAPK3 MAPK8 MAPK9
2.83E-06	5	113	24.69 35	Path:hsa04725 Cholinergic synapse	http://www.genome.jp/kegg-bin/show_pathway?hsa04725	CREB1 AKT1 AKT2 MAPK1 MAPK3
3.93E-06	5	121	23.06 087	Path:hsa04152 AMPK signaling pathway	http://www.genome.jp/kegg-bin/show_pathway?hsa04152	CREB1 AKT1 AKT2 RPS6KB1 AKT1S1
5.41E-06	6	232	14.43 293	Path:hsa04714 Thermogenesis	http://www.genome.jp/kegg-bin/show_pathway?hsa04714	CREB1 MAPK14 RPS6KA2 RPS6KA3 RPS6KB1 AKT1S1
5.49E-06	5	130	21.46 435	Path:hsa04650 Natural killer cell mediated cytotoxicity	http://www.genome.jp/kegg-bin/show_pathway?hsa04650	PTK2B LCK PLCG1 MAPK1 MAPK3
5.66E-06	5	131	21.30 05	Path:hsa04114 Oocyte meiosis	http://www.genome.jp/kegg-bin/show_pathway?hsa04114	MAPK14 MAPK1 MAPK3 RPS6KA2 RPS6KA3
7.11E-06	4	62	36.00 472	Path:hsa04213 Longevity regulating pathway-multiple species	http://www.genome.jp/kegg-bin/show_pathway?hsa04213	AKT1 AKT2 RPS6KB1 AKT1S1
8.02E-06	4	64	34.87 957	Path:hsa04929 GnRH secretion	http://www.genome.jp/kegg-bin/show_pathway?hsa04929	AKT1 AKT2 MAPK1 MAPK3
8.46E-06	4	65	34.34 296	Path:hsa05321 Inflammatory bowel disease	http://www.genome.jp/kegg-bin/show_pathway?hsa05321	JUN STAT1 STAT3 STAT6
9.48E-06	4	67	33.31 78	Path:hsa04720 Long-term potentiation	http://www.genome.jp/kegg-bin/show_pathway?hsa04720	MAPK1 MAPK3 RPS6KA2 RPS6KA3
9.86E-06	5	148	18.85 382	Path:hsa04723 Retrograde endocannabinoid signaling	http://www.genome.jp/kegg-bin/show_pathway?hsa04723	MAPK14 MAPK1 MAPK3 MAPK8 MAPK9
1.19E-05	5	154	18.11 926	Path:hsa04921 Oxytocin signaling pathway	http://www.genome.jp/kegg-bin/show_pathway?hsa04921	JUN NOS3 MAPK1 MAPK3 SRC
1.21E-05	5	155	18.00 236	Path:hsa04934 Cushing syndrome	http://www.genome.jp/kegg-bin/show_pathway?hsa04934	CREB1 CTNNB1 GSK3B MAPK1 MAPK3
2.55E-05	5	181	15.41 639	Path:hsa04360 Axon guidance	http://www.genome.jp/kegg-bin/show_pathway?hsa04360	GSK3B PLCG1 MAPK1 MAPK3 SRC
3.96E-05	4	97	23.01 333	Path:hsa04713 Circadian entrainment	http://www.genome.jp/kegg-bin/show_pathway?hsa04713	CREB1 MAPK1 MAPK3 RPS6KA5
5.99E-05	5	217	12.85 883	Path:hsa04810 Regulation of actin cytoskeleton	http://www.genome.jp/kegg-bin/show_pathway?hsa04810	EGF PDGFRB MAPK1 MAPK3 SRC

7.35E-05	4	114	19.58151	Path:hsa04670 Leukocyte transendothelial migration	http://www.genome.jp/kegg-bin/show_pathway?hsa04670	MAPK14 CTNNB1 PTK2B PLCG1
9.52E-05	5	240	11.62652	Path:hsa04020 Calcium signaling pathway	http://www.genome.jp/kegg-bin/show_pathway?hsa04020	EGF PTK2B NOS3 PDGFRB PLCG1
0.000169741	4	142	15.72037	Path:hsa05017 Spinocerebellar ataxia	http://www.genome.jp/kegg-bin/show_pathway?hsa05017	AKT1 AKT2 MAPK8 MAPK9
0.000238049	3	60	27.90366	Path:hsa04730 Long-term depression	http://www.genome.jp/kegg-bin/show_pathway?hsa04730	LYN MAPK1 MAPK3
0.000273165	3	63	26.57491	Path:hsa05217 Basal cell carcinoma	http://www.genome.jp/kegg-bin/show_pathway?hsa05217	CTNNB1 GSK3B TP53
0.000321634	4	169	13.20883	Path:hsa04530 Tight junction	http://www.genome.jp/kegg-bin/show_pathway?hsa04530	JUN MAPK8 MAPK9 SRC
0.000321634	6	495	6.764523	Path:hsa05168 Herpes simplex virus 1 infection	http://www.genome.jp/kegg-bin/show_pathway?hsa05168	AKT1 AKT2 SRC STAT1 STAT2 TP53
0.000365111	3	70	23.91742	Path:hsa04622 RIG-I-like receptor signaling pathway	http://www.genome.jp/kegg-bin/show_pathway?hsa04622	MAPK14 MAPK8 MAPK9
0.000809131	3	92	18.19804	Path:hsa05222 Small cell lung cancer	http://www.genome.jp/kegg-bin/show_pathway?hsa05222	AKT1 AKT2 TP53
0.000829057	3	93	18.00236	Path:hsa04350 TGF-beta signaling pathway	http://www.genome.jp/kegg-bin/show_pathway?hsa04350	MAPK1 MAPK3 RPS6KB1
0.00113947	3	104	16.09826	Path:hsa04064 NF-kappa B signaling pathway	http://www.genome.jp/kegg-bin/show_pathway?hsa04064	LCK LYN PLCG1
0.001187198	3	106	15.79452	Path:hsa04922 Glucagon signaling pathway	http://www.genome.jp/kegg-bin/show_pathway?hsa04922	CREB1 AKT1 AKT2
0.001187198	3	106	15.79452	Path:hsa04928 Parathyroid hormone synthesis secretion and action	http://www.genome.jp/kegg-bin/show_pathway?hsa04928	CREB1 MAPK1 MAPK3
0.001677319	4	266	8.392078	Path:hsa05012 Parkinson disease	http://www.genome.jp/kegg-bin/show_pathway?hsa05012	PLCG1 MAPK8 MAPK9 TP53
0.001926409	3	126	13.28746	Path:hsa04110 Cell cycle	http://www.genome.jp/kegg-bin/show_pathway?hsa04110	CHEK2 GSK3B TP53
0.001926409	2	32	34.87957	Path:hsa04215 Apoptosis-multiple species	http://www.genome.jp/kegg-bin/show_pathway?hsa04215	MAPK8 MAPK9
0.002554441	2	37	30.16612	Path:hsa04960 Aldosterone-regulated sodium reabsorption	http://www.genome.jp/kegg-bin/show_pathway?hsa04960	MAPK1 MAPK3

0.002722232	4	306	7.295074	Path:hsa05016 Huntington disease	http://www.genome.jp/kegg-bin/show_pathway?hsa05016	CREB1 MAPK8 MAPK9 TP53
0.004044099	2	47	23.74779	Path:hsa04973 Carbohydrate digestion and absorption	http://www.genome.jp/kegg-bin/show_pathway?hsa04973	AKT1 AKT2
0.004360098	2	49	22.7785	Path:hsa05030 Cocaine addiction	http://www.genome.jp/kegg-bin/show_pathway?hsa05030	CREB1 JUN
0.005568004	3	186	9.00118	Path:hsa05034 Alcoholism	http://www.genome.jp/kegg-bin/show_pathway?hsa05034	CREB1 MAPK1 MAPK3
0.005986208	2	58	19.2439	Path:hsa04923 Regulation of lipolysis in adipocytes	http://www.genome.jp/kegg-bin/show_pathway?hsa04923	AKT1 AKT2
0.008335759	2	69	16.17603	Path:hsa05031 Amphetamine addiction	http://www.genome.jp/kegg-bin/show_pathway?hsa05031	CREB1 JUN
0.009234948	2	73	15.28968	Path:hsa04115 p53 signaling pathway	http://www.genome.jp/kegg-bin/show_pathway?hsa04115	CHEK2 TP53
0.010169387	2	77	14.49541	Path:hsa05100 Bacterial invasion of epithelial cells	http://www.genome.jp/kegg-bin/show_pathway?hsa05100	CTNNB1 SRC
0.020635491	2	112	9.965592	Path:hsa04726 Serotonergic synapse	http://www.genome.jp/kegg-bin/show_pathway?hsa04726	MAPK1 MAPK3
0.021197704	2	114	9.790757	Path:hsa04724 Glutamatergic synapse	http://www.genome.jp/kegg-bin/show_pathway?hsa04724	MAPK1 MAPK3
0.028493496	2	134	8.32945	Path:hsa04270 Vascular smooth muscle contraction	http://www.genome.jp/kegg-bin/show_pathway?hsa04270	MAPK1 MAPK3
0.037921209	2	157	7.109212	Path:hsa04390 Hippo signaling pathway	http://www.genome.jp/kegg-bin/show_pathway?hsa04390	CTNNB1 GSK3B
0.043101875	2	169	6.604416	Path:hsa04141 Protein processing in endoplasmic reticulum	http://www.genome.jp/kegg-bin/show_pathway?hsa04141	MAPK8 MAPK9
0.044995573	1	22	25.36696	Path:hsa00220 Arginine biosynthesis	http://www.genome.jp/kegg-bin/show_pathway?hsa00220	NOS3
0.068390279	1	34	16.41392	Path:hsa04710 Circadian rhythm	http://www.genome.jp/kegg-bin/show_pathway?hsa04710	CREB1
0.075701509	1	38	14.68614	Path:hsa05340 Primary immunodeficiency	http://www.genome.jp/kegg-bin/show_pathway?hsa05340	LCK
0.080966287	1	41	13.61154	Path:hsa04216 Ferroptosis	http://www.genome.jp/kegg-bin/show_pathway?hsa04216	TP53

0.084252169	1	43	12.97845	Path:hsa04940 Type I diabetes mellitus	http://www.genome.jp/kegg-bin/show_pathway?hsa04940	HSPD1
0.085614953	1	44	12.68348	Path:hsa04962 Vasopressin-regulated water reabsorption	http://www.genome.jp/kegg-bin/show_pathway?hsa04962	CREB1
0.095627537	1	50	11.16146	Path:hsa00330 Arginine and proline metabolism	http://www.genome.jp/kegg-bin/show_pathway?hsa00330	NOS3
0.095627537	1	50	11.16146	Path:hsa05110 Vibrio cholerae infection	http://www.genome.jp/kegg-bin/show_pathway?hsa05110	PLCG1
0.1059157	1	56	9.965592	Path:hsa04340 Hedgehog signaling pathway	http://www.genome.jp/kegg-bin/show_pathway?hsa04340	GSK3B
0.107076819	1	57	9.790757	Path:hsa05134 Legionellosis	http://www.genome.jp/kegg-bin/show_pathway?hsa05134	HSPD1
0.120553841	1	65	8.585741	Path:hsa04927 Cortisol synthesis and secretion	http://www.genome.jp/kegg-bin/show_pathway?hsa04927	CREB1
0.126787566	1	69	8.088017	Path:hsa04924 Renin secretion	http://www.genome.jp/kegg-bin/show_pathway?hsa04924	CREB1
0.132900549	1	73	7.644838	Path:hsa00562 Inositol phosphate metabolism	http://www.genome.jp/kegg-bin/show_pathway?hsa00562	PLCG1
0.13551971	1	75	7.440976	Path:hsa04918 Thyroid hormone synthesis	http://www.genome.jp/kegg-bin/show_pathway?hsa04918	CREB1
0.138096962	1	77	7.247704	Path:hsa05412 Arrhythmogenic right ventricular cardiomyopathy	http://www.genome.jp/kegg-bin/show_pathway?hsa05412	CTNNB1
0.138971909	1	78	7.154784	Path:hsa04612 Antigen processing and presentation	http://www.genome.jp/kegg-bin/show_pathway?hsa04612	CREB1
0.139834012	1	79	7.064217	Path:hsa03018 RNA degradation	http://www.genome.jp/kegg-bin/show_pathway?hsa03018	HSPD1
0.14534582	2	364	3.066336	Path:hsa05014 Amyotrophic lateral sclerosis	http://www.genome.jp/kegg-bin/show_pathway?hsa05014	MAPK14 TP53
0.149614998	1	86	6.489223	Path:hsa04911 Insulin secretion	http://www.genome.jp/kegg-bin/show_pathway?hsa04911	CREB1
0.153574867	1	89	6.270485	Path:hsa04727 GABAergic synapse	http://www.genome.jp/kegg-bin/show_pathway?hsa04727	SRC
0.157465673	1	92	6.066013	Path:hsa05323 Rheumatoid arthritis	http://www.genome.jp/kegg-bin/show_pathway?hsa05323	JUN

0.164403634	1	97	5.753332	Path:hsa04070 Phosphatidylinositol signaling system	http://www.genome.jp/kegg-bin/show_pathway?hsa04070	PLCG1
0.165045156	1	98	5.694624	Path:hsa04925 Aldosterone synthesis and secretion	http://www.genome.jp/kegg-bin/show_pathway?hsa04925	CREB1
0.170261926	1	102	5.471306	Path:hsa05146 Amoebiasis	http://www.genome.jp/kegg-bin/show_pathway?hsa05146	HSPB1

Table S3: Oncoproteome Profiler/ phosphokinase array data following normalization and correction for membrane-to-membrane variation. Raw OncoBreast Profiler measurements are shown following normalization and the relevant calculations applied to account for differences between assay membranes. Values are presented for the indicated protein targets across the control and treatment conditions (perifosine, GSK690693, GDC-0941, and rapamycin).

Absolute fold change values with control as 1

Type	Control	Perifosine	GSK690693	GDC-0941	Rapamycin
beta catenin	0.099507025	0.298476742	0.245507858	0.180455018	0.096448928
CREB	0.017128538	0.107830482	-0.019341937	0.079786589	0.094682059
EGF R	0.025182473	0.094942891	0.165500886	0.054258049	0.047657231
eNOS	0.020560972	0.076081949	0.004763035	0.041797327	0.160724795
ERK1/2	0.056180437	0.194356167	0.033220997	0.107556045	0.034656633
Fgr	0.03871706	0.132437918	0.036934341	0.132436382	0.1079884
GSK-3aplha/beta	0.040583113	0.126748439	0.024038996	0.065234604	0.015691571
GSK-3beta	0.043845926	0.126128726	0.101886059	0.058910563	-0.019325078
HSP27	0.014082341	0.080055211	0.017570277	0.027186717	0.215580175
Jnk1/2/3	0.053379822	0.212561701	0.020146961	0.147937412	0.11214401
Lck	0.042962345	0.081599283	0.055231853	0.073551894	0.104955442
Lyn	0.028366766	0.154267669	0.052215573	0.048735668	0.095240624
MSK1/2	0.030637451	0.109824683	0.155644022	0.052153034	0.030722686
p38alpha	0.03725202	0.119384911	0.082619351	0.108341576	0.174194394
PDGF Rbeta	0.051818195	0.169458934	0.246587136	0.143253336	0.215817784
PLC - Y1	0.100920645	0.280014253	0.002489789	0.140954965	0.273839756
Src	0.076094874	0.293598578	0.05985884	0.160419016	0.026038683
STAT 2	0.034625452	0.166261439	0.051556215	0.13392918	0.211081776
STAT 5a/b	0.041696147	0.189048903	0.039058775	0.158203225	0.142315738
WNK 1	0.061043314	0.154282261	0.00428972	0.117169778	0.125152069
Yes	0.025163712	0.108121303	0.062310082	0.068814862	0.002357691
STAT 1	-0.005957318	-0.004445114	0.114650951	0.00564903	0.008066553
STAT 2	-0.004410429	-0.001574464	0.116902437	-0.006264062	0.0035333
STAT 3	0.001131554	-0.004385977	0.037007302	0.001158127	0.003909151
STAT6	0.107429374	-0.004096326	0.608866759	0.019045521	0.062804354
hsp60	0.950205785	0.674655356	-0.076230797	0.329865077	0.725860516
PYK2	0.30390416	0.203344518	0.348075987	0.109189463	0.196319574
RSK 1/2	0.097421759	0.066128864	0.038415911	0.022641724	0.063807489
p70 s6 Kinase	0.182042223	0.091110358	0.317991995	0.062264879	0.116072653
p70 s6 Kinase	0.055956251	0.039648536	0.190436191	0.028778991	0.048254161

p53	0.170542103	0.095254887	0.144058031	0.054653064	0.178391184
p53	0.375089696	0.375939872	0.078507847	0.212865651	0.426159927
Chk-2	0.208442056	0.194191081	0.157388473	0.172372264	0.212810903
c-Jun	0.078512981	0.143748814	0.232941443	0.050420653	0.149659809
Akt1/2/3	0.161879134	0.264379004	-0.082299711	0.138039989	0.22800491
Akt1/2/3	0.092979388	0.132927508	0.128183271	0.035529718	0.161130087
p53	0.074482338	0.032120507	0.075360785	0.039339209	0.07418854
PRAS40	0.111354945	0.073536314	-0.006991883	0.035214942	0.130684287
RSK 1/2/3	0.073152103	0.106951509	0.059087609	0.070891533	0.137771769

Table S4. Absolute fold-change values from the Proteome Profiler Human Phospho-Kinase Array. Absolute fold-change values are shown for the indicated phosphoprotein targets following treatment with perifosine, GSK690693, GDC-0941, or rapamycin. Values for the control condition were normalized to 1, and the corresponding treatment values are expressed relative to the control. Reference spots and the PBS negative-control spot are included as array controls. Values represent the processed array measurements after the relevant normalization and calculations used to account for differences between membranes.

- FDR calculations

Target	Control	Perifosine	GSK690693	GDC-0941	Rapamycin
Reference spot	1	1.0310768	1.144E-06	1.071273	1.55841642
Reference spot	1	0.9689247	8.559E-07	0.928727	0.44158335
beta catenin	1	1.7018994	1.241E-06	0.789148	0.60076588
CREB	1	3.5718925	-5.678E-07	2.026996	3.42617068
EGF R	1	2.1391498	3.305E-06	0.937582	1.17298383
eNOS	1	2.0994964	1.165E-07	0.884603	4.84507872
ERK1/2	1	1.9628646	2.974E-07	0.833092	0.38235154
Fgr	1	1.9408282	4.797E-07	1.488498	1.72876645
GSK-3aplha/beta	1	1.7720436	2.979E-07	0.699481	0.23965288
GSK-3beta	1	1.6321569	1.169E-06	0.584665	-0.273183
HSP27	1	3.2254615	6.274E-07	0.840089	9.48845592
Jnk1/2/3	1	2.2593579	1.898E-07	1.205992	1.30214861
Lck	1	1.0776445	6.465E-07	0.744988	1.51418376
Lyn	1	3.0856185	9.256E-07	0.747619	2.08100923
MSK1/2	1	2.0338759	2.555E-06	0.740748	0.62153866
p38alpha	1	1.8183469	1.115E-06	1.265577	2.89831737
PDGF Rbeta	1	1.855493	2.393E-06	1.203001	2.58146678
PLC - Y1	1	1.5742629	1.241E-08	0.607776	1.68181444
Src	1	2.1891508	3.956E-07	0.917368	0.21209245
STAT 2	1	2.7244147	7.488E-07	1.683151	3.77847792
STAT 5a/b	1	2.5724999	4.711E-07	1.65106	2.11552713

WNK 1	1	1.4340194	3.534E-08	0.835258	1.27075411
Yes	1	2.4378877	1.245E-06	1.190011	0.05807293
STAT 1	1	0.4233595	-9.678E-06	-0.412635	-0.8392644
STAT 2	1	0.2025484	-1.333E-05	0.618043	-0.496548
STAT 3	1	-2.199217	1.645E-05	0.445373	2.14125645
PBS (Negtive Control)	1	-0.192442	-6.524E-06	-0.238694	-0.7865291
STAT6	1	-0.021635	2.85E-06	0.077146	0.36235003
hsp60	1	0.4028482	-4.034E-08	0.151064	0.47347505
PYK2	1	0.3796409	5.76E-07	0.156346	0.40039492
RSK 1/2	1	0.3851343	1.983E-07	0.101134	0.40595442
p70 s6 Kinase (Site 1)	1	0.2839702	8.784E-07	0.148838	0.39520222
p70 s6 Kinase (Site 2)	1	0.4020274	1.711E-06	0.223805	0.53450005
p53 (Site 1)	1	0.3169077	4.248E-07	0.139452	0.64834082
p53 (Site 2)	1	0.5686701	1.053E-07	0.246953	0.70420494
Chk-2	1	0.5285926	3.797E-07	0.359853	0.63280532
c-Jun	1	1.0388191	1.492E-06	0.279454	1.18147711
Akt1/2/3 (Site 1)	1	0.9266447	-2.557E-07	0.371071	0.87300137
Akt1/2/3 (Site 2)	1	0.8111577	6.933E-07	0.166283	1.0741169
p53 (Site 3)	1	0.2446844	5.088E-07	0.229835	0.61736943
PRAS40	1	0.3746877	-3.157E-08	0.137613	0.72740362
RSK 1/2/3	1	0.8295398	4.062E-07	0.421707	1.16733368

Table S5: Calculation of false discovery rate (FDR)-adjusted p-values. The figure illustrates the calculation and multiple-testing correction applied to the protein-marker data. P-values obtained from the statistical comparisons are ranked and adjusted for multiple testing using the Benjamini–Hochberg procedure. The resulting adjusted p-values represent the FDR-controlled significance values used to determine which protein markers meet the predefined statistical significance threshold. The calculations shown provide the basis for the FDR-corrected results reported in the subsequent analyses.

FDR Summary after calculations :

	Perifosine			GSK690693			GDC-0941			Rapamycin		
	Discovery?	P value	q value	Discovery?	P value	q value	Discovery?	P value	q value	Discovery?	P value	q value
beta catenin	Yes	0.005706	0.023298	Yes	0.006034	0.025482	No	0.020697	0.061721	No	0.702502	0.761044
CREB	Yes	0.01092	0.033033	No	0.024315	0.070103	Yes	0.00583	0.032482	Yes	0.018142	0.042068
EGF R	Yes	0.00212	0.022605	Yes	0.00596	0.025482	No	0.023488	0.061721	Yes	0.022541	0.046268
eNOS	Yes	0.006265	0.023298	No	0.487123	0.593681	No	0.01907	0.061721	Yes	0.014955	0.042068
ERK1/2	Yes	0.017516	0.042694	No	0.452296	0.580754	No	0.045548	0.080744	No	0.453273	0.570247
Fgr	No	0.070978	0.110725	No	0.906248	0.930096	Yes	0.011453	0.044668	Yes	0.002815	0.024004
GSK-3aplha/beta	Yes	0.00548	0.023298	No	0.127215	0.236256	No	0.221098	0.287428	No	0.344954	0.463903
GSK-3beta	Yes	0.006571	0.023298	No	0.459812	0.580754	No	0.344374	0.406988	Yes	0.011554	0.042068
HSP27	Yes	0.018628	0.042736	No	0.876086	0.923442	No	0.291273	0.366441	Yes	0.002016	0.024004
Jnk1/2/3	No	0.084511	0.123949	No	0.037049	0.085202	Yes	0.004863	0.03161	Yes	0.013685	0.042068
Lck	No	0.130224	0.172171	No	0.619421	0.710513	No	0.056108	0.09514	Yes	0.015847	0.042068
Lyn	Yes	0.004988	0.023298	No	0.148343	0.262972	No	0.409665	0.469909	Yes	0.006483	0.028092
MSK1/2	Yes	0.004443	0.023298	No	0.296717	0.482166	No	0.218023	0.287428	No	0.990073	0.99458
p38alpha	Yes	0.014806	0.041246	No	0.037139	0.085202	Yes	0.010662	0.044668	Yes	0.003057	0.024004
PDGF Rbeta	Yes	0.002898	0.022605	Yes	0.001951	0.025482	Yes	0.001126	0.013233	Yes	0.003077	0.024004
PLC - Y1	Yes	0.001381	0.022605	Yes	0.005258	0.025482	No	0.034587	0.064232	Yes	0.001718	0.024004
Src	Yes	0.00024	0.00935	No	0.370093	0.555139	No	0.0145	0.051408	Yes	0.018337	0.042068
STAT 2 (entry 1)	No	0.048339	0.085691	No	0.460182	0.580754	No	0.029304	0.064232	Yes	0.012344	0.042068
STAT 2 (entry 2)	No	0.477211	0.516978	Yes	0.006534	0.025482	No	0.798023	0.819024	No	0.156132	0.253715
STAT 5a/b	Yes	0.002335	0.022605	No	0.828336	0.897364	Yes	0.001513	0.013233	Yes	0.006232	0.028092
WNK 1	No	0.034309	0.066903	Yes	0.006458	0.025482	Yes	0.001697	0.013233	No	0.073234	0.141802
Yes	Yes	0.02119	0.043495	No	0.040334	0.087389	No	0.033182	0.064232	No	0.132653	0.224934
STAT 1	No	0.770623	0.790902	No	0.32983	0.514535	No	0.313197	0.381708	No	0.088206	0.156365
STAT 3	No	0.189367	0.223798	No	0.506592	0.5987	No	0.997714	0.997714	No	0.593786	0.701747
STAT6	No	0.166992	0.203521	Yes	0.013635	0.044314	No	0.193016	0.268844	No	0.432272	0.561954
hsp60	Yes	0.020796	0.043495	Yes	0.000393	0.015323	Yes	0.00082	0.013233	Yes	0.00607	0.028092
PYK2	No	0.11541	0.160749	No	0.436906	0.580754	No	0.023739	0.061721	No	0.076355	0.141802
RSK 1/2	No	0.243102	0.278853	No	0.079624	0.155266	No	0.083721	0.130605	No	0.272895	0.380103
p70 s6 Kinase (entry 1)	No	0.132941	0.172171	No	0.254355	0.431297	No	0.0836	0.130605	No	0.219258	0.328887
p70 s6 Kinase (entry 2)	No	0.460459	0.513083	Yes	0.009566	0.033915	No	0.180992	0.261433	No	0.697	0.761044
p53 (entry 1)	Yes	0.017177	0.042694	No	0.640919	0.714167	Yes	0.007267	0.035425	No	0.652114	0.748013
p53 (entry 2)	No	0.991971	0.991971	No	0.034675	0.085202	No	0.118956	0.178435	No	0.472353	0.57568
p53 (entry 3)	Yes	0.011011	0.033033	No	0.95665	0.95665	No	0.029136	0.064232	No	0.99458	0.99458
Chk-2	No	0.726926	0.766219	No	0.461625	0.580754	No	0.42711	0.475923	No	0.942205	0.993135
c-Jun	No	0.085811	0.123949	Yes	0.003506	0.025482	No	0.029872	0.064232	Yes	0.016339	0.042068
Akt1/2/3 (entry 1)	No	0.069398	0.110725	Yes	0.00445	0.025482	No	0.469923	0.495324	No	0.20558	0.320705
Akt1/2/3 (entry 2)	No	0.136854	0.172171	No	0.025165	0.070103	Yes	0.000426	0.013233	Yes	0.020455	0.044319
PRAS40	No	0.038722	0.071912	Yes	0.005615	0.025482	No	0.033569	0.064232	No	0.26666	0.380103
RSK 1/2/3	No	0.057153	0.096911	No	0.053533	0.109884	No	0.464214	0.495324	Yes	0.004582	0.028092

Table S6. Summary of false discovery rate (FDR) analysis following multiple-testing correction. The table summarizes the number of protein-marker comparisons tested and the number identified as statistically significant after FDR correction for each treatment relative to the control condition. A total of 39 protein markers were tested for each comparison. Following multiple-testing correction, 19 markers were FDR-significant for perifosine versus control, 12 for GSK-690693 versus control, 10 for GDC-0941 versus control, and 19 for rapamycin versus control. The number of FDR-significant markers reflects the results obtained after application of the specified FDR correction procedure.

- **Raw data values for each RT-qPCR plate experiment conducted**

Plate 1

comparison	n_tests	n_FDR_significant
Perifosine vs Control	39	19
GSK-690693 vs Control	39	12
GDC-0941 vs Control	39	10
Rapamycin vs Control	39	19

Table S7: the tables represent relative gene expression was determined using the standard 2^(−ΔΔCt) method, with ABL1 serving as the internal control gene. For each sample, ΔCt values were calculated by subtracting ABL1 Ct from the target gene Ct. ΔΔCt values were then derived relative to the control group, and fold-change values were computed accordingly for GSK3α, GSK3β and CREB1.

Statistical test done for validation by using GRAPHPAD Prism v.9 using Kruskal–Wallis

Well	Sample Name	Cт	Cт Mean	Tm1	Tm2	Tm3
A1	NC	20.33298	20.33298	82.58686	87.65673	
A2	Cell line	Undetermined		67.22815		
A3	PC	18.86256	18.86256	82.28624		
A4	GSK 3 alpha Patient - 1	20.25589	20.25589	82.43536	87.35606	
A5	GSK 3 Alpha Patient - 2	20.30442	20.30442	82.28595	87.35545	
A6	GSK 3 Alpha Patient - 3	20.64756	20.64756	87.50455		
A7	GSK 3 Alpha Patient - 4	20.26301	20.26301	81.69023		
A8	GSK 3 Alpha Patient - 5	20.75812	20.75812	81.69023	86.61152	
A9	GSK 3 Alpha Patient - 6	20.79479	20.79479	82.281	87.35116	
A10	GSK 3 Alpha Patient - 7	20.24529	20.24529	82.281	87.35116	
A11	GSK 3 Alpha Patient - 8	19.81419	19.81419	87.20276	81.98322	
A12	GSK 3 Alpha Patient - 9	20.66939	20.66939	82.43061		
B1	GSK 3 Alpha Patient - 10	20.78844	20.78844	82.73598		
B2	GSK 3 Alpha Patient - 11	20.10487	20.10487	87.65673	82.43774	
B3	GSK 3 Alpha Patient - 12	19.78871	19.78871	82.13713	87.35606	
B4	GSK 3 Alpha Patient - 13	20.24762	20.24762	82.28624	87.35606	
B5	GSK 3 Alpha Patient - 14	20.48253	20.48253	87.35545	82.13684	
B6	GSK 3 Alpha Patient - 15	19.67826	19.67826	82.43505		
B7	GSK 3 Alpha Patient - 16	20.22792	20.22792	81.83936		
B8	GSK 3 Alpha Patient - 17	19.69394	19.69394	86.61152	81.39197	
B9	GSK 3 Alpha Patient - 18	20.26987	20.26987	82.43012		
B10	GSK 3 Alpha Patient - 19	19.87333	19.87333	82.13188		
B11	GSK 3 Alpha Patient - 20	20.22165	20.22165	82.28148		
B12	NC	35.85719	35.85719	86.75537	68.85982	
C1	Cell line	20.03475	20.03475	83.0342		

C2	PC	Undetermined		83.63066	72.29801	87.95496
C3	GSK 3 Beta NC2	18.51311	18.51311	82.88269		
C4	GSK 3 Beta Patient - 1	20.66501	20.66501	82.73358		
C5	GSK 3 Beta Patient - 2	21.66597	21.66597	82.88235		
C6	GSK 3 Beta Patient - 3	26.9724	26.9724	82.73325		
C7	GSK 3 Beta Patient - 4	19.06456	19.06456	82.13762		
C8	GSK 3 Beta Patient - 5	20.22287	20.22287	82.13762		
C9	GSK 3 Beta Patient - 6	21.55377	21.55377	82.72836		
C10	GSK 3 Beta Patient - 7	21.13577	21.13577	82.72836		
C11	GSK 3 Beta Patient - 8	21.41875	21.41875	82.57974		
C12	GSK 3 Beta Patient - 9	20.58885	20.58885	82.72887		
D1	GSK 3 Beta Patient - 10	20.4293	20.4293	83.0342		
D2	GSK 3 Beta Patient - 11	21.19568	21.19568	83.0342		
D3	GSK 3 Beta Patient - 12	19.81369	19.81369	82.73358		
D4	GSK 3 Beta Patient - 13	19.27019	19.27019	82.73358		
D5	GSK 3 Beta Patient - 14	21.19285	21.19285	82.88235		
D6	GSK 3 Beta Patient - 15	19.00501	19.00501	82.88235		
D7	GSK 3 Beta Patient - 16	20.49331	20.49331	82.28675		
D8	GSK 3 Beta Patient - 17	21.2931	21.2931	82.13762		
D9	GSK 3 Beta Patient - 18	19.3022	19.3022	82.72836		
D10	GSK 3 Beta Patient - 19	19.26542	19.26542	82.72836		
D11	GSK 3 Beta Patient - 20	19.14201	19.14201	82.72887		
D12	Sample 1	Undetermined	35.85719	69.60547	73.7811	78.70237
E1	NC	19.68324	19.68324	79.7537		
E2	Cell line	Undetermined		65.88612	70.95599	79.30636
E3	PC	18.96932	18.96932	79.45311		
E4	CREB1 Patient - 1	19.68629	19.68629	79.45311		
E5	CREB1 Patient - 2	20.31326	20.31326	79.45299		
E6	CREB1 Patinet - 3	20.15618	20.15618	79.45299		
E7	CREB1 Patient - 4	19.33098	19.33098	79.00589		
E8	CREB1 Patient - 5	19.56552	19.56552	79.00589		
E9	CREB1 Patient - 6	21.0781	21.0781	79.44768		
E10	CREB1 Patient - 7	19.6595	19.6595	79.44768		
E11	CREB1 Patient - 8	19.9817	19.9817	79.29889		
E12	CREB1 Patient - 9	20.70097	20.70097	79.44802		
F1	CREB1 Patient - 10	20.76701	20.76701	79.7537		
F2	CREB1 Patient - 11	20.66344	20.66344	79.7537		

F3	CREB1 Patient - 12	20.10471	20.10471	79.45311		
F4	CREB1 Patient - 13	20.22733	20.22733	79.45311		
F5	CREB1 Patient - 14	20.79045	20.79045	79.6021		
F6	CREB1 Patient - 15	19.36857	19.36857	79.6021		
F7	CREB1 Patient - 16	20.07578	20.07578	79.00589		
F8	CREB1 Patient - 17	19.70654	19.70654	79.00589		
F9	CREB1 Patient - 18	20.61508	20.61508	79.44768		
F10	CREB1 Patient - 19	19.63701	19.63701	79.44768		
F11	CREB1 Patient - 20	19.30509	19.30509	79.44802		
F12	Sample 1	Undetermined	35.85719	67.51765	72.28979	80.64105
G1	ABL1 NC1	21.68826	21.68826	88.25319		
G2	ABL1 cell line	Undetermined		89.74432	66.92992	83.77977
G3	ABL1 NC2	17.83112	17.83112	88.10162		
G4	ABL1 Patient -1	20.17124	20.17124	87.95251		
G5	ABL1 Patient -2	19.25721	19.25721	88.10097		
G6	ABL1 Patient -3	18.94114	18.94114	88.10097		
G7	ABL1 Patient -4	19.82673	19.82673	87.65543		
G8	ABL1 Patient -5	18.75846	18.75846	87.65543		
G9	ABL1 Patient -6	20.48543	20.48543	87.94765		
G10	ABL1 Patient -7	19.19609	19.19609	87.94765		
G11	ABL1 Patient -8	18.9044	18.9044	87.94841		
G12	ABL1 Patient -9	21.19981	21.19981	87.94841		
H1	ABL1 Patient -10	20.52981	20.52981	88.4023		
H2	ABL1 Patient -11	19.62924	19.62924	88.25319		
H3	ABL1 Patient -12	21.27447	21.27447	88.10162		
H4	ABL1 Patient -13	20.57599	20.57599	88.10162		
H5	ABL1 Patient -14	19.77819	19.77819	88.25006		
H6	ABL1 Patient -15	19.41604	19.41604	88.25006		
H7	ABL1 Patient -16	19.12897	19.12897	87.65543		
H8	ABL1 Patient -17	18.96672	18.96672	87.5063		
H9	ABL1 Patient -18	22.09544	22.09544	87.94765		
H10	ABL1 Patient -19	21.69682	21.69682	87.94765		
H11	ABL1 Patient -20	21.89518	21.89518	87.94841		
H12	Sample 1	Undetermined	35.85719	68.85982		

- Plate 2

Well	Sample Name	Target Name	Cт	Cт Mean	Tm1	Tm2	Tm3
A1	NC	GSK 3 alpha	Undetermined		67.67398	62.15508	
A2	Cells	GSK 3 alpha	18.22849	18.22849	82.58995		
A3	PC	GSK 3 alpha	17.94518	17.94518	87.50805	82.43793	
A4	Sample 21	GSK 3 alpha	Undetermined		66.92934		
A5	Sample 22	GSK 3 alpha	18.3085	18.3085	82.58856		
A6	Sample 23	GSK 3 alpha	16.85506	16.85506	87.65908	82.29029	
A7	Sample 24	GSK 3 alpha	18.46113	18.46113	81.99258		
A8	Sample 25	GSK 3 alpha	18.4752	18.4752	81.69437		
A9	Sample 26	GSK 3 alpha	18.2099	18.2099	82.43517		
A10	Sample 27	GSK 3 alpha	18.94322	18.94322	82.43517		
A11	Sample 28	GSK 3 alpha	18.15656	18.15656	82.58567		
A12	Sample 29	GSK 3 alpha	18.25063	18.25063	82.28739	87.20894	
B1	Sample 30	GSK 3 alpha	18.30921	18.30921	82.58995	87.66138	
B2	Sample 31	GSK 3 alpha	17.30285	17.30285	87.66138	82.58995	
B3	Sample 32	GSK 3 alpha	16.38246	16.38246	87.50805	82.13969	
B4	Sample 33	GSK 3 alpha	18.2303	18.2303	82.43793		
B5	Sample 34	GSK 3 alpha	18.16128	18.16128	82.58856		

B6	Sample 35	GSK 3 alpha	17.41797	17.41797	87.50995	82.29029	
B7	Sample 36	GSK 3 alpha	19.27167	19.27167	81.84348		
B8	Sample 37	GSK 3 alpha	19.23364	19.23364	81.84348		
B9	Sample 38	GSK 3 alpha	18.41569	18.41569	82.13692	87.20721	
B10	Sample 39	GSK 3 alpha	18.29101	18.29101	82.28604	87.20721	
B11	Sample 40	GSK 3 alpha	18.95022	18.95022	82.43653		
B12	Sample 41	GSK 3 alpha	18.88678	18.88678	87.20894	82.13825	
C1	NC	GSK 3 Beta	Undetermined		82.88827	75.57944	90.34625
C2	Cells	GSK 3 Beta	17.83371	17.83371	83.18658		
C3	PC	GSK 3 Beta	18.56511	18.56511	82.8853		
C4	Sample 21	GSK 3 Beta	Undetermined		87.35893	65.13989	68.12231
C5	Sample 22	GSK 3 Beta	18.51269	18.51269	82.88683		
C6	Sample 23	GSK 3 Beta	17.68488	17.68488	82.88683		
C7	Sample 24	GSK 3 Beta	17.14776	17.14776	82.29079		
C8	Sample 25	GSK 3 Beta	17.71351	17.71351	82.29079		
C9	Sample 26	GSK 3 Beta	17.43985	17.43985	82.73342		
C10	Sample 27	GSK 3 Beta	18.27199	18.27199	82.73342		
C11	Sample 28	GSK 3 Beta	17.9783	17.9783	82.7348		
C12	Sample 29	GSK 3 Beta	18.96816	18.96816	82.7348		

D1	Sample 30	GSK 3 Beta	18.53387	18.53387	83.03743		
D2	Sample 31	GSK 3 Beta	18.057	18.057	83.03743		
D3	Sample 32	GSK 3 Beta	17.88759	17.88759	82.73618		
D4	Sample 33	GSK 3 Beta	17.53528	17.53528	82.8853		
D5	Sample 34	GSK 3 Beta	16.99841	16.99841	82.88683		
D6	Sample 35	GSK 3 Beta	18.87291	18.87291	82.88683		
D7	Sample 36	GSK 3 Beta	17.49886	17.49886	82.29079		
D8	Sample 37	GSK 3 Beta	18.29896	18.29896	82.29079		
D9	Sample 38	GSK 3 Beta	17.50967	17.50967	82.73342		
D10	Sample 39	GSK 3 Beta	18.46126	18.46126	82.73342		
D11	Sample 40	GSK 3 Beta	18.44798	18.44798	82.7348		
D12	Sample 41	GSK 3 Beta	18.8804	18.8804	82.7348		
E1	NC	CREB1	Undetermined		77.07104	66.92818	
E2	Cells	CREB1	17.94048	17.94048	79.75591		
E3	PC	CREB1	17.96267	17.96267	79.45551		
E4	Sample 21	CREB1	Undetermined		65.28902	75.57837	88.25365
E5	Sample 22	CREB1	17.80426	17.80426	79.6059		
E6	Sample 23	CREB1	16.85059	16.85059	79.6059		
E7	Sample 24	CREB1	18.15388	18.15388	79.01053		
E8	Sample 25	CREB1	17.96041	17.96041	79.01053		

E9	Sample 26	CREB1	17.45397	17.45397	79.45264		
E10	Sample 27	CREB1	18.64828	18.64828	79.45264		
E11	Sample 28	CREB1	18.07394	18.07394	79.45377		
E12	Sample 29	CREB1	18.24938	18.24938	79.45377		
F1	Sample 30	CREB1	18.21295	18.21295	79.75591		
F2	Sample 31	CREB1	17.33432	17.33432	79.75591		
F3	Sample 32	CREB1	16.94877	16.94877	79.45551		
F4	Sample 33	CREB1	17.60763	17.60763	79.45551		
F5	Sample 34	CREB1	17.36808	17.36808	79.6059		
F6	Sample 35	CREB1	17.80283	17.80283	79.6059		
F7	Sample 36	CREB1	18.24807	18.24807	79.15963		
F8	Sample 37	CREB1	19.33056	19.33056	79.01053		
F9	Sample 38	CREB1	18.57754	18.57754	79.45264		
F10	Sample 39	CREB1	18.36801	18.36801	79.45264		
F11	Sample 40	CREB1	18.74956	18.74956	79.45377		
F12	Sample 41	CREB1	18.46012	18.46012	79.45377		
G1	NC	ABL1	Undetermined		69.01642		
G2	Cells	ABL1	16.93228	16.93228	88.25801		
G3	PC	ABL1	17.68644	17.68644	88.10454		
G4	Sample 21	ABL1	Undetermined		65.73637	70.65737	92.8764

G5	Sample 22	ABL1	16.71461	16.71461	88.25562		
G6	Sample 23	ABL1	16.40455	16.40455	88.25562		
G7	Sample 24	ABL1	20.45025	20.45025	87.65848		
G8	Sample 25	ABL1	18.39088	18.39088	87.65848		
G9	Sample 26	ABL1	18.29044	18.29044	87.95285		
G10	Sample 27	ABL1	18.94639	18.94639	87.95285		
G11	Sample 28	ABL1	20.31246	20.31246	87.95463		
G12	Sample 29	ABL1	18.20011	18.20011	87.95463		
H1	Sample 30	ABL1	18.38076	18.38076	88.40718		
H2	Sample 31	ABL1	17.27541	17.27541	88.40718		
H3	Sample 32	ABL1	16.35228	16.35228	88.10454		
H4	Sample 33	ABL1	19.52015	19.52015	88.10454		
H5	Sample 34	ABL1	20.02355	20.02355	88.25562		
H6	Sample 35	ABL1	16.68155	16.68155	88.25562		
H7	Sample 36	ABL1	20.75109	20.75109	87.65848		
H8	Sample 37	ABL1	17.70359	17.70359	87.65848		
H9	Sample 38	ABL1	18.21176	18.21176	87.95285		
H10	Sample 39	ABL1	18.37708	18.37708	87.95285		
H11	Sample 40	ABL1	20.84068	20.84068	88.10377		

H12	Sample 41	ABL1	19.17607	19.17607	88.10377		

- Plate 3

Well	Sample Name	Cт	Cт Mean	Tm1	Tm2	Tm3
A1	GSK 3 alpha NC1	17.72357559	17.72357559	82.58686066	87.65673065	
A2	GSK 3 alpha Cell line					
A3	GSK 3 alpha Healthy person	16.6445179	16.6445179	82.28623962		
A4	GSK 3 alpha Patient - 1	17.34978294	17.34978294	82.43535614	87.35606384	
A5	GSK 3 Alpha Patient - 2	17.32799149	17.32799149	82.28594971	87.35544586	
A6	GSK 3 Alpha Patient - 3	16.25360107	16.25360107	87.50454712		
A7	GSK 3 Alpha Patient - 4	18.5051136	18.5051136	81.69023132		
A8	GSK 3 Alpha Patient - 5	18.00955009	18.00955009	81.69023132	86.61151886	
A9	GSK 3 Alpha Patient - 6	18.17056084	18.17056084	82.28099823	87.35115814	
A10	GSK 3 Alpha Patient - 7	16.55882454	16.55882454	82.28099823	87.35115814	
A11	GSK 3 Alpha Patient - 8	16.35250854	16.35250854	87.20275879	81.98322296	
A12	GSK 3 Alpha Patient - 9	18.1298275	18.1298275	82.43061066		
B1	GSK 3 Alpha Patient - 10	18.3124733	18.3124733	82.73597717		
B2	GSK 3 Alpha Patient - 11	18.17155457	18.17155457	87.65673065	82.43774414	
B3	GSK 3 Alpha Patient - 12	17.21609306	17.21609306	82.13713074	87.35606384	
B4	GSK 3 Alpha Patient - 13	17.45670891	17.45670891	82.28623962	87.35606384	
B5	GSK 3 Alpha Patient - 14	Undetermined		87.35544586	82.13684082	
B6	GSK 3 Alpha Patient - 15	17.35715675	17.35715675	82.43505096		
B7	GSK 3 Alpha Patient - 16	17.58639717	17.58639717	81.83935547		
B8	GSK 3 Alpha Patient - 17	16.0920372	16.0920372	86.61151886	81.39196777	
B9	GSK 3 Alpha Patient - 18	17.78026199	17.78026199	82.43012238		
B10	GSK 3 Alpha Patient - 19	17.41620445	17.41620445	82.13188171		
B11	GSK 3 Alpha Patient - 20	17.64873505	17.64873505	82.28147888		
B12	Sample 1	33.53114319	33.53114319	86.75537109	68.8598175	

C1	GSK 3 Beta NC1	17.50579262	17.50579262	83.03420258		
C2	GSK 3 Beta Cell line	Undetermined		83.63066101	72.29801178	87.95496
C3	GSK 3 Beta NC2	16.35326385	16.35326385	82.88269043		
C4	GSK 3 Beta Patient - 1	17.66467857	17.66467857	82.73358154		
C5	GSK 3 Beta Patient - 2	17.96616554	17.96616554	82.88235474		
C6	GSK 3 Beta Patient - 3	16.10005188	16.10005188	82.73325348		
C7	GSK 3 Beta Patient - 4	16.78033829	16.78033829	82.13761902		
C8	GSK 3 Beta Patient - 5	17.47484589	17.47484589	82.13761902		
C9	GSK 3 Beta Patient - 6	18.66955566	18.66955566	82.72836304		
C10	GSK 3 Beta Patient - 7	15.91600609	15.91600609	82.72836304		
C11	GSK 3 Beta Patient - 8	16.17996979	16.17996979	82.57974243		
C12	GSK 3 Beta Patient - 9	17.86086464	17.86086464	82.72886658		
D1	GSK 3 Beta Patient - 10	17.8614254	17.8614254	83.03420258		
D2	GSK 3 Beta Patient - 11	19.57947922	19.57947922	83.03420258		
D3	GSK 3 Beta Patient - 12	17.28634834	17.28634834	82.73358154		
D4	GSK 3 Beta Patient - 13	16.16944885	16.16944885	82.73358154		
D5	GSK 3 Beta Patient - 14	16.78938866	16.78938866	82.88235474		
D6	GSK 3 Beta Patient - 15	16.74996185	16.74996185	82.88235474		
D7	GSK 3 Beta Patient - 16	17.84086609	17.84086609	82.28675079		
D8	GSK 3 Beta Patient - 17	16.70256996	16.70256996	82.13761902		
D9	GSK 3 Beta Patient - 18	16.87101555	16.87101555	82.72836304		
D10	GSK 3 Beta Patient - 19	16.86209679	16.86209679	82.72836304		
D11	GSK 3 Beta Patient - 20	16.71029282	16.71029282	82.72886658		
D12	Sample 1	Undetermined	33.53114319	69.60546875	73.78109741	78.70237
E1	CREB1 NC1	17.15900993	17.15900993	79.75370026		
E2	CREB1 Cell line	Undetermined		65.88612366	70.95599365	79.30636
E3	CREB1 NC2	16.69983101	16.69983101	79.45310974		
E4	CREB1 Patient - 1	16.67643356	16.67643356	79.45310974		
E5	CREB1 Patient - 2	16.93855858	16.93855858	79.45298767		
E6	CREB1 Patinet - 3	15.79999638	15.79999638	79.45298767		

E7	CREB1 Patient - 4	16.98731422	16.98731422	79.00588989		
E8	CREB1 Patient - 5	16.82817841	16.82817841	79.00588989		
E9	CREB1 Patient - 6	18.16943359	18.16943359	79.44767761		
E10	CREB1 Patient - 7	15.62963772	15.62963772	79.44767761		
E11	CREB1 Patient - 8	15.53638649	15.53638649	79.29888916		
E12	CREB1 Patient - 9	18.23518181	18.23518181	79.44802094		
F1	CREB1 Patient - 10	18.24063301	18.24063301	79.75370026		
F2	CREB1 Patient - 11	18.81741524	18.81741524	79.75370026		
F3	CREB1 Patient - 12	17.5317173	17.5317173	79.45310974		
F4	CREB1 Patient - 13	17.53935051	17.53935051	79.45310974		
F5	CREB1 Patient - 14	16.89041901	16.89041901	79.60209656		
F6	CREB1 Patient - 15	17.00984383	17.00984383	79.60209656		
F7	CREB1 Patient - 16	17.60095406	17.60095406	79.00588989		
F8	CREB1 Patient - 17	16.14772415	16.14772415	79.00588989		
F9	CREB1 Patient - 18	17.98271561	17.98271561	79.44767761		
F10	CREB1 Patient - 19	17.22580147	17.22580147	79.44767761		
F11	CREB1 Patient - 20	16.88423729	16.88423729	79.44802094		
F12	Sample 1	Undetermined	33.53114319	67.51765442	72.28979492	80.64105
G1	ABL1 NC1	19.12765121	19.12765121	88.25318909		
G2	ABL1 cell line	Undetermined		89.74432373	66.92991638	83.77977
G3	ABL1 NC2	15.76301384	15.76301384	88.10162354		
G4	ABL1 Patient -1	17.18518639	17.18518639	87.95251465		
G5	ABL1 Patient -2	16.38106537	16.38106537	88.10096741		
G6	ABL1 Patient -3	15.43328953	15.43328953	88.10096741		
G7	ABL1 Patient -4	17.35513687	17.35513687	87.65542603		
G8	ABL1 Patient -5	16.3166008	16.3166008	87.65542603		
G9	ABL1 Patient -6	17.73335838	17.73335838	87.94764709		
G10	ABL1 Patient -7	15.5773983	15.5773983	87.94764709		
G11	ABL1 Patient -8	15.52987671	15.52987671	87.94841003		
G12	ABL1 Patient -9	18.60337448	18.60337448	87.94841003		

H1	ABL1 Patient -10	18.21222115	18.21222115	88.40229797		
H2	ABL1 Patient -11	17.7889843	17.7889843	88.25318909		
H3	ABL1 Patient -12	18.89070129	18.89070129	88.10162354		
H4	ABL1 Patient -13	17.84443474	17.84443474	88.10162354		
H5	ABL1 Patient -14	16.71042633	16.71042633	88.25006104		
H6	ABL1 Patient -15	17.14895439	17.14895439	88.25006104		
H7	ABL1 Patient -16	16.70449448	16.70449448	87.65542603		
H8	ABL1 Patient -17	16.05904388	16.05904388	87.50630188		
H9	ABL1 Patient -18	19.65167618	19.65167618	87.94764709		
H10	ABL1 Patient -19	19.32387733	19.32387733	87.94764709		
H11	ABL1 Patient -20	19.49708748	19.49708748	87.94841003		
H12	Sample 1	Undetermined	33.53114319	68.8598175		

- Plate 4

Well	Sample Name	Target Name	C_T	C_T Mean	Tm1	Tm2	Tm3
A1	NC	GSK 3 Alpha	Undetermined		67.08057404	61.26535797	
A2	SUBP15 Cell	GSK 3 Alpha	18.22483444	18.22483444	82.73693085		
A3	PC	GSK 3 Alpha	18.04395866	18.04395866	82.58789063	87.65692902	
A4	Sample 41	GSK 3 Alpha	16.62866592	16.62866592	82.289711	87.35874939	
A5	Sample 42	GSK 3 Alpha	17.15641022	17.15641022	82.43791199		
A6	Sample 43	GSK 3 Alpha	16.7959671	16.7959671	82.43791199		
A7	Sample 44	GSK 3 Alpha	16.36217117	16.36217117	81.84308624		
A8	Sample 45	GSK 3 Alpha	17.87303543	17.87303543	81.69399261		
A9	Sample 46	GSK 3 Alpha	17.78946114	17.78946114	82.43347168		
A10	Sample 47	GSK 3 Alpha	16.2616806	16.2616806	82.28438568		
A11	Sample 48	GSK 3 Alpha	16.85199738	16.85199738	82.28656006		
A12	Sample 49	GSK 3 Alpha	17.31097794	17.31097794	82.28656006		
B1	Sample 50	GSK 3 Alpha	17.69099426	17.69099426	82.58782196	87.65750122	

B2	Sample 51	GSK 3 Alpha	16.82757759	16.82757759	82.73693085		
B3	Sample 52	GSK 3 Alpha	17.45860481	17.45860481	82.43880463		
B4	Sample 53	GSK 3 Alpha	17.13713455	17.13713455	82.43880463	87.20965576	
B5	Sample 54	GSK 3 Alpha	16.75138855	16.75138855	82.28881836		
B6	Sample 55	GSK 3 Alpha	17.4751091	17.4751091	82.43791199		
B7	Sample 56	GSK 3 Alpha	16.85403442	16.85403442	81.69399261		
B8	Sample 57	GSK 3 Alpha	16.77684212	16.77684212	81.84308624		
B9	Sample 58	GSK 3 Alpha	16.24910545	16.24910545	82.28438568		
B10	Sample 59	GSK 3 Alpha	17.49435997	17.49435997	82.28438568		
B11	Sample 60	GSK 3 Alpha	16.23412132	16.23412132	82.28656006		
B12		GSK 3 Alpha	Undetermined		65.73431396		
C1	NC	GSK 3 Beta	Undetermined		71.85203552	88.40304565	68.2734375
C2	SUBP15 Cell	GSK 3 Beta	17.81488228	17.81488228	83.18425751		
C3	PC	GSK 3 Beta	18.80684471	18.80684471	83.03515625		
C4	Sample 41	GSK 3 Beta	17.97717476	17.97717476	82.58789063		
C5	Sample 42	GSK 3 Beta	17.95894814	17.95894814	82.73610687		
C6	Sample 43	GSK 3 Beta	18.41752052	18.41752052	82.73610687		
C7	Sample 44	GSK 3 Beta	18.26765442	18.26765442	82.14128113		
C8	Sample 45	GSK 3 Beta	18.55891991	18.55891991	81.9921875		
C9	Sample 46	GSK 3 Beta	18.68675041	18.68675041	82.58256531		
C10	Sample 47	GSK 3 Beta	16.85057259	16.85057259	82.58256531		
C11	Sample 48	GSK 3 Beta	17.96516609	17.96516609	82.58479309		
C12	Sample 49	GSK 3 Beta	17.7923069	17.7923069	82.58479309		
D1	Sample 50	GSK 3 Beta	19.26649475	19.26649475	83.03514862		
D2	Sample 51	GSK 3 Beta	18.32047081	18.32047081	83.03514862		
D3	Sample 52	GSK 3 Beta	18.69469833	18.69469833	82.73698425		
D4	Sample 53	GSK 3 Beta	19.35908508	19.35908508	82.73698425		
D5	Sample 54	GSK 3 Beta	17.18002701	17.18002701	82.73610687		
D6	Sample 55	GSK 3 Beta	18.63948441	18.63948441	82.73610687		
D7	Sample 56	GSK 3 Beta	17.21987534	17.21987534	82.14128113		

D8	Sample 57	GSK 3 Beta	17.73935509	17.73935509	82.14128113		
D9	Sample 58	GSK 3 Beta	16.77157211	16.77157211	82.58256531		
D10	Sample 59	GSK 3 Beta	17.94911385	17.94911385	82.58256531		
D11	Sample 60	GSK 3 Beta	16.19785881	16.19785881	82.58479309		
D12		GSK 3 Beta	Undetermined		90.78636169	73.33940125	83.92687225
E1	NC	CREB 1	Undetermined		65.14216614	75.43063354	78.56190491
E2	SUBP15 Cell	CREB 1	17.78864861	17.78864861	79.75476837		
E3	PC	CREB 1	18.00692749	18.00692749	79.45701599		
E4	Sample 41	CREB 1	17.15355301	17.15355301	79.15883636		
E5	Sample 42	CREB 1	17.19884872	17.19884872	79.30686951		
E6	Sample 43	CREB 1	17.3548851	17.3548851	79.30686951		
E7	Sample 44	CREB 1	16.72959137	16.72959137	78.86112976		
E8	Sample 45	CREB 1	17.89625359	17.89625359	78.71203613		
E9	Sample 46	CREB 1	17.5519352	17.5519352	79.15342712		
E10	Sample 47	CREB 1	17.608675	17.608675	79.15342712		
E11	Sample 48	CREB 1	16.95628738	16.95628738	79.15505219		
E12	Sample 49	CREB 1	17.71698761	17.71698761	79.15505219		
F1	Sample 50	CREB 1	18.17512512	18.17512512	79.60565948		
F2	Sample 51	CREB 1	17.84958458	17.84958458	79.60565948		
F3	Sample 52	CREB 1	18.15585518	18.15585518	79.30792999		
F4	Sample 53	CREB 1	17.3924427	17.3924427	79.30792999		
F5	Sample 54	CREB 1	16.83101273	16.83101273	79.30686951		
F6	Sample 55	CREB 1	17.89903831	17.89903831	79.45596313		
F7	Sample 56	CREB 1	17.32027626	17.32027626	78.86112976		
F8	Sample 57	CREB 1	17.03900337	17.03900337	78.86112976		
F9	Sample 58	CREB 1	16.21716309	16.21716309	79.15342712		
F10	Sample 59	CREB 1	17.50788116	17.50788116	79.30252075		
F11	Sample 60	CREB 1	16.66605377	16.66605377	79.3041687		
F12		CREB 1	Undetermined		68.120224		
G1	NC	ABL1	Undetermined		68.12432861		

G2	SUBP15 Cell	ABL1	16.9069767	16.9069767	88.40304565		
G3	PC	ABL1	17.61581802	17.61581802	88.25328064		
G4	Sample 41	ABL1	16.26274109	16.26274109	87.95510864		
G5	Sample 42	ABL1	17.49113655	17.49113655	88.10361481		
G6	Sample 43	ABL1	15.46016693	15.46016693	88.10361481		
G7	Sample 44	ABL1	17.05069351	17.05069351	87.65790558		
G8	Sample 45	ABL1	18.23213768	18.23213768	87.50880432		
G9	Sample 46	ABL1	15.52803612	15.52803612	87.94992065		
G10	Sample 47	ABL1	16.26794815	16.26794815	87.94992065		
G11	Sample 48	ABL1	17.32248878	17.32248878	87.95309448		
G12	Sample 49	ABL1	17.36175728	17.36175728	87.95309448		
H1	Sample 50	ABL1	17.51229095	17.51229095	88.40304565		
H2	Sample 51	ABL1	17.53509331	17.53509331	88.25393677		
H3	Sample 52	ABL1	17.56101608	17.56101608	88.10419464		
H4	Sample 53	ABL1	17.50268936	17.50268936	88.10419464		
H5	Sample 54	ABL1	16.66413689	16.66413689	88.25271606		
H6	Sample 55	ABL1	16.48507309	16.48507309	88.25271606		
H7	Sample 56	ABL1	17.12325668	17.12325668	87.65790558		
H8	Sample 57	ABL1	16.53444099	16.53444099	87.65790558		
H9	Sample 58	ABL1	15.6299305	15.6299305	87.94992065		
H10	Sample 59	ABL1	18.26931763	18.26931763	87.94992065		
H11	Sample 60	ABL1	16.12748909	16.12748909	88.102211		
H12		ABL1	Undetermined		67.97110748	92.72491455	82.88303375

- Plate 5

Well	Sample Name	Target Name	C_T	C_T Mean	C_T SD	Tm1	Tm2	Tm3
A1	NC	GSK 3 Alpha	Undetermined	19.77321	2.107449	61.87661	68.28442	90.78627
A2	SUBP15 Cell	GSK 3 Alpha	18.22304	19.77321	2.107449	82.59023		

A3	PC	GSK 3 Alpha	18.6543	19.77321	2.107449	82.58688		
A4	Sample 81	GSK 3 Alpha	18.94346	19.77321	2.107449	82.43786		
A5	Sample 82	GSK 3 Alpha	19.47272	19.77321	2.107449	82.43564	87.35313	
A6	Sample 83	GSK 3 Alpha	26.77121	19.77321	2.107449	87.50215		
A7	Sample 84	GSK 3 Alpha	19.26929	19.77321	2.107449	81.84274		
A8	Sample 85	GSK 3 Alpha	19.0751	19.77321	2.107449	81.99176		
A9	Sample 86	GSK 3 Alpha	18.89271	19.77321	2.107449	82.28641		
A10	Sample 87	GSK 3 Alpha	18.73591	19.77321	2.107449	82.43542		
A11	Sample 88	GSK 3 Alpha	19.24286	19.77321	2.107449	82.2868	87.20531	
A12	Sample 89	GSK 3 Alpha	19.41353	19.77321	2.107449	82.43584		
B1	Sample 90	GSK 3 Alpha	18.97531	19.77321	2.107449	82.73925		
B2	Sample 91	GSK 3 Alpha	19.20559	19.77321	2.107449	82.73925		
B3	Sample 92	GSK 3 Alpha	18.70845	19.77321	2.107449	82.43786		
B4	Sample 93	GSK 3 Alpha	18.72774	19.77321	2.107449	82.43786		
B5	Sample 94	GSK 3 Alpha	19.33961	19.77321	2.107449	82.28662	87.35313	
B6	Sample 95	GSK 3 Alpha	19.51336	19.77321	2.107449	82.43564		
B7	Sample 96	GSK 3 Alpha	19.02712	19.77321	2.107449	81.84274		
B8	Sample 97	GSK 3 Alpha	20.18877	19.77321	2.107449	81.84274		
B9	Sample 98	GSK 3 Alpha	19.98087	19.77321	2.107449	82.43542		
B10	Sample 99	GSK 3 Alpha	18.75938	19.77321	2.107449	82.28641		
B11	Sample 100	GSK 3 Alpha	19.36794	19.77321	2.107449	82.2868		
B12		GSK 3 Alpha	19.76953	19.77321	2.107449	82.2868		
C1	NC	GSK 3 Beta	Undetermined	19.77321	2.107449	87.80589	67.83736	73.20204
C2	SUBP15 Cell	GSK 3 Beta	18.44596	19.77321	2.107449	83.18631		
C3	PC	GSK 3 Beta	18.57215	19.77321	2.107449	82.8849		
C4	Sample 81	GSK 3 Beta	18.65359	19.77321	2.107449	82.8849		
C5	Sample 82	GSK 3 Beta	20.06183	19.77321	2.107449	82.88268		
C6	Sample 83	GSK 3 Beta	35.32173	19.77321	2.107449	82.73367		
C7	Sample 84	GSK 3 Beta	19.94723	19.77321	2.107449	82.28979		
C8	Sample 85	GSK 3 Beta	19.48131	19.77321	2.107449	82.28979		

C9	Sample 86	GSK 3 Beta	20.44383	19.77321	2.107449	82.73347		
C10	Sample 87	GSK 3 Beta	18.6968	19.77321	2.107449	82.73347		
C11	Sample 88	GSK 3 Beta	20.80706	19.77321	2.107449	82.73393		
C12	Sample 89	GSK 3 Beta	19.35603	19.77321	2.107449	82.73393		
D1	Sample 90	GSK 3 Beta	19.42546	19.77321	2.107449	83.18631		
D2	Sample 91	GSK 3 Beta	19.19791	19.77321	2.107449	83.03729		
D3	Sample 92	GSK 3 Beta	18.82647	19.77321	2.107449	82.73589		
D4	Sample 93	GSK 3 Beta	18.68045	19.77321	2.107449	82.73589		
D5	Sample 94	GSK 3 Beta	20.69844	19.77321	2.107449	82.73367		
D6	Sample 95	GSK 3 Beta	19.92037	19.77321	2.107449	82.88268		
D7	Sample 96	GSK 3 Beta	20.3495	19.77321	2.107449	82.28979		
D8	Sample 97	GSK 3 Beta	20.71186	19.77321	2.107449	82.28979		
D9	Sample 98	GSK 3 Beta	20.28796	19.77321	2.107449	82.73347		
D10	Sample 99	GSK 3 Beta	18.95746	19.77321	2.107449	82.73347		
D11	Sample 100	GSK 3 Beta	20.0589	19.77321	2.107449	82.73393		
D12		GSK 3 Beta	19.97824	19.77321	2.107449	82.73393		
E1	NC	CREB 1	Undetermined	19.77321	2.107449	71.56284	83.48434	76.7785
E2	SUBP15 Cell	CREB 1	18.89597	19.77321	2.107449	79.60986		
E3	PC	CREB 1	18.49335	19.77321	2.107449	79.30849		
E4	Sample 81	CREB 1	18.63357	19.77321	2.107449	79.30849		
E5	Sample 82	CREB 1	19.89177	19.77321	2.107449	79.45534		
E6	Sample 83	CREB 1	23.81991	19.77321	2.107449	79.45534	66.04398	
E7	Sample 84	CREB 1	19.737	19.77321	2.107449	78.86236		
E8	Sample 85	CREB 1	19.83284	19.77321	2.107449	78.86236		
E9	Sample 86	CREB 1	19.6096	19.77321	2.107449	79.30595		
E10	Sample 87	CREB 1	19.62432	19.77321	2.107449	79.30595		
E11	Sample 88	CREB 1	20.26319	19.77321	2.107449	79.30588		
E12	Sample 89	CREB 1	20.08136	19.77321	2.107449	79.30588		
F1	Sample 90	CREB 1	19.41001	19.77321	2.107449	79.75887		
F2	Sample 91	CREB 1	19.13034	19.77321	2.107449	79.60986		

F3	Sample 92	CREB 1	19.76609	19.77321	2.107449	79.45751		
F4	Sample 93	CREB 1	19.28983	19.77321	2.107449	79.45751		
F5	Sample 94	CREB 1	20.23696	19.77321	2.107449	79.45534		
F6	Sample 95	CREB 1	19.5801	19.77321	2.107449	79.45534		
F7	Sample 96	CREB 1	19.94271	19.77321	2.107449	79.01138		
F8	Sample 97	CREB 1	20.64466	19.77321	2.107449	79.01138		
F9	Sample 98	CREB 1	19.55291	19.77321	2.107449	79.30595		
F10	Sample 99	CREB 1	19.91109	19.77321	2.107449	79.30595		
F11	Sample 100	CREB 1	19.53271	19.77321	2.107449	79.30588		
F12		CREB 1	19.95496	19.77321	2.107449	79.30588		
G1	NC	ABL1	Undetermined	19.77321	2.107449	74.24518		
G2	SUBP15 Cell	ABL1	16.7313	19.77321	2.107449	88.25295		
G3	PC	ABL1	20.64419	19.77321	2.107449	87.95149		
G4	Sample 81	ABL1	20.50168	19.77321	2.107449	87.95149		
G5	Sample 82	ABL1	18.99021	19.77321	2.107449	88.09821		
G6	Sample 83	ABL1	25.42995	19.77321	2.107449	88.09821		
G7	Sample 84	ABL1	18.62506	19.77321	2.107449	87.65447		
G8	Sample 85	ABL1	18.41625	19.77321	2.107449	87.65447		
G9	Sample 86	ABL1	18.97611	19.77321	2.107449	87.94925		
G10	Sample 87	ABL1	20.35896	19.77321	2.107449	87.94925		
G11	Sample 88	ABL1	19.33378	19.77321	2.107449	87.95055		
G12	Sample 89	ABL1	18.80918	19.77321	2.107449	87.95055		
H1	Sample 90	ABL1	18.15885	19.77321	2.107449	88.40197		
H2	Sample 91	ABL1	20.6443	19.77321	2.107449	88.25295		
H3	Sample 92	ABL1	19.26476	19.77321	2.107449	88.10051		
H4	Sample 93	ABL1	18.03825	19.77321	2.107449	88.10051		
H5	Sample 94	ABL1	19.42577	19.77321	2.107449	88.09821		
H6	Sample 95	ABL1	19.94034	19.77321	2.107449	88.09821		
H7	Sample 96	ABL1	18.08213	19.77321	2.107449	87.65447		
H8	Sample 97	ABL1	21.24058	19.77321	2.107449	87.65447		

H9	Sample 98	ABL1	17.8614	19.77321	2.107449	87.94925		
H10	Sample 99	ABL1	18.77332	19.77321	2.107449	87.94925		
H11	Sample 100	ABL1	19.96562	19.77321	2.107449	87.95055		
H12		ABL1	19.95	19.77321	2.107449	87.95055		

- ALL controls

Well	Sample Name	Target Name	Cт	Cт Mean	Cт SD	Tm1	Tm2	Tm3
A1	Cell SUBP - 15	gsk 3 ALPHA	16.21977	16.17092	0.069094	82.58974		
A2	PC	gsk 3 ALPHA	13.8247	13.8247		87.36192	67.82578	73.49275
A3	HC1	gsk 3 ALPHA	16.50889	16.38423	0.176301	82.43974		
A4	HC2	gsk 3 ALPHA	Undetermined			68.57161	80.79942	89.59749
A5	HC3	gsk 3 ALPHA	16.36324	16.279	0.119131	82.43594		
A6	HC4	gsk 3 ALPHA	12.79426	12.24677	0.77428	61.56435	87.80293	76.17446
A7	NC	gsk 3 ALPHA	16.52492	16.43378	0.128892	81.84014		
B1	Cell SUBP - 15	gsk 3 ALPHA	16.12206	16.17092	0.069094	82.58974		
B2	PC	gsk 3 ALPHA	Undetermined	13.8247		89.15149	62.60619	81.24756
B3	HC1	gsk 3 ALPHA	16.25957	16.38423	0.176301	82.43974		
B4	HC2	gsk 3 ALPHA	Undetermined			92.729	61.56298	77.36967
B5	HC3	gsk 3 ALPHA	16.19476	16.279	0.119131	82.43594		
B6	HC4	gsk 3 ALPHA	11.69927	12.24677	0.77428	87.65384	81.98869	71.25473

B7	NC	gsk 3 ALPHA	16.34264	16.43378	0.128892	81.84014		
C1	Cell SUBP - 15	gsk 3 bETA	16.3318	16.28691	0.063489	83.18626		
C2	PC	gsk 3 bETA	Undetermined	18.27236		61.71141		
C3	HC1	gsk 3 bETA	16.30235	16.31724	0.02105	82.8871		
C4	HC2	gsk 3 bETA	15.65843	15.65843		90.93956	87.06245	66.18569
C5	HC3	gsk 3 bETA	16.2665	16.25191	0.020638	82.88319		
C6	HC4	gsk 3 bETA	Undetermined	14.21551		82.13778	93.31898	61.26618
C7	NC	gsk 3 bETA	16.1344	16.15553	0.029882	82.28743		
D1	Cell SUBP - 15	gsk 3 bETA	16.24202	16.28691	0.063489	83.18626		
D2	PC	gsk 3 bETA	18.27236	18.27236		68.27317	91.68672	76.47537
D3	HC1	gsk 3 bETA	16.33212	16.31724	0.02105	82.8871		
D4	HC2	gsk 3 bETA	Undetermined	15.65843		87.50981	91.83428	80.65031
D5	HC3	gsk 3 bETA	16.23731	16.25191	0.020638	82.88319		
D6	HC4	gsk 3 bETA	14.21551	14.21551		68.8694	93.02083	80.94511
D7	NC	gsk 3 bETA	16.17666	16.15553	0.029882	82.28743		
E1	Cell SUBP - 15	creb 1	16.65283	16.63975	0.018495	79.75625		
E2	PC	creb 1	12.72049	15.46712	3.88433	68.27317	80.5019	76.47537
E3	HC1	creb 1	16.24019	16.20454	0.050416	79.45734		
E4	HC2	creb 1	Undetermined			65.73833	87.65893	82.1415
E5	HC3	creb 1	16.42731	16.41766	0.013654	79.45428		
E6	HC4	creb 1	Undetermined			66.63316	90.93367	62.45884
E7	NC	creb 1	16.48057	16.58065	0.141542	78.85822		
F1	Cell SUBP - 15	creb 1	16.62667	16.63975	0.018495	79.75625		
F2	PC	creb 1	18.21376	15.46712	3.88433	63.50098	68.27317	90.04628
F3	HC1	creb 1	16.16889	16.20454	0.050416	79.45734		
F4	HC2	creb 1	Undetermined			70.51016	64.6945	61.26474
F5	HC3	creb 1	16.408	16.41766	0.013654	79.45428		

F6	HC4	creb 1	Undetermined			77.21805	67.37858	84.97035
F7	NC	creb 1	16.68074	16.58065	0.141542	79.00731		
G1	Cell SUBP - 15	abl 1	16.1803	15.77475	0.573529	88.25671		
G2	PC	abl 1	14.15367	15.494	1.895516	89.74802	61.56228	74.08927
G3	HC1	abl 1	18.30938	18.32685	0.024708	87.95717		
G4	HC2	abl 1	Undetermined	13.24038		92.13252	80.79942	76.92231
G5	HC3	abl 1	17.78949	17.84186	0.074071	87.95201		
G6	HC4	abl 1	12.33682	13.7151	1.949188	88.54834	61.56435	66.63316
G7	NC	abl 1	18.41091	18.54683	0.192219	87.50579		
H1	Cell SUBP - 15	abl 1	15.36921	15.77475	0.573529	88.25671		
H2	PC	abl 1	16.83433	15.494	1.895516	92.28325	80.80016	76.32623
H3	HC1	abl 1	18.34432	18.32685	0.024708	87.95717		
H4	HC2	abl 1	13.24038	13.24038		90.79044	70.95752	61.56298
H5	HC3	abl 1	17.89424	17.84186	0.074071	88.10109		
H6	HC4	abl 1	15.09338	13.7151	1.949188	61.41526	80.05061	85.86485
H7	NC	abl 1	18.68275	18.54683	0.192219	87.65489		

Table S8 : Statistical test done for validation by using GRAPHPAD Prism v.9 using Kruskal–Wallis for the RT-qPCR study

- Densitometric raw reading for the blot and loading control

Gene	Kruskal–Wallis	Comparison	Adjusted p-value	Significant?
GSK3α	$H(2) = 23.34$, $p < 0.0001$	Control vs. Cell line	< 0.0001	Yes (****)
GSK3α		Control vs. Patients	0.0017	Yes (**)
GSK3α		Cell line vs. Patients	0.0040	Yes (**)
GSK3β	$H(2) = 14.88$, $p = 0.0006$	Control vs. Cell line	> 0.9999	No (ns)
GSK3β		Control vs. Patients	0.0038	Yes (**)
GSK3β		Cell line vs. Patients	0.0700	No (ns)
CREB1	$H(2) = 18.56$, $p < 0.0001$	Control vs. Cell line	< 0.0001	Yes (****)
CREB1		Control vs. Patients	0.0053	Yes (**)
CREB1		Cell line vs. Patients	0.0148	Yes (*)

Table S9: Densitometric quantification of CREB1 and GSK3α/β Western blot signals across seven membranes. Raw densitometric band-volume measurements for the target protein and β-actin loading control are shown for each lane/sample across seven blot membranes. For each membrane, one Control sample and the corresponding Patient samples are identified by group. Normalized expression was calculated as the target protein band volume divided by the corresponding β-actin band volume. The normalized expression values were subsequently transformed to log_2 values for distributional analysis. Data are presented separately for CREB1 and GSK3α/β and include the membrane number, lane/sample identifier, raw target-protein band volume, β-actin band volume, experimental group, normalized expression, and log_2-transformed normalized expression.

CREB1						
**Membrane No.**	**Lane No.**	**Protein Band Vol.**	**Bet actin Band Vol.**	**Group**	**Norm. Expression**	**Log_2 (Norm.)**
1	Control 1	8208.00	34462816.00	Control	0.0002	-12.0357
1	Sample 1	148256.00	55363049.00	Patient	0.0027	-8.5447
1	Sample 2	182288.00	25783583.00	Patient	0.0071	-7.1441
1	Sample 3	20272.00	26724701.00	Patient	0.0008	-10.3645
1	Sample 4	192528.00	19538046.00	Patient	0.0099	-6.6651
1	Sample 5	114160.00	52532726.00	Patient	0.0022	-8.8460
1	Sample 6	197200.00	56965212.00	Patient	0.0035	-8.1743
1	Sample 7	182320.00	75331052.00	Patient	0.0024	-8.6906
1	Sample 8	136512.00	86124619.00	Patient	0.0016	-9.3013
2	Control 2	201201.00	25927924.00	Control	0.0078	-7.0097
2	Sample 9	590163.00	24620794.00	Patient	0.0240	-5.3826
2	Sample 10	223104.00	18273079.00	Patient	0.0122	-6.3559
2	Sample 11	902895.00	12872519.00	Patient	0.0701	-3.8336
2	Sample 12	427812.00	7636706.00	Patient	0.0560	-4.1579
2	Sample 13	520170.00	15345781.00	Patient	0.0339	-4.8827
2	Sample 14	303492.00	12368367.00	Patient	0.0245	-5.3489
2	Sample 15	696738.00	10999340.00	Patient	0.0633	-3.9807
3	Control 3	1103520.00	24231680.00	Control	0.0455	-4.4567
3	Sample 16	1986660.00	22808960.00	Patient	0.0871	-3.5212
3	Sample 17	2186460.00	27641480.00	Patient	0.0791	-3.6602
3	Sample 18	3185310.00	26398560.00	Patient	0.1207	-3.0510
3	Sample 19	2056500.00	21709640.00	Patient	0.0947	-3.4001
3	Sample 20	1368960.00	9637880.00	Patient	0.1420	-2.8156
3	Sample 21	1187640.00	11417200.00	Patient	0.1040	-3.2650
3	Sample 22	1630740.00	21494840.00	Patient	0.0759	-3.7204

3	Sample 23	1507920.00	35025320.00	Patient	0.0431	-4.5378
4	Control 4	2298047.00	7334200.00	Control	0.3133	-1.6742
4	Sample 24	1634817.00	2719600.00	Patient	0.6011	-0.7343
4	Sample 25	3309944.00	5321200.00	Patient	0.6220	-0.6849
4	Sample 26	1168671.00	1681400.00	Patient	0.6951	-0.5248
4	Sample 27	1657437.00	2573520.00	Patient	0.6440	-0.6348
4	Sample 28	4087028.00	5168480.00	Patient	0.7908	-0.3387
4	Sample 29	1600887.00	11346320.00	Patient	0.1411	-2.8253
4	Sample 30	1166525.00	1935280.00	Patient	0.6028	-0.7303
5	Control 5	4376126.00	1294840.00	Control	3.3797	1.7569
5	Sample 31	4067552.00	1471280.00	Patient	2.7646	1.4671
5	Sample 32	4557984.00	1797160.00	Patient	2.5362	1.3427
5	Sample 33	6344490.00	2314040.00	Patient	2.7417	1.4551
5	Sample 34	3097432.00	915840.00	Patient	3.3821	1.7579
5	Sample 35	5341822.00	1965600.00	Patient	2.7177	1.4424
5	Sample 36	8059896.00	1894880.00	Patient	4.2535	2.0887
5	Sample 37	7222496.00	2845480.00	Patient	2.5382	1.3438
5	Sample 38	9121340.00	6980480.00	Patient	1.3067	0.3859
6	Control 6	1668038.00	1493917.00	Control	1.1166	0.1591
6	Sample 39	2527600.00	990150.00	Patient	2.5527	1.3520
6	Sample 40	4094178.00	1059522.00	Patient	3.8642	1.9502
6	Sample 41	4709791.00	1201505.00	Patient	3.9199	1.9708
6	Sample 42	2922671.00	996259.00	Patient	2.9336	1.5527
6	Sample 43	3160212.00	574779.00	Patient	5.4981	2.4589
6	Sample 44	1673912.00	296512.00	Patient	5.6453	2.4971
6	Sample 45	703100.00	667029.00	Patient	1.0541	0.0760
7	Control 7	2418690.00	2264955.00	Control	1.0679	0.0947
7	Sample 46	9266650.00	6475148.00	Patient	1.4311	0.5171
7	Sample 47	9391340.00	5607609.00	Patient	1.6747	0.7439
7	Sample 48	8188655.00	5227508.00	Patient	1.5665	0.6475
7	Sample 49	9341020.00	6008171.00	Patient	1.5547	0.6367
7	Sample 50	6941385.00	4338583.00	Patient	1.5999	0.6780

GSK3α/β						
**Membrane No.**	**Lane No.**	**Protein Band Vol.**	**Beta actin Band Vol.**	**Group**	**Norm. Expression**	**Log$_2$ (Norm.)**
1	Control 1	35925.00	5717832.00	Control	0.0063	-7.3143

1	Sample 1	12750.00	7152951.00	Patient	0.0018	-9.1319
1	Sample 2	27650.00	6185549.00	Patient	0.0045	-7.8055
1	Sample 3	78350.00	10032365.00	Patient	0.0078	-7.0005
1	Sample 4	646475.00	5436447.00	Patient	0.1189	-3.0720
1	Sample 5	55700.00	6056271.00	Patient	0.0092	-6.7646
1	Sample 6	1122750.00	5606906.00	Patient	0.2002	-2.3202
1	Sample 7	3054300.00	5774257.00	Patient	0.5290	-0.9188
1	Sample 8	3523075.00	3554553.00	Patient	0.9911	-0.0128
2	Control 2	391240.00	7426782.00	Control	0.0527	-4.2466
2	Sample 9	263560.00	5349212.00	Patient	0.0493	-4.3431
2	Sample 10	221860.00	7600736.00	Patient	0.0292	-5.0984
2	Sample 11	601260.00	4859778.00	Patient	0.1237	-3.0148
2	Sample 12	402060.00	9027326.00	Patient	0.0445	-4.4888
2	Sample 13	450920.00	6861778.00	Patient	0.0657	-3.9276
2	Sample 14	438660.00	10478248.00	Patient	0.0419	-4.5782
2	Sample 15	2630700.00	9440200.00	Patient	0.2787	-1.8434
3	Control 3	3124909.00	1498406.00	Control	2.0855	1.0604
3	Sample 16	4039993.00	2140606.00	Patient	1.8873	0.9163
3	Sample 17	6248671.00	1241578.00	Patient	5.0328	2.3314
3	Sample 18	5949970.00	966784.00	Patient	6.1544	2.6216
3	Sample 19	3483180.00	6696950.00	Patient	0.5201	-0.9431
3	Sample 20	3527654.00	3021538.00	Patient	1.1675	0.2234
3	Sample 21	2046285.00	4883996.00	Patient	0.4190	-1.2551
3	Sample 22	3143668.00	4154956.00	Patient	0.7566	-0.4024
3	Sample 23	4912934.00	1495416.00	Patient	3.2853	1.7160
4	Control 4	4151496.00	14644644.00	Control	0.2835	-1.8187
4	Sample 24	6067713.00	15081360.00	Patient	0.4023	-1.3135
4	Sample 25	6097192.00	13560876.00	Patient	0.4496	-1.1532
4	Sample 26	6582099.00	18796708.00	Patient	0.3502	-1.5139
4	Sample 27	1200275.00	16527896.00	Patient	0.0726	-3.7835

4	Sample 28	6413507.00	13660052.00	Patient	0.4695	-1.0908
4	Sample 29	3064504.00	14354340.00	Patient	0.2135	-2.2278
4	Sample 30	3074713.00	13013476.00	Patient	0.2363	-2.0815
5	Control 5	6493651.00	3835975.00	Control	1.6928	0.7594
5	Sample 31	7454247.00	3850997.00	Patient	1.9357	0.9528
5	Sample 32	7462889.00	3443035.00	Patient	2.1675	1.1161
5	Sample 33	8423108.00	4076623.00	Patient	2.0662	1.0470
5	Sample 34	7495282.00	3392530.00	Patient	2.2093	1.1436
5	Sample 35	4443032.00	2308245.00	Patient	1.9249	0.9447
5	Sample 36	4056230.00	1817403.00	Patient	2.2319	1.1583
5	Sample 37	7173034.00	2274279.00	Patient	3.1540	1.6572
5	Sample 38	4412205.00	1856068.00	Patient	2.3772	1.2493
6	Control 6	2527379.00	1169892.00	Control	2.1604	1.1113
6	Sample 39	2405376.00	819576.00	Patient	2.9349	1.5533
6	Sample 40	1086949.00	631548.00	Patient	1.7211	0.7833
6	Sample 41	2961886.00	1096236.00	Patient	2.7019	1.4340
6	Sample 42	2002363.00	802908.00	Patient	2.4939	1.3184
6	Sample 43	7815471.00	2345472.00	Patient	3.3322	1.7365
6	Sample 44	4134066.00	1432116.00	Patient	2.8867	1.5294
6	Sample 45	1441880.00	299196.00	Patient	4.8192	2.2688
7	Control 7	9441705.00	8082138.00	Control	1.1682	0.2243
7	Sample 46	6856850.00	9733683.00	Patient	0.7044	-0.5054
7	Sample 47	12466272.00	7299112.00	Patient	1.7079	0.7722
7	Sample 48	21372624.00	10087532.00	Patient	2.1187	1.0832
7	Sample 49	4900259.00	6455417.00	Patient	0.7591	-0.3977
7	Sample 50	4434703.00	6595266.00	Patient	0.6724	-0.5726

Table S10. Shapiro–Wilk normality assessment of normalized Western blot measurements before and after $\log_2$ transformation. Normality was assessed separately for the Control and Patient groups for CREB1 and GSK3α/β using the Shapiro–Wilk test. Analyses were performed on the normalized protein/loading-control ratios before transformation and on the corresponding $\log_2$-transformed values. The table reports the sample size (n), Shapiro–Wilk W statistic, p-value, normality classification, and interpretation. Using $p < 0.05$ as the criterion for evidence against normality, CREB1 Control, CREB1 Patient, and GSK3α/β Patient showed evidence of non-normality before transformation, whereas GSK3α/β Control was consistent with normality. Following $\log_2$ transformation, CREB1 Control and GSK3α/β Control were consistent with normality, whereas CREB1 Patient and GSK3α/β Patient remained non-normal. The $\log_2$ transformation therefore did not result in normally distributed Patient-group values according to the Shapiro–Wilk test.

Shapiro-Wilk — Raw Normalized Values (Protein/GAPDH)					
**Group**	**n**	**W statistic**	**p-value**	**Normal?**	**Interpretation**
**CREB1 Control**	7	0.7527	0.013546	**No**	Significantly non-normal (p = 0.0135)
**CREB1 Patient**	50	0.7872	0.000000	**No**	Significantly non-normal (p = 0.0000)
**GSK3 Control**	7	0.8613	0.155454	Yes	Consistent with normality (p ≥ 0.05)
**GSK3 Patient**	50	0.8434	0.000010	**No**	Significantly non-normal (p = 0.0000)
Note: Performed on protein/GAPDH ratio before any transformation. Significant results indicate non-normality warranting log2 transformation.					
**Shapiro-Wilk — Log_2-Transformed Values**					
**Group**	**n**	**W statistic**	**p-value**	**Normal?**	**Interpretation**
**CREB1 Control**	7	0.9095	0.392320	Yes	Consistent with normality (p ≥ 0.05)
**CREB1 Patient**	50	0.9135	0.001388	**No**	Significantly non-normal (p = 0.0014)
**GSK3 Control**	7	0.8293	0.078801	Yes	Consistent with normality (p ≥ 0.05)
**GSK3 Patient**	50	0.8982	0.000419	**No**	Significantly non-normal (p = 0.0004)
Note: Log_2 transformation applied to reduce right-skew. Patient data remain significantly non-normal after transformation; this informed the choice of linear model with membrane blocking rather than a simple parametric t-test.					

Table S11: Linear model analysis of CREB1 and GSK3α/β protein expression. A linear model was used to evaluate differences between control and patient samples while accounting for between-membrane (blot) technical variability. For both CREB1 and GSK3α/β, the response variable was $\log_2$(Protein/GAPDH), and the fixed effects were Group (Control vs Patient) and Membrane No. (1–7), with Membrane No. treated as a blocking factor. The reference levels were Control for Group and membrane 1 for Membrane No. The analysis included 7 control and 50 patient observations (57 total).

For **CREB1**, the estimated Group effect for Patient versus Control was β = 1.3169 ± 0.3424 SE on the $\log_2$ scale (t = 3.8463, df = 49, p = 0.000347; 95% CI, 0.6289–2.0050), corresponding to a fold-change of 2.491. The model had R^2 = 0.9569 and adjusted R^2 = 0.9507.

For **GSK3α/β**, the estimated Group effect for Patient versus Control was β = 0.4670 ± 0.6357 SE on the $\log_2$ scale (t = 0.7346, df = 49, p = 0.466075; 95% CI, −0.8105–1.7445), corresponding to a fold-change of 1.382. The model had R^2 = 0.7354 and adjusted R^2 = 0.6976.

Observed, fitted, residual, and standardized residual values are shown for each sample. Residuals were calculated as observed − fitted value, and standardized residuals as residual divided by the SD of the residuals. Rows with |standardized residual| > 2 were highlighted as potential outliers.

Linear Model — CREB1						
**Model formula**	Log2Norm ~ C(Group) + C(Membrane_No)					
**Reference level**	Group = Control | Membrane = 1					
**Response variable**	$\log_2$(Protein/GAPDH)					
**Fixed effects**	Group (Control vs Patient) + Membrane No. (1–7)					
**Rationale**	Membrane No. treated as blocking factor to account for between-blot technical variance					
**n (Control)**	7					
**n (Patient)**	50					
**Total obs.**	57					
**Key Result — Group Effect (Patient vs Control)**						
**β (log2 scale)**	**SE**	**t-statistic**	**df (residual)**	**p-value**	**95% CI**	**Fold-change (2^β)**
1.3169	0.3424	3.8463	49	**0.000347**	[0.629, 2.005]	2.491
**Model Fit Statistics**						
**R^2**	**Adjusted R^2**	**AIC**	**BIC**	**F-statistic**	**F p-value**	**df model / df resid**
0.9569	0.9507	150.26	166.60	155.416	0.000000	7 / 49
**Full Model Coefficients (all parameters)**						

Parameter	Coefficient (β)	Std. Error	t-statistic	p-value	CI lower (95%)	CI upper (95%)
Intercept	-10.0335	0.4152	-24.1640	0.000000	-10.8680	-9.1991
C(Group, Treatment(reference='Control'))[T.Patient]	1.3169	0.3424	3.8463	0.000347	0.6289	2.0050
C(Membrane_No)[T.2]	3.7622	0.4118	9.1364	0.000000	2.9347	4.5897
C(Membrane_No)[T.3]	5.2598	0.3995	13.1673	0.000000	4.4571	6.0626
C(Membrane_No)[T.4]	7.8628	0.4118	19.0946	0.000000	7.0353	8.6903
C(Membrane_No)[T.5]	10.3118	0.3995	25.8145	0.000000	9.5091	11.1146
C(Membrane_No)[T.6]	10.3833	0.4118	25.2155	0.000000	9.5558	11.2108
C(Membrane_No)[T.7]	9.4891	0.4470	21.2276	0.000000	8.5908	10.3874

Observed | Fitted | Residual Values (per sample)

Membrane	Lane	Group	Log_2 Observed	Log_2 Fitted	Residual	Std. Residual
1	Control 1	Control	-12.0357	-10.0335	-2.0022	-2.526
1	Sample 1	Patient	-8.5447	-8.7166	0.1719	0.217
1	Sample 2	Patient	-7.1441	-8.7166	1.5725	1.984
1	Sample 3	Patient	-10.3645	-8.7166	-1.6479	-2.079
1	Sample 4	Patient	-6.6651	-8.7166	2.0515	2.588
1	Sample 5	Patient	-8.8460	-8.7166	-0.1294	-0.163
1	Sample 6	Patient	-8.1743	-8.7166	0.5423	0.684
1	Sample 7	Patient	-8.6906	-8.7166	0.0260	0.033
1	Sample 8	Patient	-9.3013	-8.7166	-0.5847	-0.738
2	Control 2	Control	-7.0097	-6.2713	-0.7384	-0.932
2	Sample 9	Patient	-5.3826	-4.9544	-0.4282	-0.540
2	Sample 10	Patient	-6.3559	-4.9544	-1.4015	-1.768
2	Sample 11	Patient	-3.8336	-4.9544	1.1208	1.414
2	Sample 12	Patient	-4.1579	-4.9544	0.7965	1.005
2	Sample 13	Patient	-4.8827	-4.9544	0.0717	0.090
2	Sample 14	Patient	-5.3489	-4.9544	-0.3945	-0.498
2	Sample 15	Patient	-3.9807	-4.9544	0.9737	1.228
3	Control 3	Control	-4.4567	-4.7737	0.3170	0.400
3	Sample 16	Patient	-3.5212	-3.4568	-0.0644	-0.081
3	Sample 17	Patient	-3.6602	-3.4568	-0.2034	-0.257
3	Sample 18	Patient	-3.0510	-3.4568	0.4058	0.512
3	Sample 19	Patient	-3.4001	-3.4568	0.0567	0.072
3	Sample 20	Patient	-2.8156	-3.4568	0.6411	0.809
3	Sample 21	Patient	-3.2650	-3.4568	0.1917	0.242
3	Sample 22	Patient	-3.7204	-3.4568	-0.2636	-0.333
3	Sample 23	Patient	-4.5378	-3.4568	-1.0810	-1.364

4	Control 4	Control	-1.6742	-2.1707	0.4965	0.626
4	Sample 24	Patient	-0.7343	-0.8538	0.1195	0.151
4	Sample 25	Patient	-0.6849	-0.8538	0.1689	0.213
4	Sample 26	Patient	-0.5248	-0.8538	0.3290	0.415
4	Sample 27	Patient	-0.6348	-0.8538	0.2190	0.276
4	Sample 28	Patient	-0.3387	-0.8538	0.5151	0.650
4	Sample 29	Patient	-2.8253	-0.8538	-1.9715	-2.487
4	Sample 30	Patient	-0.7303	-0.8538	0.1235	0.156
5	Control 5	Control	1.7569	0.2783	1.4786	1.865
5	Sample 31	Patient	1.4671	1.5953	-0.1282	-0.162
5	Sample 32	Patient	1.3427	1.5953	-0.2526	-0.319
5	Sample 33	Patient	1.4551	1.5953	-0.1402	-0.177
5	Sample 34	Patient	1.7579	1.5953	0.1626	0.205
5	Sample 35	Patient	1.4424	1.5953	-0.1529	-0.193
5	Sample 36	Patient	2.0887	1.5953	0.4934	0.622
5	Sample 37	Patient	1.3438	1.5953	-0.2514	-0.317
5	Sample 38	Patient	0.3859	1.5953	-1.2093	-1.526
6	Control 6	Control	0.1591	0.3498	-0.1907	-0.241
6	Sample 39	Patient	1.3520	1.6667	-0.3147	-0.397
6	Sample 40	Patient	1.9502	1.6667	0.2834	0.358
6	Sample 41	Patient	1.9708	1.6667	0.3041	0.384
6	Sample 42	Patient	1.5527	1.6667	-0.1140	-0.144
6	Sample 43	Patient	2.4589	1.6667	0.7922	0.999
6	Sample 44	Patient	2.4971	1.6667	0.8303	1.048
6	Sample 45	Patient	0.0760	1.6667	-1.5907	-2.007
7	Control 7	Control	0.0947	-0.5445	0.6392	0.806
7	Sample 46	Patient	0.5171	0.7725	-0.2554	-0.322
7	Sample 47	Patient	0.7439	0.7725	-0.0285	-0.036
7	Sample 48	Patient	0.6475	0.7725	-0.1250	-0.158
7	Sample 49	Patient	0.6367	0.7725	-0.1358	-0.171
7	Sample 50	Patient	0.6780	0.7725	-0.0945	-0.119

Linear Model — GSK3α/β	
**Model formula**	Log2Norm ~ C(Group) + C(Membrane_No)
**Reference level**	Group = Control | Membrane = 1
**Response variable**	Log_2(Protein/GAPDH)

**Fixed effects**	Group (Control vs Patient) + Membrane No. (1–7)
**Rationale**	Membrane No. treated as blocking factor to account for between-blot technical variance
**n (Control)**	7
**n (Patient)**	50
**Total obs.**	57

**Key Result — Group Effect (Patient vs Control)**						
**β (log2 scale)**	**SE**	**t-statistic**	**df (residual)**	**p-value**	**95% CI**	**Fold-change (2^β)**
0.4670	0.6357	0.7346	49	**0.466075**	[-0.81, 1.744]	1.382

**Model Fit Statistics**						
**R^2**	**Adjusted R^2**	**AIC**	**BIC**	**F-statistic**	**F p-value**	**df model / df resid**
0.7354	0.6976	220.80	237.14	19.455	0.000000	7 / 49

**Full Model Coefficients (all parameters)**						
**Parameter**	**Coefficient (β)**	**Std. Error**	**t-statistic**	**p-value**	**CI lower (95%)**	**CI upper (95%)**
Intercept	-5.3418	0.7709	-6.9292	0.000000	-6.8911	-3.7926
C(Group, Treatment(reference='Control'))[T.Patient]	0.4670	0.6357	0.7346	0.466075	-0.8105	1.7445
C(Membrane_No)[T.2]	0.9906	0.7645	1.2957	0.201145	-0.5458	2.5270
C(Membrane_No)[T.3]	5.6233	0.7416	7.5821	0.000000	4.1329	7.1136
C(Membrane_No)[T.4]	3.0604	0.7645	4.0030	0.000211	1.5240	4.5967
C(Membrane_No)[T.5]	6.0410	0.7416	8.1454	0.000000	4.5506	7.5314
C(Membrane_No)[T.6]	6.4001	0.7645	8.3713	0.000000	4.8637	7.9365
C(Membrane_No)[T.7]	5.0534	0.8299	6.0888	0.000000	3.3855	6.7212

Observed | Fitted | Residual Values (per sample)

Membrane	Lane	Group	Log_2 Observed	Log_2 Fitted	Residual	Std. Residual
1	Control 1	Control	-7.3143	-5.3418	-1.9725	-1.340
1	Sample 1	Patient	-9.1319	-4.8748	-4.2570	-2.893
1	Sample 2	Patient	-7.8055	-4.8748	-2.9306	-1.991
1	Sample 3	Patient	-7.0005	-4.8748	-2.1257	-1.444
1	Sample 4	Patient	-3.0720	-4.8748	1.8029	1.225
1	Sample 5	Patient	-6.7646	-4.8748	-1.8898	-1.284
1	Sample 6	Patient	-2.3202	-4.8748	2.5547	1.736
1	Sample 7	Patient	-0.9188	-4.8748	3.9561	2.688
1	Sample 8	Patient	-0.0128	-4.8748	4.8620	3.304
2	Control 2	Control	-4.2466	-4.3512	0.1046	0.071
2	Sample 9	Patient	-4.3431	-3.8842	-0.4589	-0.312
2	Sample 10	Patient	-5.0984	-3.8842	-1.2142	-0.825
2	Sample 11	Patient	-3.0148	-3.8842	0.8694	0.591
2	Sample 12	Patient	-4.4888	-3.8842	-0.6046	-0.411
2	Sample 13	Patient	-3.9276	-3.8842	-0.0434	-0.029
2	Sample 14	Patient	-4.5782	-3.8842	-0.6939	-0.472
2	Sample 15	Patient	-1.8434	-3.8842	2.0409	1.387
3	Control 3	Control	1.0604	0.2814	0.7790	0.529
3	Sample 16	Patient	0.9163	0.7484	0.1679	0.114
3	Sample 17	Patient	2.3314	0.7484	1.5830	1.076
3	Sample 18	Patient	2.6216	0.7484	1.8732	1.273
3	Sample 19	Patient	-0.9431	0.7484	-1.6915	-1.149
3	Sample 20	Patient	0.2234	0.7484	-0.5250	-0.357
3	Sample 21	Patient	-1.2551	0.7484	-2.0035	-1.361
3	Sample 22	Patient	-0.4024	0.7484	-1.1508	-0.782
3	Sample 23	Patient	1.7160	0.7484	0.9676	0.658
4	Control 4	Control	-1.8187	-2.2815	0.4628	0.314
4	Sample 24	Patient	-1.3135	-1.8145	0.5009	0.340
4	Sample 25	Patient	-1.1532	-1.8145	0.6612	0.449

4	Sample 26	Patient	-1.5139	-1.8145	0.3006	0.204
4	Sample 27	Patient	-3.7835	-1.8145	-1.9690	-1.338
4	Sample 28	Patient	-1.0908	-1.8145	0.7237	0.492
4	Sample 29	Patient	-2.2278	-1.8145	-0.4133	-0.281
4	Sample 30	Patient	-2.0815	-1.8145	-0.2670	-0.181
5	Control 5	Control	0.7594	0.6992	0.0603	0.041
5	Sample 31	Patient	0.9528	1.1661	-0.2133	-0.145
5	Sample 32	Patient	1.1161	1.1661	-0.0501	-0.034
5	Sample 33	Patient	1.0470	1.1661	-0.1192	-0.081
5	Sample 34	Patient	1.1436	1.1661	-0.0225	-0.015
5	Sample 35	Patient	0.9447	1.1661	-0.2214	-0.150
5	Sample 36	Patient	1.1583	1.1661	-0.0079	-0.005
5	Sample 37	Patient	1.6572	1.1661	0.4910	0.334
5	Sample 38	Patient	1.2493	1.1661	0.0831	0.056
6	Control 6	Control	1.1113	1.0582	0.0530	0.036
6	Sample 39	Patient	1.5533	1.5252	0.0281	0.019
6	Sample 40	Patient	0.7833	1.5252	-0.7419	-0.504
6	Sample 41	Patient	1.4340	1.5252	-0.0913	-0.062
6	Sample 42	Patient	1.3184	1.5252	-0.2068	-0.141
6	Sample 43	Patient	1.7365	1.5252	0.2112	0.144
6	Sample 44	Patient	1.5294	1.5252	0.0042	0.003
6	Sample 45	Patient	2.2688	1.5252	0.7436	0.505
7	Control 7	Control	0.2243	-0.2885	0.5128	0.348
7	Sample 46	Patient	-0.5054	0.1785	-0.6839	-0.465
7	Sample 47	Patient	0.7722	0.1785	0.5937	0.403
7	Sample 48	Patient	1.0832	0.1785	0.9047	0.615
7	Sample 49	Patient	-0.3977	0.1785	-0.5762	-0.392
7	Sample 50	Patient	-0.5726	0.1785	-0.7511	-0.510

Highlighted rows (orange) = |standardized residual| > 2 (potential outliers). Residuals = observed − fitted value. Std. residual = residual / SD(residuals).

Table S12: Statistical analysis and sensitivity analysis of CREB1 and GSK3α/β protein expression. The table summarizes normality testing, the primary linear-model analysis, residual diagnostics, and a Mann–Whitney U sensitivity analysis for log_2-transformed protein/GAPDH values. For the primary analysis, a linear model including Group (Control vs Patient) and Membrane No. as a blocking factor was used. For CREB1, the estimated Group effect (Patient vs Control) was β = 1.3169 (SE = 0.3424; t = 3.8463; df = 49; p = 0.000347; 95% CI, 0.629–2.005; fold-change = 2.491). For GSK3α/β, the estimated Group effect was β = 0.4670 (SE = 0.6357; t = 0.7346; df = 49; p = 0.466075; 95% CI, −0.81–1.744; fold-change = 1.382).

Normality was assessed using the Shapiro–Wilk test. For log_2-transformed values, CREB1 was normally distributed in controls (W = 0.9095, p = 0.392) but not in patients (W = 0.9135, p = 0.001388); GSK3α/β was normally distributed in controls (W = 0.8293, p = 0.079) but not in patients (W = 0.8982, p = 0.000419). Shapiro–Wilk testing of model residuals indicated non-normal residuals for both CREB1 (W = 0.9480, p = 0.0160) and GSK3α/β (W = 0.9350, p = 0.0043).

A two-sided Mann–Whitney U test was performed on the log_2-transformed values as a sensitivity analysis. For CREB1, the median was −1.6742 in controls and −0.7323 in patients (U = 150, p = 0.5592). For GSK3α/β, the median was 0.2243 in controls and −0.4539 in patients (U = 155, p = 0.6424). Neither Mann–Whitney U test was significant. The table notes that this sensitivity analysis does not account for membrane blocking and therefore may differ from the linear-model analysis where membrane variance is large.

- Descriptive statistics

Mann-Whitney U Test — Sensitivity Analysis (Log_2-Transformed Values)

Rationale: Mann-Whitney U (non-parametric) was run as a sensitivity analysis because log_2 patient values remain significantly non-normal after transformation (SW $p < 0.05$). This test does NOT account for membrane blocking and is therefore expected to differ from the linear model for proteins where membrane variance is large.

Protein	n Control	n Patient	Median Control (log2)	Median Patient (log2)	U statistic	p-value (two-sided)	Significant?
**CREB1**	7	50	-1.6742	-0.7323	150	0.5592	No
**GSK3α/β**	7	50	0.2243	-0.4539	155	0.6424	No

IMPORTANT — Discrepancy note: For CREB1, the linear model detects a highly significant group effect (p = 0.000347) while Mann-Whitney is non-significant (p = 0.559). This is expected and is NOT a contradiction. The linear model accounts for membrane blocking (R^2 = 0.957 for CREB1), removing the dominant source of variance. Mann-Whitney ignores this blocking structure, making it confounded by between-membrane variability and therefore underpowered. The linear model result is the primary analysis.

Residual Normality — Shapiro-Wilk on Model Residuals

CREB1

W statistic	p-value	Normal residuals?	Implication
0.9480	0.0160	No	Residuals are significantly non-normal ($p < 0.05$). Parametric inference remains approximately valid for $n > 30$ (central limit theorem), but Mann-Whitney was run as sensitivity analysis.

GSK3α/β

W statistic	p-value	Normal residuals?	Implication
0.9350	0.0043	No	Residuals are significantly non-normal ($p < 0.05$). Parametric inference remains approximately valid for $n > 30$ (central limit theorem), but Mann-Whitney was run as sensitivity analysis.

Complete Statistical Results Summary

A. Normality Testing (Shapiro-Wilk)							
Protein	Group	Stage	n	W	p-value	Normal?	Decision taken
CREB1	Control	Raw (Norm.	7	0.7527	0.013546	No	Log_2 transformation applied
CREB1	Patient	Raw (Norm.	50	0.7872	0.000001	No	Log_2 transformation applied
GSK3α/β	Control	Raw (Norm.	7	0.8613	0.155454	Yes	Log_2 transformation applied
GSK3α/β	Patient	Raw (Norm.	50	0.8434	0.00001	No	Log_2 transformation applied
CREB1	Control	Log_2	7	0.9095	0.392	Yes	Non-parametric test not required for controls
CREB1	Patient	Log_2	50	0.9135	0.001388	No	Mann-Whitney used as sensitivity analysis
GSK3α/β	Control	Log_2	7	0.8293	0.079	Yes	Non-parametric test not required for controls
GSK3α/β	Patient	Log_2	50	0.8982	0.000419	No	Mann-Whitney used as sensitivity analysis

B. Primary Analysis — Linear Model (Group + Membrane blocking)								
Protein	β	SE	t-stat	df resid	p-value	95% CI	Fold-change	Significant?
**CREB1**	1.3169	0.3424	3.8463	49	**0.000347**	[0.629, 2.005]	2.491	**Yes ✓**
**GSK3α/β**	0.4670	0.6357	0.7346	49	**0.466075**	[-0.81, 1.744]	1.382	**No**

C. Residual Diagnostics — Shapiro-Wilk on Model Residuals				
Protein	W (residuals)	p (residuals)	rmal residua	Implication
**CREB1**	0.9480	0.0160	**No**	Non-normal residuals; n=57 provides some robustness via CLT. Mann-Whitney run as non-parametric sensitivity analysis.
**GSK3α/β**	0.9350	0.0043	**No**	Non-normal residuals; n=57 provides some robustness via CLT. Mann-Whitney run as non-parametric sensitivity analysis.

D. Sensitivity Analysis — Mann-Whitney U (Log_2, two-sided)				
Protein	U statistic	p-value	Significant?	Agree with LM?
**CREB1**	150	0.5592	No	**membrane confound**
**GSK3α/β**	155	0.6424	No	**Yes**

Table S13. Descriptive statistics of normalized CREB1 and GSK3α/β Western blot measurements. Descriptive statistics are shown separately for the Control (n = 7) and Patient (n = 50) groups for CREB1 and GSK3α/β. For each group, the untransformed normalized values ("raw") and the corresponding $\log_2$-transformed values are summarized by the mean, standard deviation (SD), and median. The first and third quartiles (Q1 and Q3) are additionally reported for the $\log_2$-transformed values. The raw values represent the normalized protein measurements used for the subsequent $\log_2$ transformation. These descriptive statistics summarize the distribution of the observations and do not represent an inferential statistical comparison between groups.

- list of phosphorylated kinases and related signaling proteins analyzed using the **Human Phospho-Kinase Array (Proteome Profiler™, R&D Systems)**

Descriptive Statistics — CREB1									
Group	n	Mean (raw)	SD (raw)	Median (raw)	Mean (log2)	SD (log2)	Median (log2)	Q1 (log2)	Q3 (log2)
Control	7	0.8473	1.2168	0.3133	-3.3094	4.8839	-1.6742	-5.7332	0.1269
Patient	50	1.2139	1.5429	0.6019	-2.1951	3.6857	-0.7323	-4.4428	1.1930

Descriptive Statistics — GSK3α/β									
Group	n	Mean (raw)	SD (raw)	Median (raw)	Mean (log2)	SD (log2)	Median (log2)	Q1 (log2)	Q3 (log2)
Control	7	1.0642	0.9489	1.1682	-1.4606	3.2376	0.2243	-3.0326	0.9099
Patient	50	1.3766	1.4707	0.7305	-1.0401	2.8370	-0.4539	-2.2971	1.1367

Supplementary Table S14: This table presents the list of phosphorylated kinases and related signaling proteins analyzed using the **Human Phospho-Kinase Array (Proteome Profiler™, R&D Systems)**. Each row corresponds to a specific target protein along with its associated **phosphorylation site(s)**, representing key regulatory residues involved in cellular signaling pathways. Phosphorylation at these sites reflects activation or modulation of critical signaling pathways, including MAPK/ERK, PI3K/Akt, JAK/STAT, and stress-response pathways. Proteins such as **Akt, ERK1/2, JNK, p38α, STATs, and p53** are included due to their central roles in regulating cell proliferation, survival, apoptosis, and immune

responses.The data derived from this panel represent **relative phosphorylation levels**, typically quantified via chemiluminescent signal intensity, enabling comparative analysis between experimental conditions rather than absolute quantification.

S.No	Target/Control	Phosphorylation Site
1	Akt 1/2/3	T308
2	Akt 1/2/3	S473
3	CREB	S133
4	EGF R	Y1086
ss5	eNOS	S1177
6	ERK1/2	T202/Y204, T185/Y187
7	Chk-2	T68
8	c-Jun	S63
9	Fgr	Y412
10	GSK-3α/β	S21/S9
11	GSK-3β	S9
12	HSP27	S78/S82
13	p53	S15
14	p53	S46
15	p53	S392
16	JNK 1/2/3	T183/Y185, T221/Y223
17	Lck	Y394
18	Lyn	Y397
19	MSK1/2	S376/S360
20	p70 S6 Kinase	T389
21	p70 S6 Kinase	T421/S424
22	PRAS40	T246
23	p38α	T180/Y182
24	PDGF Rβ	Y751
25	PLC-γ1	Y783
26	Src	Y419
27	PYK2	Y402
28	RSK1/2	S221/S227
29	RSK1/2/3	S380/S386/S377
30	STAT2	(pY690)
31	STAT5a/b	Y694/Y699
32	WNK1	T60
33	Yes	Y426
34	STAT1	Y701
35	STAT3	Y705
36	STAT3	S727
37	STAT6	Y641

Supplementary Table S15: Raw densitometric values obtained from the Proteome Profiler™ Human Phospho-Kinase Array analysis of SUBP-15 cells following treatment with Perifosine, GDC-0941, GSK690693, or Rapamycin at their respective IC_{50} concentrations for 24 h. Pixel densities were quantified using Image Lab Software (Bio-Rad) from duplicate antibody spots on each membrane, with duplicate spot intensities averaged for each phosphoprotein. Data are presented for each of the two independent biological replicates per treatment group. Background subtraction was performed using PBS-loaded negative control spots, and membrane-specific positive reference spots were used for normalization. These raw densitometric values were used to calculate fold-change relative to untreated control samples and to generate the z-score-normalized heatmap presented in Figure 4.

- Patient characteristics for the 100 patients

Type	Control	Perifosine	GSK690693	GDC-0941	Rapamycin
Reference spot	4.37E-06	1.98778E-06	2.32939E-06	4.39985E-06	8.20872E-06
Reference spot	5.21085E-06	2.39514E-06	2.42584E-06	4.34598E-06	1.18096E-05
Reference spot	5.10279E-06	2.09487E-06	1.80023E-06	3.90814E-06	3.42585E-06
Reference spot	5.03916E-06	2.26324E-06	1.96388E-06	4.11463E-06	2.57608E-06
beta catenin	9.87888E-07	7.45066E-07	5.2809E-07	6.71052E-07	7.97506E-07
beta catenin	8.96138E-07	6.76957E-07	4.85519E-07	5.95317E-07	7.19366E-07
CREB	2.02006E-07	2.36429E-07	-3.17127E-08	2.88088E-07	8.20963E-07
CREB	1.22299E-07	2.77305E-07	-4.81429E-08	2.71826E-07	6.68121E-07
EGF R	2.16946E-07	2.31607E-07	3.63616E-07	1.76591E-07	3.95035E-07
EGF R	2.59849E-07	2.20726E-07	3.19675E-07	2.04173E-07	3.5448E-07
eNOS	1.92806E-07	1.91744E-07	-2.87645E-08	1.57078E-07	1.12756E-06
eNOS	1.96487E-07	1.7073E-07	4.84293E-08	1.36241E-07	1.40019E-06
ERK1/2	5.16907E-07	4.18998E-07	1.74872E-08	3.37934E-07	8.96585E-08
ERK1/2	5.46791E-07	5.06967E-07	1.1967E-07	4.16856E-07	4.55393E-07
Fgr	3.62594E-07	3.78353E-07	1.03861E-07	5.00165E-07	8.20403E-07
Fgr	3.70459E-07	2.52616E-07	4.86265E-08	4.29226E-07	8.77952E-07
GSK-3aplha/beta	4.44173E-07	2.99705E-07	5.29897E-08	2.7284E-07	2.75068E-07
GSK-3aplha/beta	3.24211E-07	3.04159E-07	4.62582E-08	1.84953E-07	-2.82837E-08
GSK-3beta	4.7777E-07	2.97978E-07	3.41599E-07	2.42944E-07	-1.38197E-07
GSK-3beta	3.52391E-07	3.02933E-07	7.90502E-08	1.7047E-07	-1.65732E-07
HSP27	6.84109E-08	1.7633E-07	7.45132E-08	7.37429E-08	1.64864E-06
HSP27	1.98219E-07	2.05074E-07	-1.97218E-09	1.17044E-07	1.74183E-06
Jnk1/2/3	5.67438E-07	6.23149E-07	4.05045E-08	5.16014E-07	8.99809E-07
Jnk1/2/3	4.43234E-07	3.89552E-07	4.26747E-08	5.22158E-07	8.63902E-07
Lck	3.36435E-07	1.61969E-07	1.54746E-07	2.63237E-07	8.04134E-07
Lck	4.76997E-07	2.26792E-07	7.32858E-08	2.52924E-07	8.46521E-07
Lyn	3.17742E-07	3.50202E-07	1.26332E-07	2.37654E-07	7.37094E-07

Lyn	2.19343E-07	3.84771E-07	8.92464E-08	1.04355E-07	7.60775E-07
MSK1/2	3.40165E-07	2.61833E-07	5.05425E-07	1.44716E-07	2.17372E-07
MSK1/2	2.39912E-07	2.61401E-07	1.37171E-07	2.21276E-07	2.6581E-07
p38alpha	4.22391E-07	2.67906E-07	1.81211E-07	3.77674E-07	1.38415E-06
p38alpha	2.82923E-07	3.00875E-07	1.59893E-07	3.82629E-07	1.35544E-06
PDGF Rbeta	5.12724E-07	3.89621E-07	4.91917E-07	5.09621E-07	1.6278E-06
PDGF Rbeta	4.68381E-07	4.17727E-07	5.26148E-07	4.9568E-07	1.76641E-06
PLC - Y1	1.01771E-06	6.69501E-07	1.09878E-08	4.8094E-07	2.13089E-06
PLC - Y1	8.93085E-07	6.64562E-07	-7.0838E-10	5.08231E-07	2.17584E-06
Src	7.42789E-07	7.05097E-07	9.47575E-08	5.27832E-07	1.5402E-07
Src	6.97961E-07	6.93685E-07	1.52377E-07	5.97931E-07	2.55496E-07
STAT 2	4.90477E-07	4.54755E-07	1.21485E-07	4.79137E-07	1.58271E-06
STAT 2	1.65107E-07	3.37358E-07	9.1371E-08	4.6073E-07	1.73702E-06
STAT 5a/b	4.37467E-07	4.37187E-07	6.05919E-08	5.56684E-07	1.06736E-06
STAT 5a/b	3.51992E-07	4.63493E-07	1.00667E-07	5.5353E-07	1.17086E-06
WNK 1	5.60126E-07	4.09519E-07	2.23393E-10	4.15886E-07	8.40413E-07
WNK 1	5.95644E-07	3.25523E-07	1.74872E-08	4.0637E-07	1.12788E-06
Yes	3.06842E-07	2.33971E-07	1.33959E-07	2.28341E-07	6.37727E-08
Yes	1.69597E-07	2.81148E-07	1.23296E-07	2.54577E-07	-2.66928E-08
STAT 1	-9.41853E-08	-1.57259E-08	4.18749E-08	-7.24573E-09	4.76804E-08
STAT 1	-1.86082E-08	-5.45185E-09	4.31476E-07	4.68886E-08	7.91839E-08
STAT 2	-3.0005E-08	3.45629E-09	2.21142E-07	-1.09076E-10	5.41195E-08
STAT 2	-5.35003E-08	-1.09575E-08	2.61504E-07	-4.38499E-08	1.44944E-09
STAT 3	2.64365E-08	-5.02322E-09	-1.58559E-08	3.16742E-08	-1.44829E-09
STAT 3	-5.01209E-09	-1.58727E-08	1.68645E-07	-2.35468E-08	6.29283E-08
Unknown	-7.71859E-08	-3.19968E-08	1.96872E-07	5.69608E-09	1.3504E-08
Unknown	4.37253E-08	-4.9151E-08	4.7368E-07	-2.2648E-08	3.79122E-08
PBS (Negtive Control)	-1.22465E-07	5.27761E-09	1.39431E-07	-9.95775E-09	4.03198E-08
PBS (Negtive Control)	1.66578E-08	3.75267E-09	1.59893E-07	3.14694E-08	7.12091E-08
Unknown	-1.18212E-07	-4.94988E-08	2.2351E-08	9.15264E-09	3.44387E-08
Unknown	6.8683E-08	-1.82136E-08	5.61264E-08	-2.39377E-09	3.83619E-08
Unknown	1.96554E-08	-1.79552E-08	1.43014E-07	-8.41272E-09	8.81124E-08
Unknown	-5.69486E-08	-2.75314E-08	8.23539E-08	4.02817E-08	8.80974E-08
Unknown	-2.30273E-08	-2.52412E-08	4.55568E-07	1.42284E-08	-5.04769E-08
Unknown	-4.22453E-08	-2.07493E-09	3.81233E-07	1.08411E-08	-4.98499E-08

Unknown	-9.44893E-08	4.1813E-09	3.23111E-07	-1.54603E-08	9.41612E-06
Unknown	1.35384E-08	-1.76488E-08	3.54597E-07	-1.36219E-08	9.11905E-06
STAT6	1.44456E-06	5.34335E-08	1.33577E-06	9.21847E-08	5.51577E-07
STAT6	5.89465E-07	-7.29494E-08	1.17801E-06	4.147E-08	4.36159E-07
hsp60	9.16159E-06	1.52052E-06	-1.7862E-07	1.16713E-06	5.70479E-06
hsp60	8.82922E-06	1.69372E-06	-1.36108E-07	1.14775E-06	5.71096E-06
Unknown	1.53093E-06	1.74038E-07	5.1978E-07	1.99682E-07	8.25467E-07
Unknown	1.34493E-06	1.89038E-07	3.21096E-07	2.36461E-07	7.78312E-07
Unknown	7.47494E-07	7.04617E-08	4.28045E-07	4.5187E-08	3.32024E-07
Unknown	6.01941E-07	1.19165E-07	3.70788E-07	1.01026E-07	3.02564E-07
PYK2	3.16385E-06	5.36978E-07	7.89544E-07	3.69519E-07	1.47421E-06
PYK2	2.59015E-06	4.3181E-07	6.4753E-07	3.96733E-07	1.61335E-06
RSK 1/2	1.08655E-06	1.38489E-07	7.18345E-08	2.58693E-08	3.89851E-07
RSK 1/2	7.5799E-07	1.76566E-07	8.67703E-08	1.33022E-07	6.13662E-07
p70 s6 Kinase	2.07191E-06	2.24329E-07	8.1666E-07	2.31078E-07	9.61357E-07
p70 s6 Kinase	1.37481E-06	2.09745E-07	4.96209E-07	2.05875E-07	8.64141E-07
p70 s6 Kinase	6.55141E-07	6.54399E-08	3.93245E-07	9.25681E-08	2.94071E-07
p70 s6 Kinase	4.04312E-07	1.23456E-07	3.92994E-07	1.09393E-07	4.64832E-07
p53	1.67668E-06	2.44858E-07	3.96941E-07	1.65628E-07	1.29713E-06
p53	1.55229E-06	2.08962E-07	1.9782E-07	2.17907E-07	1.50846E-06
p53	4.087E-06	7.78924E-07	1.68399E-07	8.31856E-07	3.24774E-06
p53	3.0148E-06	1.01215E-06	1.5573E-07	6.61959E-07	3.45456E-06
Chk-2	2.28079E-06	4.96707E-07	4.20347E-07	6.63156E-07	2.00633E-06
Chk-2	1.66576E-06	4.28472E-07	2.2945E-07	5.4649E-07	1.34059E-06
c-Jun	7.89768E-07	3.89718E-07	4.64884E-07	1.74323E-07	1.2381E-06
c-Jun	6.96765E-07	2.9514E-07	4.96843E-07	1.79511E-07	1.11563E-06
Akt1/2/3	1.6625E-06	6.89295E-07	-1.51605E-07	5.6578E-07	2.05247E-06
Akt1/2/3	1.40245E-06	5.70277E-07	-1.88179E-07	4.02935E-07	1.53341E-06
Akt1/2/3	8.89281E-07	3.5595E-07	2.53026E-07	1.22213E-07	1.34454E-06
Akt1/2/3	8.71153E-07	2.77352E-07	2.76194E-07	1.27121E-07	1.18958E-06
p53	7.47499E-07	7.60235E-08	1.83639E-07	1.52743E-07	8.82917E-07
p53	6.6272E-07	7.70071E-08	1.27497E-07	1.23325E-07	2.83861E-07
PRAS40	1.12466E-06	1.79524E-07	-4.34699E-09	1.66456E-07	1.10828E-06
PRAS40	9.8369E-07	1.70823E-07	-2.45198E-08	8.06699E-08	9.47021E-07
RSK 1/2/3	7.16236E-07	2.73989E-07	1.26695E-07	2.47814E-07	1.05504E-06
RSK 1/2/3	6.68797E-07	2.35556E-07	1.17256E-07	2.49677E-07	1.11172E-06
Unknown	3.62519E-06	6.4556E-07	1.6734E-07	7.44145E-07	2.41083E-06
Unknown	3.17606E-06	6.46653E-07	1.75714E-07	7.75638E-07	2.12345E-06
PBS (Negtive Control)	9.26878E-08	-1.84053E-08	-1.59822E-07	-3.81099E-09	9.3075E-09

PBS (Negtive Control)	1.31195E-08	9.37502E-09	-1.39502E-07	-1.77006E-08	-1.20836E-07
Reference spot	1.15792E-05	2.61864E-06	2.31578E-06	3.69962E-06	1.02602E-05
Reference spot	1.19823E-05	3.11533E-06	2.21283E-06	4.10348E-06	1.11159E-05

Table S16. Distribution of BCR::ABL1 transcript subtypes and clinical and molecular characteristics in patients with Philadelphia chromosome-positive B-cell acute lymphoblastic leukemia **(A)** Patient Number ID, (B) date of sample collection, (C) Age distribution of patients, (D) sex of the respective patients (E) RNA obtained after extraction from patient PBMNC, (F) Mortality status (G – I) Relative expression of **GSK3A, GSK3B, and CREB1**, respectively, compared with healthy controls.

1) Biological and technical replicates –

Patient No	date of sample collection	Age	Sex	RNA value	Transcript positive	Status	GSK 3 alpha expression compared to healthy control	GSK 3 Beta expression compared to healthy control	CREB1 expression compared to healthy control
Patient - 1	20-Jul-22	3	M	352	BCR::ABL1 (minor, p190)		0.219901	0.183151	0.305946
Patient - 2	14-Dec-22	23	F	419.7	BCR::ABL1 (minor, p190)		0.112841	0.048567	0.105137
Patient - 3	25-Jul-22	30	F	388.4	BCR::ABL1 (minor)		0.071454	0.000986	0.094166
Patient - 4	07-Sep-22	29	M	1979.2	BCR::ABL1 (minor)	Expired	0.172337	0.437409	0.308245
Patient - 5	17-Jun-22	12	M	454.7	BCR::ABL1 (minor)		0.058311	0.093458	0.124943
Patient - 6	03-Sep-22	4	M	144	BCR::ABL1 (minor, p190)		0.188185	0.122983	0.14496
Patient - 7	########	7	F	362.9	BCR::ABL1 (minor, p190)		0.112686	0.067228	0.158548
Patient - 8	########	4	M	293.2	BCR::ABL1 (major, p210)		0.124119	0.045139	0.1036
Patient - 9	11-Oct-22	16	M	1370.5	BCR::ABL1 (minor)		0.336807	0.393883	0.308907
Patient - 10	13-Oct-22	52	F	522.7	BCR::ABL1 (major, p210)		0.19492	0.276508	0.185463
Patient - 11	14-Nov-22	7.5	M	195.8	BCR::ABL1 (major, p210)		0.167699	0.087077	0.106741
Patient - 12	31-Oct-22	41	M	330	BCR::ABL1 (minor)		0.653082	0.709887	0.491805

Patient - 13	30-Sep-22	4	M	418.9	BCR::ABL1 (minor, p190)		0.292793	0.637582	0.278368
Patient - 14	24-Nov-22	2.5	M	392.4	BCR::ABL1 (minor, p190)		0.143115	0.096737	0.108378
Patient - 15	02-Dec-22	17	M	240.3	BCR::ABL1 (minor)		0.194435	0.342911	0.225918
Patient - 16	21-Mar-23	45	F	341.7	BCR::ABL1 (micro)		0.108867	0.100171	0.113408
Patient - 17	12-Jan-23	18	M	207.5	BCR::ABL1 (minor)		0.140863	0.051421	0.130904
Patient - 18	25-Apr-23	6	M	935.4	BCR::ABL1 (major, p210)		0.826537	1.787711	0.609949
Patient - 19	17-Jan-23	36	M	312.3	BCR::ABL1 (major, p210)		0.825347	1.391149	0.911431
Patient - 20	17-Jan-23	7	M	827.8	BCR::ABL1 (minor)		0.743864	1.738747	1.3163
Patient - 21	04-Aug-23	8	M	1841.1	BCR::ABL1 (minor)		0.080326	0.073798	0.062554
Patient - 22	04-Aug-23	7	M	207.4	BCR::ABL1 (major, p210)		0.146285	0.052142	0.044197
Patient - 23	09-Aug-23	22	M	580	BCR::ABL1 (minor)		0.95938	2.965022	2.513265
Patient - 24	15-Jun-23	41	M	352.6	BCR::ABL1 (minor)		0.229996	0.431272	0.365563
Patient - 25	26-Jun-23	24	F	1682.9	BCR::ABL1 (minor, p190)		0.257506	0.511716	0.43375
Patient - 26	21-Jul-23		M	584.6	BCR::ABL1 (minor)		0.242865	0.399165	0.338348
Patient - 27	03-Jul-23	36	M	1127.2	BCR::ABL1 (minor, p190)	Expired	1.08124	1.353078	1.14692
Patient - 28	03-Jul-23	15	F	509.5	BCR::ABL1 (minor, p190)		0.227502	0.140782	0.119333
Patient - 29	28-Nov-23	30	M	232.4	BCR::ABL1 (minor)		0.240373	0.227164	0.192553
Patient - 30	12-Oct-23	33	M	552.9	BCR::ABL1 (minor, p190)		0.206004	0.113272	0.096014
Patient - 31	11-Oct-23	42	M	218.9	BCR::ABL1 (minor)		0.188618	0.05318	0.045077
Patient - 32	05-Oct-23	34	F	518.5	BCR::ABL1 (minor)		0.593191	1.075963	0.912027
Patient - 33	15-Jun-23	38	M	345	BCR::ABL1 (minor)		0.891634	2.476214	2.098933
Patient - 34	29-Aug-23	42	M	575.2	BCR::ABL1 (minor)		0.12681	0.029104	0.02467
Patient - 35	29-Aug-23	45	M	675.9	BCR::ABL1 (minor)		0.651776	2.625849	2.22577
Patient - 36	10-Aug-23	11	M	1503.7	BCR::ABL1 (minor)		0.081913	0.168016	0.142416
Patient - 37	08-Dec-23	31	M	9.4	BCR::ABL1 (minor)		0.210969	0.380278	0.322338
Patient - 38	23-Oct-23	25	F	925.4	BCR::ABL1 (minor)		0.237062	0.236153	0.200172
Patient - 39	25-Oct-23	38	M	184.4	BCR::ABL1 (minor)		0.851991	1.404105	1.190173

Patient - 40	31-Mar-23	10	F	385.5	BCR::ABL1 (minor, p190)	Expired	0.293037	0.304295	0.257932
Patient - 41	25-Apr-24	72	F	892.4	BCR::ABL1 (major, p210)		0.180949	0.200122	0.169631
Patient - 42	16-Jul-24	2	M	867.2	BCR::ABL1 (minor, p190)		0.294085	0.325247	0.275691
Patient - 43	25-Jul-24	59	M	881.9	BCR::ABL1 (major)		0.092384	0.102173	0.086605
Patient - 44	23-Aug-24	11	M	782.2	BCR::ABL1 (minor, p190)		0.375817	0.415639	0.352311
Patient - 45	20-Feb-24	6	F	260.2	BCR::ABL1 (minor)		0.299096	0.330789	0.280389
Patient - 46	06-Feb-24	3	F	802.5	BCR::ABL1 (minor, p190)		0.048636	0.053789	0.045594
Patient - 47	01-Aug-24	52	M	399.2	BCR::ABL1 (major)		0.234206	0.259022	0.219557
Patient - 48	24-Jan-24	11	F	504.6	BCR::ABL1 (minor, p190)		0.323104	0.357341	0.302895
Patient - 49	24-Jan-24	35	F	657.8	BCR::ABL1 (minor, p190)		0.241544	0.267138	0.226437
Patient - 50	########	21	M	1446.8	BCR::ABL1 (major)		0.206023	0.227853	0.193137
Patient - 51	########	25	F	335.6	BCR::ABL1 (major, p210)		0.380797	0.421147	0.35698
Patient - 52	08-Jul-24	7	M	1085.5	BCR::ABL1 (minor)		0.250345	0.276872	0.234687
Patient - 53	08-Mar-24	16	F	487.5	BCR::ABL1 (minor, p190)		0.300437	0.332272	0.281646
Patient - 54	16-Jul-24	4	F	942.4	BCR::ABL1 (major, p210)		0.219505	0.242764	0.205776
Patient - 55	10-Oct-24	25	M	806.5	BCR::ABL1 (major, p210)		0.117403	0.129843	0.11006
Patient - 56	########	3	F	405.8	BCR::ABL1 (major, p210)		0.281031	0.310809	0.263454
Patient - 57	19-Sep-24	1	M	2102.3	BCR::ABL1 (minor)		0.197124	0.218012	0.184795
Patient - 58	20-Sep-24	39	F	1234.7	BCR::ABL1 (major, p210)		0.151817	0.167904	0.142321
Patient - 59	20-Feb-24	28	M	373.7	BCR::ABL1 (major, p210)		0.399021	0.441302	0.374064
Patient - 60	19-Sep-24	8	M	658	BCR::ABL1 (major, p210)		0.216576	0.239525	0.20303
Patient - 61	14-Mar-23	60	F	738.3	BCR::ABL1 (micro)		0.180989	0.176066	0.130582

Patient - 62	03-Mar-23	5	F	1293.3	BCR::ABL1 (major, p210)		0.323081	0.376368	0.281581
Patient - 63	23-Feb-23	20	M	230	BCR::ABL1 (minor)	Expired	0.133456	0.143273	0.091692
Patient - 64	24-Jan-23	4	F	378.1	BCR::ABL1 (major, p210)		0.128235	0.155376	0.097726
Patient - 65	13-Feb-23	82	F	1400.9	BCR::ABL1 (minor)		0.113957	0.118312	0.080071
Patient - 66	08-Mar-23	34	M	1586.9	BCR::ABL1 (micro)		0.242658	0.305815	0.157976
Patient - 67	########	18	M	934.8	BCR::ABL1 (minor)		0.16805	0.25308	0.109511
Patient - 68	21-Mar-23	39	M	211.3	BCR::ABL1 (minor)		0.46611	0.688112	0.366294
Patient - 69	22-Mar-23	32	M	512	BCR::ABL1 (minor)	Expired	0.128498	0.105786	0.083104
Patient - 70	########	33	F	564	BCR::ABL1 (minor)		0.829103	0.862043	0.617096
Patient - 71	08-Jun-23	18	M	218.1	BCR::ABL1 (minor)		0.473906	0.421443	0.284123
Patient - 72	20-Apr-23	34	F	1821.7	BCR::ABL1 (minor, p190)	Expired	0.176607	0.072386	0.080062
Patient - 73	29-Apr-23	7	M	1537.4	BCR::ABL1 (minor)	Expired	0.187179	0.058397	0.124052
Patient - 74	23-Mar-23	55	F	417.1	BCR::ABL1 (minor)	Expired	0.228925	0.169427	0.132557
Patient - 75	21-Mar-23	49	F	442.7	BCR::ABL1 (micro)		0.142915	0.065944	0.247314
Patient - 76	12-Jan-23	20	M	306.7	BCR::ABL1 (minor)		0.834863	0.946436	0.459651
Patient - 77	25-Apr-23	21	M	935.4	BCR::ABL1 (major, p210)		0.314065	0.34015	0.221643
Patient - 78	17-Jan-23	38	M	412.7	BCR::ABL1 (major, p210)		0.243987	0.218147	0.148619
Patient - 79	17-Sep-24	35	M	220.1	BCR::ABL1 (major)		0.200348	0.188161	0.131762
Patient - 80	23-Aug-24	15	M	382.3	BCR::ABL1 (minor, p190)		0.586273	0.219563	0.401105
Patient - 81	23-Jan-25	1	F	925.8	BCR::ABL1 (major, p210)		0.131269	0.128431	0.123059
Patient - 82	28-Jan-25	25	M	924.5	BCR::ABL1 (major, p210)		0.772355	0.000301	0.724048
Patient - 83	14-Feb-25	23	M	319.3	BCR::ABL1 (minor)		0.116368	0.10522	0.10909
Patient - 84	20-Feb-25	52	M	928.1	BCR::ABL1 (major, p210)		0.081575	0.113877	0.076473
Patient - 85	24-Feb-25	1.1	M	1092.9	BCR::ABL1 (minor, p190)		0.150459	0.109559	0.141048
Patient - 86	27-Feb-25	18	M	303.4	BCR::ABL1 (minor)		0.36323	0.756882	0.340512
Patient - 87	05-Jul-22	40	M	663.9	BCR::ABL1 (major)		0.191031	0.157168	0.179082

Patient - 88	04-Aug-22	25	F	70.2	BCR::ABL1 (major)	0.101591	0.187075	0.095237
Patient - 89	15-Jan-25	41	M	1427.6	BCR::ABL1 (major)	0.113854	0.122937	0.106733
Patient - 90	21-Jan-25	30	M	296.2	BCR::ABL1 (major)	0.597052	0.625155	0.559709
Patient - 91	########	47	M	149	BCR::ABL1 (major)	0.177909	0.341543	0.166782
Patient - 92	21-Sep-24	6	M	711	BCR::ABL1 (major, p210)	0.098645	0.156652	0.092475
Patient - 93	27-Apr-24	70	F	725.4	BCR::ABL1 (major, p210)	0.189003	0.140608	0.177181
Patient - 94	18-Jul-24	10	M	900.2	BCR::ABL1 (minor, p190)	0.308133	0.260814	0.28886
Patient - 95	25-Jul-24	57	M	654.7	BCR::ABL1 (major)	0.071011	0.061351	0.06657
Patient - 96	20-Feb-24	7	F	280.2	BCR::ABL1 (minor)	0.395121	0.407674	0.370408
Patient - 97	06-Feb-24	8	F	602.4	BCR::ABL1 (minor, p190)	0.077007	0.055664	0.072191
Patient - 98	01-Aug-24	57	M	298.2	BCR::ABL1 (major)	0.118591	0.239686	0.111173
Patient - 99	24-Jan-24	14	F	350.4	BCR::ABL1 (minor, p190)	0.325621	0.252812	0.305254
Patient - 100	24-Jan-24	36	F	659.8	BCR::ABL1 (minor, p190)	0.268355	0.291305	0.25157

Table S17: Each experimental group included multiple biological replicates to ensure that results reflected true biological variability. Within each biological replicate for the control group there were at least 2 technical replicates, reactions were run in triplicate to account for pipetting and machine variation. Multiple negative controls without template were also included to confirm absence of contamination or non-specific amplification.

- **Supplementary data for RT-PCR experiment (Patient validation study, n=100)**

1) Primers used for RT_ PCR run

Plate No.	Patient Samples (Only biological Replicates)	Control Samples (Both biological and technical Replicates)	SUBP - 15 cell line (Only technical replicate)
**Plate 1**	None	**5** biological replicates **(2 technical replicate/biological replicate)**	2 technical replicate
**Plate 2**	Patient samples 1–20	**2** biological replicates	1 technical replicate
**Plate 3**	Patient samples 21–40	**2** biological replicates	1 technical replicate
**Plate 4**	Patient samples 41–60	**2** biological replicates	1 technical replicate
**Plate 5**	Patient samples 61–80	**2** biological replicates	1 technical replicate
**Plate 6**	Patient samples 81–100	**2** biological replicates	1technical replicate

Table S18: Primer sequences used for quantitative real-time PCR (qRT-PCR) analysis of CREB1, GSK3A, and GSK3B. CREB1 primers were obtained from previously published studies (PMCID: PMC4496382; PLoS ONE 10.1371/journal.pone.0054261). GSK3A and GSK3B primer pairs were purchased from OriGene (NM_019884 and NM_002093, respectively). All primers were validated for SYBR Green based detection. The annealing temperature was optimized at 60°C for all targets.

Gene (Homo sapiens)	Forward Primer (5'→3')	Reverse Primer (5'→3')	Source
**CREB1 (cAMP Response Element-Binding Protein 1)**	**GCTGCCTCTGGAGACGTACAA**	**GCTAGTGGGTGCTGTGCGA**	**PMC4496382; PLoS ONE (10.1371/journal.pone.0054261)**
**GSK3A (Glycogen Synthase Kinase 3 Alpha)**	**GCAGATCATGCGTAAGCTGGAC**	**GGTACACTGTCTCGGGCACATA**	**OriGene (NM_019884)**
**GSK3B (Glycogen Synthase Kinase 3 Beta)**	**CCGACTAACACCACTGGAAGCT**	**AGGATGGTAGCCAGAGGTGGAT**	**OriGene (NM_002093)**

Ss
